# Aromaticity Tuning in Biaryl Monophosphines and Their Derivatives

**DOI:** 10.3390/molecules30194018

**Published:** 2025-10-08

**Authors:** Barbara Miroslaw, Pawel Rejmak, Izabela Dybala, Urszula Kosikowska, Sylwia Andrzejczuk, Łukasz Świątek, Kinga Salwa, Oleg M. Demchuk

**Affiliations:** 1 Department of General and Coordination Chemistry and Crystallography, Institute of Chemical Sciences, Faculty of Chemistry, Maria Curie-Sklodowska University in Lublin, 20-031 Lublin, Poland; 2 Laboratory of X-Ray and Electron Microscopy Research, Institute of Physics Polish Academy of Sciences, 02-668 Warsaw, Poland; rejmak@ifpan.edu.pl; 3 Department of Pathobiochemistry and Interdisciplinary Applications of Ion Chromatography, Medical University of Lublin, 20-093 Lublin, Poland; izabela.dybala@umlub.edu.pl; 4 Department of Pharmaceutical Microbiology, Medical University of Lublin, 20-093 Lublin, Poland; urszula.kosikowska@umlub.pl (U.K.); sylwia.andrzejczuk@umlub.pl (S.A.); 5 Department of Virology with Viral Diagnostics Laboratory, Medical University of Lublin, 20-850 Lublin, Poland; lukasz.swiatek@umlub.edu.pl (Ł.Ś.); kinga.salwa@umlub.pl (K.S.); 6 Faculty of Medicine, The John Paul II Catholic University of Lublin, 20-708 Lublin, Poland

**Keywords:** aromaticity, biaryl phosphines, phosphine oxides, ring-activating and ring-deactivating groups, HOMA, NICS, cross-coupling, phosphine ligand, hindered biaryls

## Abstract

Aromaticity tuning of biaryl monophosphines can significantly impact their catalytic performance. Biaryl monophosphines constitute a crucial class of compounds due to their potential as ligand precursors in asymmetric Pd-catalyzed cross-coupling and some other catalytic reactions. In this study, we investigate the tuning of aromaticity within a series of selected biaryl monophosphine derivatives exhibiting diverse steric and electronic properties. XRD structures and Hirshfeld surface analyses were complemented by DFT calculations. Aromaticity indices, such as geometric HOMA, HOMER, and magnetic NICS, were evaluated and correlated with ligand properties. NICS(1)zz was the most sensitive to aromaticity changes. The results showed that among the ring-activating substituents, methoxy groups were more beneficial than hydroxy ones. The hydroxy groups not only modulated the aromaticity but also induced unfavorable conformational changes of the catalyst precursors through strong inter- and intramolecular hydrogen bonding. The spatial arrangement of the P atom adjacent to the aryl ring system confers catalytic advantages by promoting the assembly of coordination compounds (catalysts) in which Pd—C bond formation occurs, yielding C,P-chelated five-membered palladacyclic structures. The hydroxy substituents blocked access to the P atom, thereby hindering catalytic performance. The studies show that even subtle changes in the monophosphine biaryl scaffold, especially aromaticity tuning should be carefully evaluated during the rational design of new efficient catalysts. The studied compounds were evaluated for their biological activity against three Gram-positive and four Gram-negative bacteria as model microorganisms. The research was supplemented by in vitro cytotoxicity evaluation.

## 1. Introduction

Aromaticity is a concept with multiple definitions, depending on its context of application, which is still under development [1,2,3,4]. Despite its fundamental role in organic chemistry, the concept of aromaticity still lacks a rigorously defined and universally accepted description [2,5,6]. A summary of commonly and less-commonly used aromaticity indicators is presented in Table S1 of the Supplementary Materials. These indicators are categorized as energetic [7,8,9,10,11], magnetic [12,13,14,15], structural [16,17,18,19,20], electronic [21,22,23,24,25,26,27,28], and experimental, including spectroscopic [29,30,31] and crystallographic types [32]. Among the magnetic indices worth mentioning is the Nucleus-Independent Chemical Shift (NICS) [12]—a magnetic index helping to evaluate aromaticity by calculating magnetic shielding at specific points relative to the ring. For example, NICS(0) is measured at the center of the ring, while NICS(1) is measured 1 Å above the ring plane. The more negative the values, the stronger the aromatic character. Among the structural indices widely used are the Harmonic Oscillator Model of Aromaticity (HOMA)—based on bond length deviations (more deformation indicates higher aromaticity: 0 nonaromatic, 1 fully aromatic) [16,17], and the Harmonic Oscillator Model of Electron Delocalization Revised (HOMER)—a modification of the HOMA index, applied especially for systems with weaker aromatic or antiaromatic character, including non-planar or heterocyclic rings [18].

In organic compounds, aromaticity affects properties such as thermodynamic stability, electronic structure, chemical reactivity, and spectroscopic behavior. To modulate this feature, various strategies can be employed, such as substituent effects, positional isomerism, heteroatom incorporation, steric modulation, application of external magnetic field, redox control, protonation/deprotonation, changes in ring size, ring fusion, solvent effects, etc. (Table 1).

Tuning the aromaticity in organic compounds may be crucial for organic synthesis, catalysis, materials science, and medicinal chemistry. Changing the aromaticity of biaryl monophosphines can significantly impact the catalytic performance of derived catalysts. Functionalization of the biaryl backbone with bulky and electron-donating substituents enhances both catalyst stability and reactivity in palladium-catalyzed cross-coupling reactions [49,50,51]. Studies report that bulky groups (e.g., cyclohexyl, isopropyl) and electron-rich substituents (e.g., methoxy, amino) improve air and moisture tolerance, suppress off-cycle palladium species, and promote the formation of monoligated palladium(0) complexes [49,52,53,54]. Ligands such as SPhos, KITPhos, Nap-Phos, BisNap-Phos, oxaphosphole-based compounds, and isopropyl BippyPhos have been shown to efficiently mediate carbon–carbon, carbon–nitrogen, and carbon–halogen couplings [49,50,51,54,55,56,57,58,59]. Doherty et al. [50] and Ikawa et al. [60] showed that the proper substitution pattern of biaryl fragments of the catalyst enhances catalytic efficiency, particularly in reactions involving aryl chlorides. Analogously, Pratap et al. [61] postulated a positive impact of increased electron density in the aromatic ring on C–N and C–C bond-forming reactions. Tuning aromaticity as an important factor in organic synthesis and materials chemistry was studied by many research groups [62,63,64,65]. Dudek et al. applied some of the aromaticity indicators (modified HOMA and NICS) to order selected α-amino acids according to their increasing aromaticity caused by different substitution. The greatest reduction in aromaticity was observed in the case of a π-electron-donating –OH group [66].

In this study, we focus on the substituent effect (substituent type and position) as a key factor in aromaticity tuning. As research materials, we selected biaryl monophosphines and corresponding oxides, compounds widely recognized as important precursors of highly active and selective ligands in Pd-catalyzed cross-coupling reactions. Our investigation intentionally focuses on phosphine oxide precursors rather than active phosphine ligands for important practical reasons. Phosphine oxides are significantly more stable than their corresponding phosphines, enabling reliable X-ray crystallographic analysis, while tertiary phosphines are air-sensitive and prone to oxidation. Critically, the reduction process (P=O → P) should preserve the molecular framework and electronic distribution in the aryl rings, allowing the aromaticity and electronic properties of these stable precursors to be translated into the active ligands. Our aromaticity analysis thus provides predictive insights for catalyst design without requiring direct catalytic testing of air-sensitive compounds.

The steric and electronic features of these ligands are of great importance in view of their catalytic performance. The phenomenon of dearomative rearrangement of palladium(II) biaryl phosphine complexes was previously studied by Milner et al. [67]. The authors demonstrated that voluminous alkyl substituents such as tert-butyl and adamantyl significantly influence the catalytic activity of Pd(II) complexes in cross-coupling reactions. Their results also indicated that changes in the aromatic character of the biaryl fragment are critical to reactivity. The issue of diverse coordination modes of biaryl monophosphine ligands induced by various structural patterns of palladium(II) complexes was analyzed by Miroslaw et al. [68]. However, a detailed investigation into how substituents modulate the aromaticity of biaryl monophosphine ligands at the molecular level remains lacking.

**Scheme 1:**
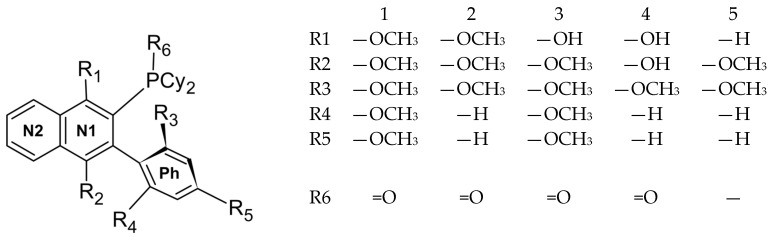
Molecular structure of the studied biaryl monophosphine derivatives (Cy—cyclohexyl).

To address this gap, we investigated a series of biaryl monophosphine derivatives variously substituted with electron-donating groups such as –OCH_3_ and –OH (Scheme 1), which may influence of the distribution of the electron density in the aromatic system.

Our study combines structural and computational analyses using single crystal X-ray diffraction, Hirshfeld surface (HS) analysis, and selected aromaticity descriptors, including geometric HOMA [16,17], HOMER [18], and magnetic NICS [12]. These findings provide new insights into the structure–aromaticity–function relationships of biaryl monophosphines, as well as corresponding phosphine oxides, and may support the rational design of next-generation ligand precursors for transition metal catalysis.

The potential application of biaryl monophosphine derivatives as catalysts in pharmaceutical synthesis poses a risk of residual catalyst contamination in final drug products. Therefore, we performed cytotoxicity and bioactivity tests to evaluate their safety profiles.

## 2. Results

### 2.1. Synthesis

**Scheme 2:**
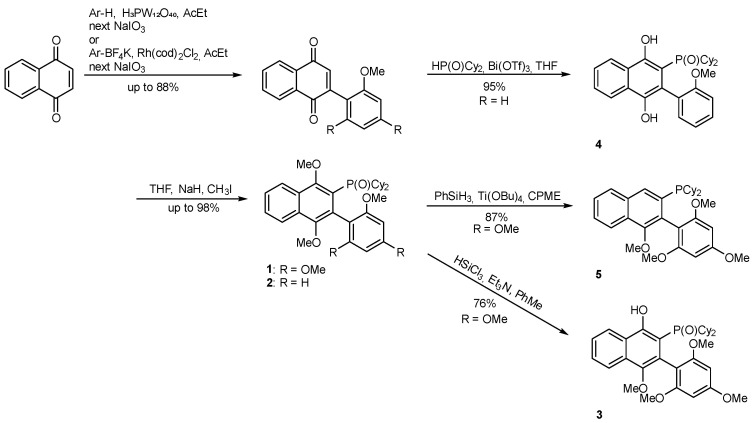
Synthesis of compounds **1**–**5**.

Compounds **1**–**5** were obtained according to Scheme 2, as previously reported for compounds **2** and **4** [59] and for compounds **1** and **5** [69]. Isolated compounds were characterized by NMR spectroscopy and single-crystal X-ray analysis.

### 2.2. Crystal Structure

**Figure 1:**
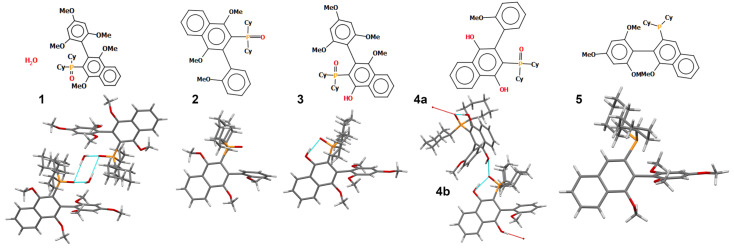
X-ray structures of compounds **1**–**5** show the orientation of the P=O vector (or the P lone pair of electrons) either toward the Ph ring (in **1**, **2**, **5**) or flipped on the opposite side, where it is stabilized by an intramolecular HB (in **3** and **4**). Crystal **1** is a monohydrate, while **4** has two symmetrically independent molecules, **4a** and **4b**.

To investigate the effect of substituent type and position on the aromatic character of selected compounds **1**–**5**, a series of derivatives bearing methoxy and hydroxy groups at different positions on the aryl rings (phenyl and naphthyl) were synthesized and structurally characterized by single crystal X-ray diffraction (Figure 1). The crystal structure refinement parameters for compounds **1**–**5** are given in Table S2.

Synthesis of **4** was previously reported by Demchuk et al. (CCDC No. 609270) but without a thorough structural analysis [59]. The list of molecular geometric parameters such as the bond length subsequently used to calculate aromaticity indices is given in Tables S3–S12. The aryl rings show a different degree of aromaticity disturbance. As expected, the bond lengths within the biaryl aromatic system show the largest deviations from the average values of the ring denoted as N1 in Scheme 1, especially for compound **4**, which contains two hydroxy substituents.

Apart from this, the molecules in the analyzed crystals exhibit one interesting feature. The orientation of the P=O vector depends on the possibility of formation of intra- or intermolecular hydrogen bonds (HB) with strong HB donors (hydroxy groups) (Figure 1, Table S13). In the crystal structures of **1** and **2**, the P=O vector is directed toward the phenyl ring. In **1**, the water molecule is small enough to occupy the position above the aromatic ring, so no flipping of the P=O bond is observed (P=O… C_Ph_ distances of ca. 2.83–2.84 Å). In **2**, the phosphine oxide group does not participate in any HB, so the conformation could be stabilized somehow by the interaction with π electrons of the phenyl ring Ph. In the case of compound **3**, an intramolecular HB with a hydroxy substituent at naphthyl N1 is formed, and the P=O vector is flipped towards the naphthyl moiety, forming the O–H…O(=P) HB. In the crystal structure of compound **4**, there are two symmetrically independent molecules. An intramolecular O–H…O(=P) HB analogous to that in structure **3** is observed. However, an additional intermolecular HB with another biaryl monophosphine oxide molecule is present. It is worth noting that the P=O vector of molecule **4a** is directed towards the aromatic phenyl ring Ph in molecule **4b** (P=O… C_Ph_ distance of 3.21 Å). In phosphine **5**, although there is no P=O bond, the vector running along the direction of P=*lp* is directed towards the aromatic ring Ph (P…C_Ph_ distance of 3.10 Å), which may be stabilized by an intramolecular interaction with the aromatic system. Summarizing, the intramolecular interactions of phosphine oxide or phosphine with the aryl Ph ring seems to influence molecular conformation in the absence of strong HB donors. An intermolecular P=O…Ph ring contact was also found in structure of compound **4**, with the O…C_Ph_ distances being 3.21 Å.

The potential flipping of the P=O group in phosphine oxides or P=*lp* vector in phosphines (which are considered basic because of electron-donating substituents and steric effect at the phosphorus atom [70]) may block access to the P atom. The strategic positioning of the phosphorus atom near the aryl Ph ring, especially in the case of phosphines, promotes catalytic performance by increasing the likelihood of forming coordination complexes (catalysts) where the Pd–C bond formation leads to C,P-chelated five-membered palladacycles [68,71].

Although two aromatic systems are present in each of the analyzed molecules, no evidence of π-stacking was observed in the crystal structures.

### 2.3. Hirshfeld Surface Analysis

**Figure 2:**
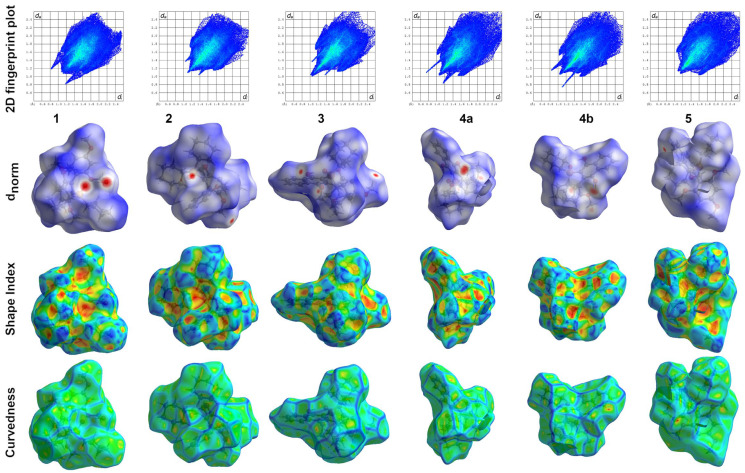
HS analysis results for **1**–**5** presented in columns: 2D fingerprint plots, d_norm_ surfaces, shape index, and curvedness. For crystal **4**, the data are presented separately for molecule **4a** and **4b**.

Hirshfeld surfaces, especially those plotted against d_norm_, along with shape index, curvedness, and fingerprint plots, helped to illustrate the differences in intermolecular interactions (Figure 2).

The main regions of shorter intermolecular contacts include the hydroxy, methoxy, and P=O groups. The presence of the –OH groups significantly increases the interaction surface in **4**, affecting solid-state packing and the accessibility of the phosphorus center. The fingerprint obtained for crystal **4** characterizes longer sharp spikes, indicating stronger O–H…O(=P) HBs. In crystal **1**, the analogous spikes are linked to the HBs formed by a water molecule. In crystal **3**, although one –OH substituent is present in the molecular structure, the sharp spikes are not so evident, and they originate from the C–H…O contacts. The hydroxy donor is used to form an intramolecular HB with the P=O acceptor. In the crystal structure of phosphine **5**, no strong HBs are formed, and the fingerprint plot is significantly different from the rest of the series (no sharp spikes). Apart from this, the wing-shaped spikes correspond to the C…H contacts. The broad central region arises from the H···H contacts. Detailed data from the HS analysis are presented in Supplementary Materials (Tables S14 and S15).

### 2.4. Aromaticity Evaluation: HOMA, HOMER, and NICS Indices

**Figure 3:**
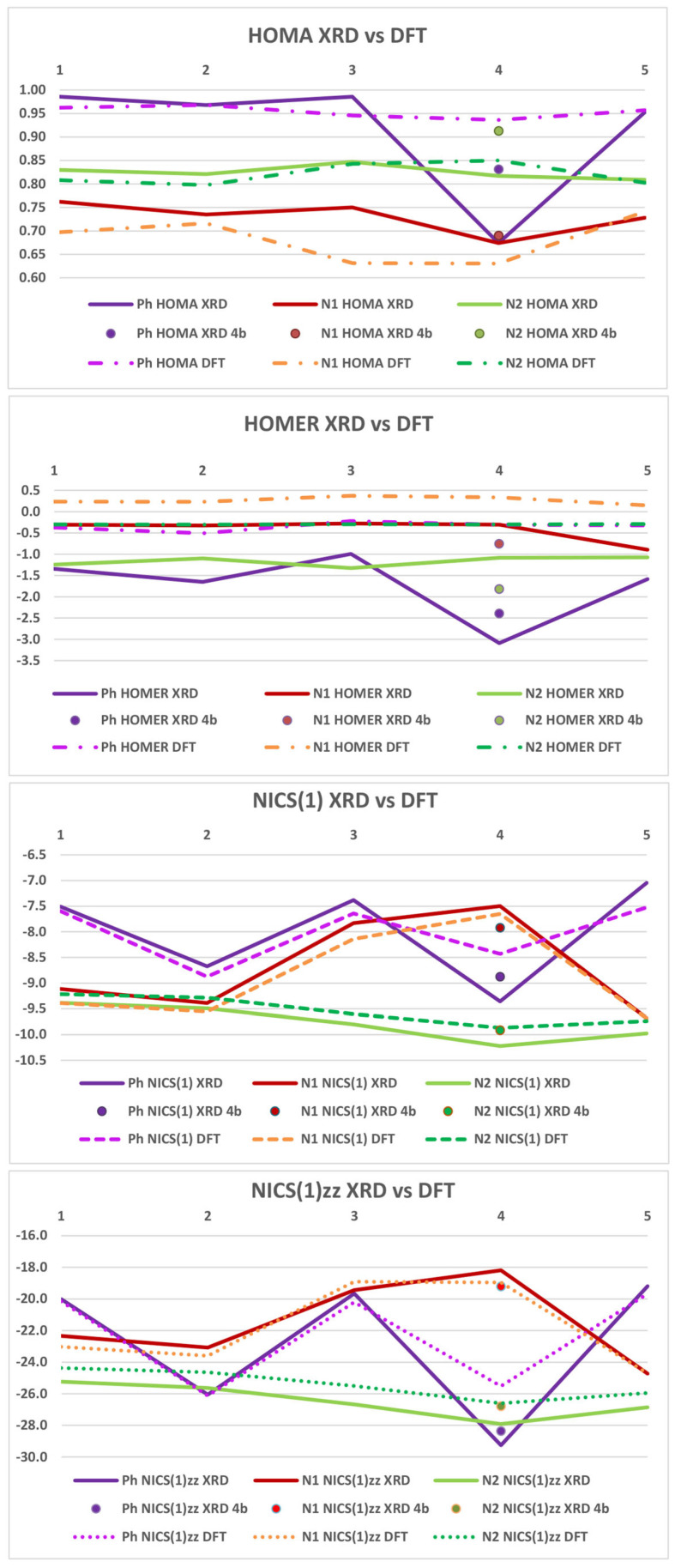
Aromaticity indices HOMA, HOMER, NICS(1), and NICS(1)zz calculated from both X-ray (XRD) and optimized (DFT) geometries for compounds **1**–**5**; in crystal **4**, the second symmetrically independent molecule is marked as **4b** (circles at the graphs). Ph, N1, and N2 denote aromatic rings, as shown in Scheme 1.

The bond length values derived from the crystal data for **1**–**5** were further analyzed to assess the aromaticity changes using the HOMA (Harmonic Oscillator Model of Aromaticity) and HOMER (Harmonic Oscillator Model of Electron Delocalization Revised) geometric indices [18,72] (Tables S16 and S17, Figure 3). For each compound, the HOMA and HOMER values were calculated separately for the aryl rings Ph, N1, and N2 (Scheme 1) using py.Aroma software ver. 4.2.0 with slight modifications (details in experimental section) [73]. These indices measure how much the aromatic molecular fragment differs from an ideal model with full delocalization of electrons.

To examine the crystal packing impact, the HOMA and HOMER indices were also recalculated for the molecular structures optimized in the gas phase by the density functional theory (DFT) method meta-gradient TPSS functional [74], identical to that used for the magnetic criterion of aromaticity (see below).

The geometric approach was complemented by the computation of the magnetic NICS (Nucleus-Independent Chemical Shift) values (NICS(0), NICS(1), and NICS(1)zz) [12] to evaluate magnetic aromaticity. The results for the X-ray (XRD) and optimized (DFT) geometries are presented in Figure 3 and Figure S6 and Table S16.

In general, substitution with electron-donating groups, such as –OH and –OR, as expected, slightly decreased aromaticity, suggesting a subtle disruption of aromatic delocalization due to increased electron density and possible conjugation with lone pairs. However, the HOMA, HOMER, and NICS indices reflect a different sequence of dearomatisation along the aryl Ph, N1, and N2 rings.

### 2.5. HOMA Indices

The HOMA indices calculated from both X-ray (XRD) and DFT-optimized data (DFT) show that the Ph ring is the least prone to loss the aromatic character in terms of geometry. The HOMA indices for Ph rings were closest to 1 (expected for fully aromatic structures) in comparison to N1 and N2 values (Ph HOMA XRD values 0.953–0.986 for **1**–**3** and **5** and Ph HOMA DFT 0.936–0.968 for structures **1**–**5**). In case of structure **4** the HOMA XRD was in the range of 0.674–0.831, but this structure refinement has the lowest quality. Crystal **4** has two symmetrically independent molecules, therefore, the number of refined parameters is higher and the bond length uncertainties are higher. This impacts the reliability of the aromaticity index calculations when based on XRD data, and additionally for this structure the difference between the indices calculated from the XRD and DFT geometries are the highest. This observation highlights the importance of validating experimental aromaticity values with computational data. The lowest aromaticity in each compound was observed for the substituted N1 part of the naphthyl fragment (N1 HOMA 0.674–0.762 (XRD) and 0.630–0.741 (DFT)). However, the DFT geometry better showed the strong impact of hydroxy substituents, lowering the aromaticity significantly in **3** and **4** (to a greater extent than in the XRD data). The aromaticity of N2 fragment was quite stable (HOMA around 0.8). The XRD and DFT data showed the same dearomatisation impact—the HOMA values (and aromatic character) decreased in the order Ph > N2 > N1.

### 2.6. HOMER Indices

In case of the HOMER index, values close to 0 indicate a nonaromatic non-delocalized structure, values close to 1 an aromatic structure (strong delocalization), and HOMER values lower than 0 antiaromatic structures (delocalization causes destabilization). All values computed from XRD geometry were negative. The values decreased in the order N1 > N2 > Ph, with N1 being closest to zero, with average values of −0.5, −1.3, and −1.8, respectively. For the DFT geometry all the HOMER indices were close to zero, but for N1 they were positive (mean value 0.3), and for Ph and N2 negative (mean value −0.3).

Interestingly, for the optimized structures, the difference between variously substituted Ph and unsubstituted N2 rings nearly disappeared, which is counterintuitive, confirming that the HOMER approach works better for molecular systems, where global dearomatisation is more important than the local structural effects caused by the substituent effect.

### 2.7. Nucleus-Independent Chemical Shift Approach (NICS)

All studied rings exhibited negative NICS(0), NICS(1), and NICS(1)zz values (Table S16, Figure 3 and Figure S6), consistent with expected aromatic character The values varied with the substituent’s type and position, indicating that π-electron delocalization is sensitive to the substitution pattern. NICS(0) is known to suffer from a strong paramagnetic contribution due to the presence of σ bonds in the plane of the ring, and is usually considered a less reliable indicator of aromaticity than the shielding values calculated above the plane, such as NICS(1) [75]. The same was observed in the studied dataset. The pattern of NICS(0) changes did not follow the substituent arrangement changes (Table S16). On the other hand, the pattern of alterations for NICS(1) and NICS(1)zz was analogous. The magnetic indices were calculated both for XRD- and DFT-optimized geometries to examine the impact of crystal packing. The highest differences between such values were observed for the Ph ring in **4**, whereas the rest of the indices followed a similar pattern of changes. Because NICS(1)zz shows the highest sensitivity to the π-electron effects and minimizes the interference from σ-bonding changes, it was chosen as the most appropriate for studying aromaticity changes upon substitution in biaryl monophosphine derivatives, as discussed in detail below.

The strongest shielding effect (NICS(1)zz), observed 1 Å above the plane of the ring (i.e., the most negative chemical shift value), implying the strongest aromaticity, is predicted for the unsubstituted naphthyl ring (N2). The N2 ring was also the least prone to aromaticity changes, with an average value of −25.4 ppm and a span of 2.2 between the maximum and minimum values. In contrast, the average and span values for the N1 and Ph rings were −21.8 ppm (span: 5.8) and −22.3 ppm (span: 6.4), respectively. The respective NICS values from the XRD geometries were very similar to the DFT ones, apart from **4** (The Ph ring has the highest structural deformations). The predicted aromaticity for each compound based on NICS(1)zz decreased as follows: N2 > N1 > Ph, which is in accordance with the expected changes, making the NICS(1)zz index the most suitable for describing subtle changes upon substitution in the studied group. The unsubstituted N2 ring should retain its aromaticity to a greater extent than the substituted rings N1 or Ph. This sequence is different compared to that in the case of the HOMA indices calculated from both the XRD and DFT geometries.

Additionally, the NICS(1)zz values showed a sensitivity to different substitution patterns within the N1 ring. A visible drop in aromaticity occurs in the naphthyl N1 ring when substituted with one or two –OH strongly activating groups (−18.9 and −19.0 ppm, for compounds **3** and **4**, respectively) comparing to methoxy substituted ones (values −23.0, −23.6, and −24.7 ppm for **1**, **2**, and **5**, respectively). This trend was not clearly visible in the case of the XRD HOMA or HOMER indices, and slightly better for DFT HOMA.

The highest fluctuations in the NICS(1)zz values were observed for the Ph ring. The NICS(1)zz values varied between −19.6 and −26.1 ppm, spanning 6.5 ppm. The number and arrangement of methoxy groups on the Ph ring are clearly reflected in the NICS(1)zz numerical trends. One —OCH_3_ group (as in compounds **2** and **4**) results in higher aromaticity (lower NICS(1)zz values), whereas the presence of three methoxy substituents (in compounds **1**, **3**, and **5**) results in a greater reduction in aromaticity. This trend was only slightly reflected in the XRD-derived HOMA and HOMER indices and was not observed for the analogous indices calculated on the DFT-optimized structures.

### 2.8. Cytotoxicity of Studied Compounds

Biaryl monophosphine derivatives are also used as catalysts in pharmaceutical synthesis. Trace amounts of these compounds may persist as process-related impurities in finished drug products. To evaluate the potential biological hazard, we performed cytotoxicity and selected bioactivity tests.

**Figure 4:**
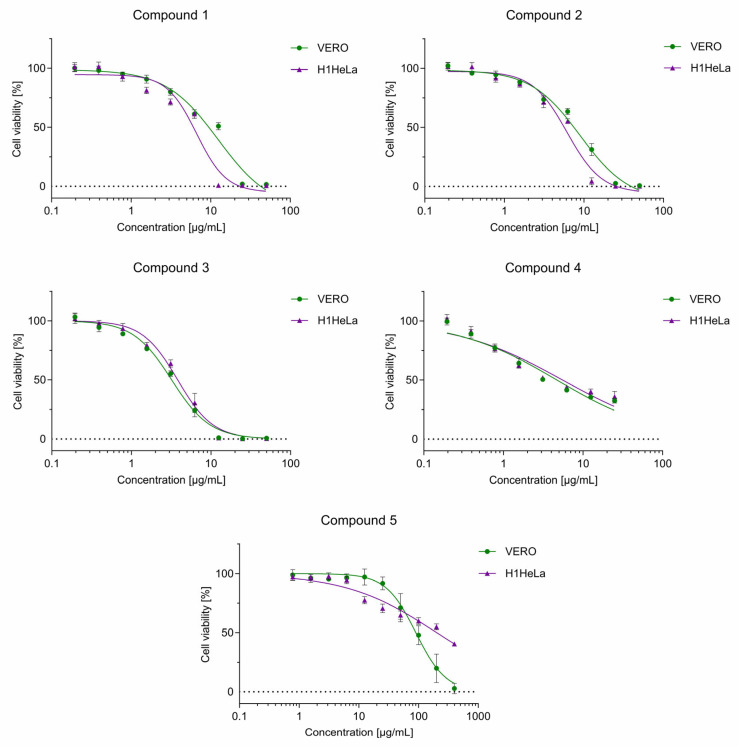
Dose–response impact of compounds **1**–**5** on cell lines VERO and H1HeLa.

The cytotoxicity of biaryl monophosphines was tested in vitro in normal (VERO) and cancer-derived (H1HeLa) cells after 24 h of incubation using a microculture tetrazolium test. Table 2 presents the CC_50_ (concentrations decreasing the cellular viability by 50%) values calculated from dose–response cytotoxicity curves (Figure 4).

**Figure 5:**
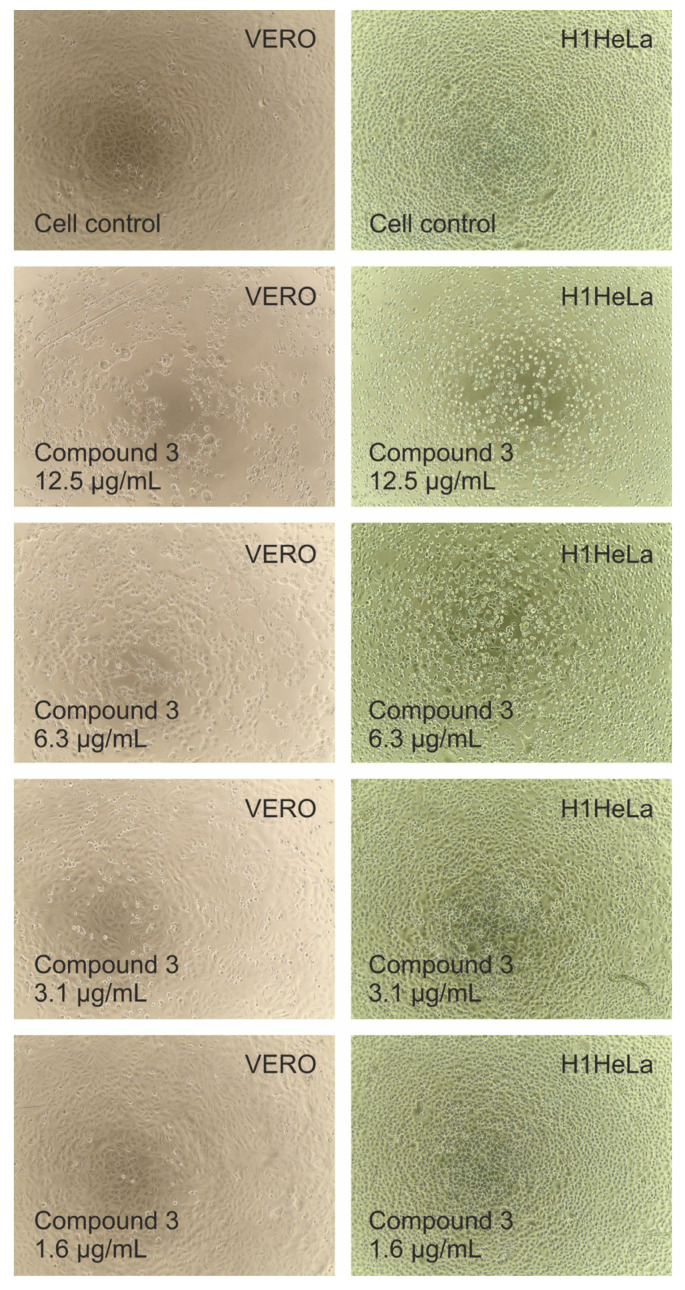
The influence of compound **3** on the morphology of VERO and H1HeLa cellular monolayers.

Based on the presented data, the highest cytotoxicity was observed for compound **3**, both toward normal (CC_50_ = 3.11 µg/mL) and cancer-derived cells (CC_50_ = 3.83 µg/mL). After 24 h of incubation, compound **3** dose-dependently influenced the morphology of the cellular monolayers of VERO and H1HeLa (Figure 5). Compound **3** at 12.5 µg/mL (Figure 5) and higher concentrations destroyed the cellular monolayer of both cell types. Only remnants of ballooned VERO cells or shrunken H1HeLa cells were observed, and the results of the MTT test revealed that these residual cells were metabolically inactive (Figure 4). When compound **3** at 6.3 µg/mL was used, some fibroblast-type cells with a spindle-shaped morphology were visible, but most of the cells showed cytotoxic damage. However, at 3.1 µg/mL, the cellular density of both cell types increased, with only a fraction of shrunken cells visible, and at 1.6 µg/mL, the cellular monolayer resembled the control cells (non-treated cells).

Compound **5** exhibited the lowest cytotoxicity among the tested compounds, with CC_50_ values of 89.88 and 200.85 µg/mL against VERO and H1HeLa, respectively. Overall, compounds **3–5** exerted noticeably higher toxicity to VERO cells compared to the effect on H1HeLa, while an opposite effect was observed for compounds **1** and **2**. Compounds **1** and **2** showed remarkable similarity in their cytotoxic impact on the tested cells. This was not only noticeable in dose–response viability curves but also in a similar influence on cellular morphology (Figure S7). Microscopic evaluation supported the results of the MTT test, indicating lower cytotoxicity of compounds **1** and **2** toward VERO cells, compared to H1HeLa. At 12.5 µg/mL of both compounds, some fibroblast-shaped VERO cells were noticeable (fewer in the case of compound **2**), while the monolayer of H1Hela was destroyed, with only cell remnants remaining. At half the concentration (6.3 µg/mL), both compounds showed similar effects on tested cells (Figure S7). The DMSO at the concentrations present in serial dilutions of the tested samples was not toxic to the cells.

### 2.9. Antibacterial Activity Screening

Because of the different structure of cell walls and biochemical properties of Gram-positive and Gram-negative bacteria [76,77,78], the antimicrobial effects of compounds **1**–**5** were tested on both types. The minimum inhibitory concentration (MIC) and minimum bactericidal concentration (MBC) values for **1**–**5** and amoxicillin (Amox) were determined and summarized in Table 3. The tested compounds exhibited low activity toward Gram-negative bacteria, with the MIC values ranging from 250 to >1000 μg/mL. An MBC value > 1000 μg/mL was determined for compounds only against the most sensitive *E. coli* ATCC 25922 species. Activity against the reference strains *S. epidermidis* ATCC 12228 (bactericidal—compounds **1** and **5**, or bacteriostatic—compounds **2**–**4**), and *S. aureus* ATCC 29213 (bacteriostatic—compounds **1**–**5**) was demonstrated. All tested compounds showed that the activity toward three examined Gram-positive reference species, with MIC values ranging from 0.98 μg/mL to 500 μg/mL and MBC values from 125 to >1000 μg/mL. Among the tested compounds, compound **2** exhibited the highest bacteriostatic activity toward Gram-positive bacteria (MICs from 1.95 to 15.63 μg/mL, MBC > 1000 μg/mL).

The most sensitive strain to the tested compounds was *M. luteus* ATCC 10240. The comparison revealed that phosphine oxides **1**, **2**, and **3**, exhibited higher antibacterial activity against *M. luteus* compared to amoxicillin, with MIC values ≤ 3.91 μg/mL versus 31.25 μg/mL obtained for amoxicillin under the same test conditions. Phosphine **5** also showed high activity against *M. luteus*, suggesting that free phosphine form may also have antibacterial properties. Our results demonstrate that these biaryl monophosphine derivatives possess promising antibacterial potential, particularly against Gram-positive bacteria. Analogous higher activity against Gram-positive than Gram-negative bacteria was observed for a series of 4-(morpholin-4-yl)-3-nitrobenzhydrazides [79]. The authors demonstrated that the presence of polarized C=O bonds appeared to enhance their activity.

## 3. Materials and Methods

### 3.1. Compounds

The NMR spectra were acquired on a JEOL ECZL500 (Jeol Ltd., 1-2, Musashino 3-Chome Akishima, Tokyo 196-8558, Japan) in DMSO-d_6_ or CDCl_3_ as a solvent and TMS (tetramethylsilane) as an internal standard. The ^1^H, ^13^C, and ^31^P NMR spectra are provided in the Supplementary Materials (Figures S8–S22). The purity of the compounds and the reaction progress were tracked by TLC using an aluminum sheet with 60 F254 plates (Merck Co., Kenilworth, NJ, USA) with a hexane/ethyl acetate/methanol solvent system. The HPLC and MS analyses were performed on a Shimadzu LC-MS Q-Tof 9030 ESI (Kyoto, Japan) instrument equipped with a column Dr. Maisch ReproSil-Pur Basic-C18 3 µm 100 mm, which was eluted at 40 °C by MeOH/H_2_O = 80/20 at a flow rate of 0.3 mL/min.

Compounds **1**, **2**, **4**, and **5** were available from previously published studies or prepared according to the procedures presented therein: compounds **2** and **4** [59], and compounds **1** and **5** [69].

Dicyclohexyl[1,4-dimethoxy-3-(2,4,6-trimethoxyphenyl)naphthalen-2-yl]phosphane oxide (**1**). ^31^P NMR (202 MHz, CDCl_3_): *δ* = 49.60. ^1^H NMR (500 MHz, CDCl_3_): *δ* = 1.17–2.18 (m, 22 H), 3.59 (s, 3H), 3.66 (s, 6H), 3.86 (s, 3H), 4.07 (s, 3H), 6.17 (s, 2H), 7.52–7.58 (m, 2H), 8.08 (d, J = 8.20, 1H), 8.16 (d, J = 8.20, 1H). ^13^C dept NMR (75 MHz, CDCl_3_): *δ* = 26.4–27.3 (Cy), 39.3 (d, J = 66 Hz), 55.3, 55.5, 61.5, 62.7, 62.7, 90.3, 123.9, 123.9, 124.0, 126.0, 127.4. CCDC No. 2482474.

Dicyclohexyl[1,4-dimethoxy-3-(2-methoxyphenyl)naphthalen-2-yl]phosphane oxide (**2**). ^31^P NMR (202 MHz, CDCl_3_): *δ* = 47.10. ^1^H NMR (500 MHz, CDCl_3_): *δ* = 1.13–2.26 (m, 22 H), 3.53 (s, 3H), 7.72 (s, 3H), 4.07 (s, 3H), 6.91 (d, J = 8,0 Hz, 1H), 6.97 (t, J = 8.60 Hz, 1H), 7.04 (dd, J = 7.40, 1.70 Hz, 1H), 7.35 (td, J = 7.70, 1.70 Hz, 1H), 7.56–7.63 (m, 2H), 8.11 (d, J = 8.0 Hz, 1H), 8.16 (d, J = 8.6 Hz, 1H). ^13^C dept NMR (75 MHz, CDCl_3_): *δ* = 25.7–26.9 (Cy), 38.9 (d, J *=* 66 Hz), 39.1 (d, J *=* 66 Hz), 55.1, 61.5, 62.8, 109.7, 119.5, 123.7, 126.2, 127.8, 128.8, 130.6. CCDC No. 2482475.

2-(Dicyclohexylphosphoryl)-3-(2-methoxyphenyl)naphthalene-1,4-diol (**4**). ^31^P NMR (202 MHz, CDCl_3_): *δ* = 67.93. ^1^H NMR (500 MHz, CDCl_3_): *δ* = 0.87–1.70 (m, 22H), 3.69 (s, 3H), 7.04–7.15 (m, 3H), 7.46–7.55 (m, 2H), 7.62 (t, J = 6.10 Hz, 1H), 7.97 (bs, 1H), 8.09 (d, J = 8.10 Hz, 1H), 8.19 (d, J = 8.10 Hz, 1H). ^13^C dept NMR (75 MHz, CDCl_3_): *δ* = 25.6–26.9 (Cy), 38.4 (d, J = 64 Hz), 38.1 (d, J = 64 Hz), 55.8, 111.6, 120.6, 122.6, 123.2, 126.1, 128.3, 130.5, 131.9. CCDC No. 609270.

Dicyclohexyl[1,4-dimethoxy-3-(2,4,6-trimethoxyphenyl)naphthalen-2-yl]phosphane (**5**). ^31^P NMR (202 MHz, C_6_D_6_): *δ* = −8.87. ^1^H NMR (500 MHz, C_6_D_6_): *δ* = 1.09–1.87 (m, 22H), 3.66 (s, 6H), 3.60 (s, 3H), 3.89 (s, 3H), 6.22 (s, 2H), 7.48–7.51 (m, 2H), 7.81–7.82 (m, 1 H), 7.89–7.91 (m, 1H), 8.14–8.16 (m, 1H). ^13^C dept NMR (75 MHz, C_6_D_6_): *δ* = 27.1–30.5 (Cy), 34.9 (d, J = 17 Hz), 54.6, 55.0, 55.2, 60.9, 90.7, 123.1, 126.1, 128.2, 128.3, 128.6, 128.8. CCDC No. 2482477.

Compound **3** was prepared as follows:

2-(Dicyclohexylphosphoryl)-4-methoxy-3-(2,4,6-trimethoxyphenyl)naphthalen-1-ol (**3**). A 250 mL 2 neck dry flask, equipped with a stir bar, reflux condenser and septum, was charged with 7.2 g of **1**, 11 mL of Et_3_N, and 100 mL of toluene. The reflux condenser was connected to an argon line, and the reaction mixture was maintained under an argon atmosphere. With efficient stirring, 2.6 mL of HSiCl_3_ was slowly added. The flask was placed in an oil bath, and the reaction was stirred under reflux for 16 h. Next, it was allowed to cool down to ambient temperature. The reaction mixture was poured on crushed ice. Organic compounds were extracted with ethyl acetate. The organic phase was separated and dried over the magnesium sulphate. After filtration, the solvents were evaporated under reduced pressure, and the product was crystallized from cyclopentyl methyl ether to yield 5.5 g (79%) of **3**. ^31^P NMR (202 MHz, CDCl_3_): *δ* = 67.62. ^1^H NMR (500 MHz, CDCl_3_): *δ* = 1.02–1.23 (m, 6H), 1.48–1.84 (m, 16H), 3.52 (s, 3H), 3.71 (s. 6H), 3.93 (s, 3H), 6.25 (s, 2H), 7.5 (ddd, J = 8.20, 6.94, 1.26 Hz, 1H), 7.58 (ddd, 8.20, 6.90, 1.30 Hz, 1H), 7.97 (d, J = 8.20 Hz, 1H), 8.42 (d, J = 8.20 Hz, 1H). ^13^C dept NMR (75 MHz, CDCl_3_): *δ* = 25.8–26.9 (Cy), 38.25 (d, J = 61 Hz), 55.6, 55.7, 61.0, 90.4, 122.1, 124.1, 125.5, 128.3. HRMS (ESI): m/z = calcd. 553.27135 (M+H+, C_32_H_41_O_6_P), found. 553.27127, diff. 0.149 ppm. CCDC No. 2482476.

### 3.2. X-Ray Crystallography

The diffraction experiments were performed on a SuperNova Orford single-crystal diffractometer. Copper anode (CuKα λ = 1.54184 Å) was applied. Details on data collecting, reduction, and refinement are given in Table S2. Olex2 was used for structure determination and refinement [80]. The non-H atoms were refined anisotropically. H atoms were added from the Fourier difference electron density maps. Crystallographic data for **1**–**3** and **5** were deposited in the CSD database under CCDC No. 2482474–2482477. Data for **4** were previously deposited under CCDC No. 609270 and previously published but not analyzed in detail [59]. Data for **5** were retrieved from a twinned crystal, and the second component was rotated by 180°.

### 3.3. Hirshfeld Surface Analysis

Hirshfeld surface analysis was performed for the crystal structures of **1–5** by using CrystalExplorer 21.5 [81]. The input data were taken from single-crystal X-ray diffraction. The wave functions were generated using the Gaussian DFT (B3LYP) method with the 6-311G(d,p) basis set [82]. The 3D d_norm_ surfaces, shape index, curvedness, and 2D fingerprint plots were generated by the same software.

### 3.4. HOMA and HOMER Calculations

The HOMA and HOMER indices were calculated and visualized using py.Aroma software [73]. The source code ver. 4.2.0 was slightly modified for better P atom connectivity presentation. The geometry was taken from the X-ray diffraction results, as well as from the optimized geometry (details below).

### 3.5. Nucleus-Independent Chemical Shift Approach (NICS)

The density functional theory (DFT) calculations were performed using the ORCA code [83], employing meta-gradient TPSS functional [74] and polarized triple-zeta Gaussian basis sets [84,85]. The initial geometries of the studied compounds were taken from the crystallographic data presented in this work and optimized at DFT level. The resulting geometries, which were found to be very similar to the experimental ones, were subsequently used in NICS(1) calculations. The results are summarized in Table S16 and Figure 3 and Figure S6.

### 3.6. Cytotoxicity Evaluation

Cytotoxicity was evaluated using a microculture tetrazolium test as previously described [86], and details can be found in the Supplementary Materials. The cell lines used in the presented study were acquired from the American Type Culture Collection (ATCC); the one used for cytotoxicity testing included non-cancerous VERO (kidney; ATCC CCL-81) cells and cancer-originating H1HeLa (cervical adenocarcinoma; ATCC CRL-1958) cells. VERO cells were cultured in Dulbecco’s Modified Eagle’s Medium (DMEM; Corning, Tewksbury, MA, USA), while Eagle’s Minimum Essential Medium (MEM; Corning) was used for H1HeLa. Antibiotics (penicillin–streptomycin solution, Corning) and fetal bovine serum (FBS, Corning) were added to cell media. Cells were incubated in a 5% CO_2_ atmosphere at 37 °C (CO_2_ incubator, Panasonic Healthcare Co., Tokyo, Japan). Briefly, the monolayers of VERO or H1HeLa cells in 96-well plates (Falcon, Corning) were treated with serial dilutions of selected compounds for 24 h. After this, the medium was removed, the plates were washed with phosphate buffered saline (PBS; Corning), a 10% solution of (3-(4,5-dimethylthiazol-2-yl)-2,5-diphenyltetrazolium bromide (MTT; Sigma-Aldrich, St. Louis, MO, USA) (5 mg/mL) in FBS-free cell media was added, and the incubation continued for the next 4 h. Subsequently, the precipitated formazan crystals were dissolved, and after overnight incubation at 37 °C, the Synergy H1 Multi-Mode Microplate Reader (BioTek Instruments, Inc., Winooski, VT, USA) with Gen5 software (ver. 3.09.07; BioTek Instruments, Inc.) was used for absorbance acquisition (540 and 620 nm). Data was exported to GraphPad Prism (ver. 10) to calculate the CC_50_ values (50% cytotoxic concentration) from dose–response curves.

### 3.7. Antibacterial Activity Screening

The antimicrobial activities of a series of compounds **1**–**5** were tested through the utilization of seven reference species, encompassing both Gram-positive (*Staphylococcus aureus* ATCC 29213, *Staphylococcus epidermidis* ATCC 12228, *Micrococcus luteus* ATCC 10240) and Gram-negative (*Escherichia coli* ATCC 25922, *Klebsiella pneumoniae* ATCC 13883, *Proteus mirabilis* ATCC 12453, *Acinetobacter baumannii* ATCC 19606) bacteria as model microorganisms. For this purpose, microbial suspensions were prepared in sterile saline (0.85% NaCl) with an optical density of 0.5 McFarland standard, yielding a concentration of 150 × 10^6^ CFU/mL (CFU, colony-forming units). All of the stock solutions of the tested compounds were dissolved in DMSO. Amoxicillin (Glentham Life Sciences Ltd., Corsham, UK) was used as a positive control.

The MIC values of the tested compounds were determined by serial twofold dilutions of **1**–**5** in Mueller Hinton Broth (MHB, Biomaxima, Lublin, Poland). The range of final **1**–**5** concentrations used was from 0.49 up to 1000 μg/mL, and for amoxicillin from 0.12 up to 250 μg/mL in the 96-well microtiter plates (Medlab, Raszyn, Poland), starting with 98 μL MHB and 2 μL bacterial inoculum added to each well. Positive controls consisted of 100 μL MHB in each well with the tested compounds at the same concentration range. The negative controls consisted of 100 μL of MHB only. Each set of tests and controls was established with three replicates. The microtiter plates were then subjected to an incubation period of 24 h at a temperature of 35 ± 2 °C. After incubation, the optical density of the microbial growth was measured at a wavelength of 600 nm using a microplate reader, the Biotek Elx800 (BioTek Instruments, Inc., Winooski, VT, USA). The MIC value was defined as the lowest concentration of the tested compound at which there was no visible growth of the tested microorganisms. The determination of the MIC values was achieved by employing the broth microdilution method, in accordance with the recommendations of the European Committee on Antimicrobial Susceptibility Testing (EUCAST) [87]. Following MIC and MBC determination, 5 μL from each well that displayed no growth signs was transferred onto MHA (Mueller Hinton Agar, Biomaxima, Lublin, Poland) plates. Then, the MHA plates were incubated overnight at 35 ± 2 °C. The MBC was defined as the lowest concentration of the compound that resulted in a >99.9% reduction in CFU of the initial inoculum. The MIC and MBC values were reported in μg/mL, according to the EUCAST reference.

## 4. Conclusions

This study demonstrates that tuning aromaticity through substituent modification is a valuable strategy in the rational design of biaryl monophosphine derivatives with potential catalytic application. We want to emphasize the following key findings.

Substituent effects: Methoxy groups are preferable over hydroxy substituents for designing biaryl monophosphine-derived catalysts, for both catalytic and biological reasons. Methoxy substitution decreases the aromaticity of the Ph ring, therefore favoring Pd-Ph interactions that are crucial for catalytic activity. Hydroxy groups, while decreasing aromaticity, form strong intramolecular hydrogen bonds that cause unfavorable conformational changes, blocking access to the P atom and hindering potential catalyst formation. Hydroxy-substituted compounds exhibit higher cytotoxicity, making them less desirable and limiting their practical utility.

Aromaticity descriptors: Among the aromaticity indices evaluated, NICS(1)zz proved to be the most sensitive for detecting electronic changes upon substitution in biaryl monophosphine motifs. The HOMA indices, calculated from both the XRD and DFT geometries, showed minimal crystal packing effects and provided consistent aromaticity sequences (Ph > N2 > N1), reflecting σ-framework thermodynamic stabilization. HOMER proved unsuitable for studying local substitution effects.

Methodological recommendation: The optimal approach combines geometric (HOMA) and magnetic (NICS) indices, which provide complementary information about π-electron delocalization and structural stabilization. Such a combination enables the validation of experimental aromaticity values using computational data.

Bioactivity: All compounds demonstrated greater bacteriostatic efficacy against Gram-positive versus Gram-negative bacteria, particularly against staphylococci. Additionally, compounds **1**, **2**, **3**, and **5** exhibited interesting activity against *M. luteus* compared to amoxicillin (MIC ≤ 3.91 μg/mL).

Substituent-induced aromaticity changes in the studied biaryls led to subtle framework modifications that significantly influence the accessibility of the P atom and potentially the catalytic efficiency. These findings provide practical guidelines for designing efficient biaryl monophosphine catalyst precursors.

## Figures and Tables

**Table 1:** Aromaticity modulation methods.

Modulation Method	Effect on Aromaticity	Ref.
Substituent effects	π-Density is modulated by electron-donating (lowering) or -withdrawing groups (increasing of aromaticity).	[33,34,35,36]
Protonation/ deprotonation	Changes π-electron count, affects Hückel/Möbius character.	[37,38]
Redox modulation	Alters number of π-electrons.	[12]
Structural constraints	Strain, ring size or planarity influence electron delocalization.	[39]
Heteroatom incorporation	Heteroatoms modulate π-electron count, delocalization, and aromatic stabilization via lone pair donation or withdrawal.	[40]
Ring size and fusion	Ring size affects planarity and π-delocalization; fusion alters local versus global aromaticity.	[41]
Metal coordination	Metal centers alter electron density, affecting aromaticity.	[42,43]
Solvent/pH effects	Environmental factors influence tautomerism or charge states.	[44,45,46]
Excited states (Baird’s rule)	Reverses aromatic or antiaromatic behavior in triplet states.	[47,48]

**Table 2:** Cytotoxicity of compounds **1–5**.

Compound	CC_50_ (µg/mL) *	
	VERO	H1HeLa
1	9.82 ± 0.59	6.78 ± 0.27
2	7.78 ± 0.44	5.92 ± 0.40
3	3.11 ± 0.14	3.83 ± 0.33
4	4.70 ± 0.41	5.53 ± 0.12
5	89.88 ± 4.11	200.85 ± 13.08

* CC_50_ (µg/mL) ± SD, calculated from dose–response curves for VERO and H1HeLa.

**Table 3:** In vitro activities of compounds **1**–**5** and amoxicillin (Amox) against Gram-positive and Gram-negative bacteria, expressed as minimum inhibitory concentrations (MICs, μg/mL) and minimum bactericidal concentrations (MBCs, μg/mL).

	1		2		3		4		5		Amox	
Bacterial Strain	MIC	MBC	MIC	MBC	MIC	MBC	MIC	MBC	MIC	MBC	MIC	MBC
*S. aureus* ATCC 29213	15.63–31.25	>1000	7.82–15.63	>1000	62.5–125	>1000	250	>1000	125–250	>1000	0.98	0.98
*S. epidermidis* ATCC 12228	15.63–31.25	250	3.91–7.89	>1000	31.25	>1000	500	>1000	31.25–62.5	1000	31.25	31.25
*M. luteus* ATCC 10240	1.95–3.91	125	1.95	>1000	0.98	>1000	15.63–31.25	>1000	1.95–3.51	125	31.25	31.25
*E. coli* ATCC 25922	500–1000	nd	>1000	nd	250–500	>1000	500–1000	nd	>1000	nd	3.91	7.81
*K. pneumoniae* ATCC 13883	>1000	nd	>1000	nd	500	nd	500	nd	>1000	nd	250	250
*P. mirabilis* ATCC 12453	>1000	nd	>1000	nd	>1000	nd	1000	nd	>1000	nd	0.49	0.49
*A. baumannii* ATCC 19606	1000/1000	nd	500/500	nd	>1000	nd	1000	nd	1000	nd	125	125

## Data Availability

Dataset available on request from the authors.

## Supplementary Materials

The following supporting information can be downloaded at https://www.mdpi.com/article/10.3390/molecules30194018/s1. Table S1. Selected aromaticity indicators. Table S2. Crystal data and structure refinement for **1**–**5**. The X-ray structure of **4** was previously reported. Figure S1. Molecular structure of 1. Ellipsoids with 50% probability. Table S3. Bond lengths for 1. Table S4. Bond angles for **1**. Figure S2. Molecular structure of **2**. Ellipsoids with 50% probability. Table S5. Bond lengths for **2**. Table S6. Bond angles for **2**. Figure S3. Molecular structure of **3**. Ellipsoids with 50% probability. Table S7. Bond lengths for **3**. Table S8. Bond angles for **3**. Figure S4. Molecular structure of **4**. Ellipsoids with 50% probability. Table S9. Bond lengths for **4**. Table S10. Bond angles for **4**. Figure S5. Molecular structure of **5**. Ellipsoids with 50% probability. Table S11. Bond lengths for **5**. Table S12. Bond angles for **5**. Table S13. Intra- and intermolecular contacts in **1**–**5**. Table S14. Hirshfeld surface analysis results for **1**–**5**: 2D fingerprint plots, d_norm_ surfaces, shape index and curvedness. Table S15. Contributions to Hirshfeld surface area for various contacts in **1**–**5**. Table S16. The geometric aromaticity indices HOMA and HOMER, calculated from the X-ray (XRD) and optimized (DFT) structures, as well as the magnetic indices NICS(0), NICS(1), and NICS(1)zz. Figure S6. The aromaticity index NICS(0), calculated from X-ray (XRD) and optimized (DFT) geometries for compounds **1**–**5**. Table S17. Visualization of aromaticity indices HOMA and HOMER calculated from X-ray (XRD) and optimized (DFT) geometries. Cell line maintenance and in vitro experiments. Evaluation of cytotoxicity. Figure S7. The influence of compounds **1** and **2** on the morphology of VERO and H1HeLa cellular monolayer. (VERO—normal kidney cells; H1HeLa—cervical adenocarcinoma). Figure S8. The ^1^H NMR spectrum for compound **1**. Figure S9. The ^13^C NMR spectrum for compound **1**. Figure S10. The ^31^P NMR spectrum for compound **1**. Figure S11. The ^1^H NMR spectrum for compound **2**. Figure S12. The ^13^C NMR spectrum for compound **2**. Figure S13. The ^31^P NMR spectrum for compound **2**. Figure S14. The ^1^H NMR spectrum for compound **3**. Figure S15. The ^13^C NMR spectrum for compound **3**. Figure S16. The ^31^P NMR spectrum for compound **3**. Figure S17. The ^1^H NMR spectrum for compound **4**. Figure S18. The ^13^C NMR spectrum for compound **4**. Figure S19. The ^31^P NMR spectrum for compound **4**. Figure S20. The ^1^H NMR spectrum for compound **5**. Figure S21. The ^13^C NMR spectrum for compound **5**. Figure S22. The ^31^P NMR spectrum for compound **5**.
