# Supplementary material for: Aromaticity Tuning in Biaryl Monophosphines and Their Derivatives
Source: Molecules. 2025 Oct 8;30(19):4018. doi: 10.3390/molecules30194018 (PMC12525867; doi:10.3390/molecules30194018)

Supplementary Materials

Aromaticity Tuning in Biaryl Monophosphines and Their Derivatives

Barbara Miroslaw [1,*], Pawel Rejmak [2], Izabela Dybala [3], Urszula Kosikowska [4], Sylwia Andrzejczuk [4], Łukasz Świątek [5], Kinga Salwa [5], and Oleg M. Demchuk [6,*]

[1] Department of General and Coordination Chemistry and Crystallography, Institute of Chemical Sciences, Faculty of Chemistry, Maria Curie-Sklodowska University in Lublin, 20-031 Lublin, Poland
[2] Laboratory of X-Ray and Electron Microscopy Research, Institute of Physics Polish Academy of Sciences, 02-668 Warsaw, Poland; rejmak@ifpan.edu.pl
[3] Department of Pathobiochemistry and Interdisciplinary Applications of Ion Chromatography, Medical University of Lublin, 20-093 Lublin, Poland; izabela.dybala@umlub.edu.pl
[4] Department of Pharmaceutical Microbiology, Medical University of Lublin, 20-093 Lublin, Poland; urszula.kosikowska@umlub.pl (U.K.); sylwia.andrzejczuk@umlub.pl (S.A.)
[5] Department of Virology with Viral Diagnostics Laboratory, Medical University of Lublin, 20-850 Lublin, Poland; lukasz.swiatek@umlub.edu.pl (Ł.Ś.); kinga.salwa@umlub.pl (K.S.)
[6] Faculty of Medicine, The John Paul II Catholic University of Lublin, 20-708 Lublin, Poland
* Correspondence: barbara.miroslaw@mail.umcs.pl (B.M.); oleh.demchuk@kul.lublin.pl (O.M.D.)

Keywords: aromaticity; biaryl phosphines; phosphine oxides; ring-activating and ring-deactivating groups; HOMA; NICS; cross-coupling; phosphine ligand; hindered biaryls

Table S1. Selected aromaticity indicators. 3
Table S2. Crystal data and structure refinement for 1-5. X-ray structure of 4 was reported previously [27]. 5
Figure S1. Molecular structure of 1. Ellipsoids with 50% probability. 6
Table S3. Bond lengths for 1. 7
Table S4. Bond angles for 1. 7
Figure S2. Molecular structure of 2. Ellipsoids with 50% probability. 8
Table S5. Bond lengths for 2. 8
Table S6. Bond angles for 2. 9
Figure S3. Molecular structure of 3. Ellipsoids with 50% probability. 10
Table S7. Bond lengths for 3. 10
Table S8. Bond angles for 3. 11
Figure S4. Molecular structure of 4. Ellipsoids with 50% probability. 12
Table S9. Bond lengths for 4. 12
Table S10. Bond angles for 4. 13
Figure S5. Molecular structure of 5. Ellipsoids with 50% probability. 15
Table S11. Bond lengths for 5. 15
Table S12. Bond angles for 5. 16
Table S13. Intra- and intermolecular contacts in 1-5. 17

Table S14. Hirshfeld Surface analysis results for 1-5: 2D fingerprint plots, dnorm surfaces, shape index and curvedness. 18

Table S15. Contributions to Hirshfeld surface area for various contacts in 1-5........ 19

Table S16. Geometric aromaticity indices HOMA and HOMER calculated for X-ray (XRD) and optimized (DFT) structures and magnetic indices NICS(0), NICS(1), and NICS(1)zz. 20

Figure S6. Aromaticity index NICS(0) calculated for X-ray (XRD) and optimized (DFT) geometries for compounds 1-5. 21

Table S17. Visualization of aromaticity indices HOMA and HOMER calculated for X-ray (XRD) and optimized (DFT) geometry. 22

Cell line maintenance and *in vitro* experiments 28

Evaluation of cytotoxicity 28

Figure S7. The influence of compounds 1 and 2 on the morphology of VERO and H1HeLa cellular monolayer. (VERO –normal kidney cells; H1HeLa - cervical adenocarcinoma). 30

Figure S8. The ^{1}H NMR spectrum for compound 1. 31

Figure S9. The ^{13}C NMR spectrum for compound 1. 32

Figure S10. The ^{31}P NMR spectrum for compound 1. 33

Figure S11. The ^{1}H NMR spectrum for compound 2. 34

Figure S12. The ^{13}C NMR spectrum for compound 2. 35

Figure S13. The ^{31}P NMR spectrum for compound 2. 36

Figure S14. The ^{1}H NMR spectrum for compound 3. 37

Figure S15. The ^{13}C NMR spectrum for compound 3. 38

Figure S16. The ^{31}P NMR spectrum for compound 3. 39

Figure S17. The ^{1}H NMR spectrum for compound 4. 40

Figure S18. The ^{13}C NMR spectrum for compound 4. 41

Figure S19. The ^{31}P NMR spectrum for compound 4. 42

Figure S20. The ^{1}H NMR spectrum for compound 5. 43

Figure S21. The ^{13}C NMR spectrum for compound 5. 44

Figure S22. The ^{31}P NMR spectrum for compound 5. 45

Table S1. Selected aromaticity indicators.

Category	Indicator	Description	Ref.
Energetic	Resonance Energy (RE)	Difference between actual and hypothetical non-aromatic energy.	[1,2]
	Aromatic Stabilization Energy (ASE)	Energy gain due to aromatic delocalization.	[3]
	Isomerization Stabilization Energy (ISE)	Energy difference between aromatic and non-aromatic isomers.	[4]
	Stabilization Energy by Substituent Exchange (SESE)	Stabilization comparison via substituent exchange.	[5]
Magnetic	Nucleus-Independent Chemical Shift (NICS)	Evaluation of aromaticity by calculating magnetic shielding at specific points in the ring, NICS(0) - at the center of the ring, NICS(1) - 1 Å above the ring plane. The more negative values the stronger aromatic character.	[6]
	Anisotropy of the Induced Current Density (ACID)	Computational method used to visualize electronic delocalization in molecules by showing the induced ring current density.	[7]
	Iso-chemical Shielding Surfaces (ICSS)	Mapping magnetic shielding regions in molecules.	[8]
	Gauge Including Magnetically Induced Currents (GIMIC)	Quantitative evaluation of magnetically induced ring currents.	[9]
Structural	Harmonic Oscillator Model of Aromaticity (HOMA)	Based on bond length deviations. The more deformation the higher aromaticity (0 nonaromatic, 1 fully aromatic)	[10,11]
	Harmonic Oscillator Model of Electron Delocalization Revised (HOMER)	Modification of HOMA index, applied especially for systems with weaker aromaticity or antiaromatic including non-planar or heterocyclic rings.	[12]
	Bird Index (Ia)	Quantification of the degree of bond length changes within a ring using bond orders instead of bond lengths.	[13]
	Bond Length Alternation (BLA)	Variation in bond lengths between single and multiple bonds within a conjugated part of a molecule.	[14]
Electronic	Para-Delocalization Index (PDI)	Quantification of π-electron delocalization between para substituted atoms.	[15,16]
	Fluctuation Index (FLU)	Measuring uniformity of π-electron distribution. In contrast to PDI, it can be used to rings with any number of atoms.	[17]
	Multicenter bond Index (MCI)	Evaluation of multicenter electron sharing involving the σ and π electrons, related to energetic and magnetic criteria.	[18,19]
	Electron Density of Delocalized Bonds (EDDB)	Visualization and quantification of delocalized electron density within multicenter bonding patterns.	[20]
	Electron Localization Function (ELF)	Visualization of localized versus delocalized electron regions.	[21]
	Localized Orbital Locator (LOL)	Alternative to ELF for delocalization mapping based on orbital kinetic energy density.	[22]
Experimental	UV-Vis absorption shift	Spectroscopic shifts linked to π-conjugation.	[23]
	IR fingerprint	Characteristic vibrational modes of conjugated systems.	[24,25]

X-ray bond length equalization	Crystallographic observation of bond lengths changes.	[26]

References

1. Rashid, Z.; Van Lenthe, J. H.; Havenith, R. W. A. Resonance and aromaticity: An ab initio valence bond approach. *J. Phys. Chem. A* **2012**, *116* (19), 4778–4788.
2. Pauling, L.; Wheland, G. W. The Nature of the Chemical Bond. V. The Quantum-Mechanical Calculation of the Resonance Energy of Benzene and Naphthalene and the Hydrocarbon Free Radicals. *J. Chem. Phys.* **1933**, *1* (6), 362–374.
3. Schleyer, P. V. R.; Jiao, H. What is aromaticity? *Pure Appl. Chem.* **1996**, *68* (2), 209–218.
4. Von Ragué Schleyer, P.; Manoharan, M.; Wang, Z. X.; Kiran, B.; Jiao, H.; Puchta, R.; Van Eikema Hommes, N. J. R. Dissected nucleus-independent chemical shift analysis of π-aromaticity and antiaromaticity. *Org. Lett.* **2001**, *3* (16), 2465–2468.
5. Krygowski, T. M.; Cyrañski, M. K.; Czarnocki, Z.; Häfelinger, G.; Katritzky, A. R. Aromaticity: a Theoretical Concept of Immense Practical Importance. *Tetrahedron* **2000**, *56* (13), 1783–1796.
6. Chen, Z.; Wannere, C. S.; Corminboeuf, C.; Puchta, R.; Von, P.; von Ragué Schleyer, P. Nucleus-Independent Chemical Shifts (NICS) as an Aromaticity Criterion. *Chem. Rev.* **2005**, *105*, 3842–3888.
7. Geuenich, D.; Hess, K.; Köhler, F.; Herges, R. Anisotropy of the induced current density (ACID), a general method to quantify and visualize electronic delocalization. *Chem. Rev.* **2005**, *105* (10), 3758–3772.
8. Klod, S.; Kleinpeter, E. Ab initio calculation of the anisotropy effect of multiple bonds and the ring current effect of arenes—application in conformational and configurational analysis. *J. Chem. Soc. Perkin Trans. 2* **2001**, *1* (10), 1893–1898.
9. Fliegl, H.; Taubert, S.; Lehtonen, O.; Sundholm, D. The gauge including magnetically induced current method. *Phys. Chem. Chem. Phys.* **2011**, *13* (46), 20500–20518.
10. Kruszewski, J.; Krygowski, T. M. Definition of aromaticity basing on the harmonic oscillator model. *Tetrahedron Lett.* **1972**, *13* (36), 3839–3842.
11. Dobrowolski, J. C.; Ostrowski, S. The Form of the HOMA Geometric Aromaticity Index Is Universal and Allows the Design of Electronic and Magnetic Descriptors. *J. Org. Chem.* **2025**.
12. Arpa, E. M.; Durbeej, B. HOMER: a reparameterization of the harmonic oscillator model of aromaticity (HOMA) for excited states. *Phys. Chem. Chem. Phys.* **2023**, *25* (25), 16763–16771.
13. Bird, C. W. A new aromaticity index and its application to five-membered ring heterocycles. *Tetrahedron* **1985**, *41* (7), 1409–1414.
14. Yang, S.; Kertesz, M. Bond Length Alternation and Energy Band Gap of Polyyne. *J. Phys. Chem.* **2006**, *110*, 9771–9774.
15. Güell, M.; Matito, E.; Luis, J. M.; Poater, J.; Solà, M. Analysis of electron delocalization in aromatic systems: Individual molecular orbital contributions to Para-Delocalization Indexes (PDI). *J. Phys. Chem. A* **2006**, *110* (40), 11569–11574.
16. Poater, J.; Fradera, X.; Duran, M.; Solà, M. The delocalization index as an electronic aromaticity criterion: Application to a series of planar polycyclic aromatic hydrocarbons. *Chem. - A Eur. J.* **2003**, *9*, 400–406.
17. Matito, E.; Duran, M.; Solà, M. The aromatic fluctuation index (FLU): A new aromaticity index based on electron delocalization. *J. Chem. Phys.* **2005**, *122*.
18. Giambiagi, M.; De Giambiagi, M. S.; dos Santos Silva, C. D.; De Figueiredo, A. P. Multicenter bond indices as a measure of aromaticity. *Phys. Chem. Chem. Phys.* **2000**, *2*, 3381–3392.
19. Bultinck, P.; Ponec, R.; Van Damme, S. Multicenter bond indices as a new measure of aromaticity in polycyclic

aromatic hydrocarbons. *J. Phys. Org. Chem.* **2005**, *18*, 706–718.
20. Szczepanik, D. W.; Andrzejak, M.; Dyduch, K.; Żak, E.; Makowski, M.; Mazur, G.; Mrozek, J. A uniform approach to the description of multicenter bonding. *Phys. Chem. Chem. Phys.* **2014**, *16*, 20514–20523.
21. Becke, A. D.; Edgecombe, K. E. A simple measure of electron localization in atomic and molecular systems. *J. Chem. Phys.* **1990**, *92*, 5397–5403.
22. Schmider, H. L.; Becke, A. D. Chemical content of the kinetic energy density. *J. Mol. Struct. THEOCHEM* **2000**, *527*, 51–61.
23. Barbon, S. M.; Staroverov, V. N.; Gilroy, J. B. Effect of Extended π Conjugation on the Spectroscopic and Electrochemical Properties of Boron Difluoride Formazanate Complexes. *J. Org. Chem.* **2015**, *80*, 5226–5235.
24. Woller, T.; Geerlings, P.; De Proft, F.; Champagne, B. t.; Alonso, M. Aromaticity as a guiding concept for spectroscopic features and nonlinear optical properties of porphyrinoids. *Molecules* **2018**, *23*.
25. Setiawan, D.; Kraka, E.; Cremer, D. Quantitative Assessment of Aromaticity and Antiaromaticity Utilizing Vibrational Spectroscopy. *J. Org. Chem.* **2016**, *81*, 9669–9686.
26. Tran Ngoc, T.; Grabicki, N.; Irran, E.; Dumele, O.; Teichert, J. F. Photoswitching neutral homoaromatic hydrocarbons. *Nat. Chem.* **2023**, *15*, 377–385.
27. Demchuk, O. M.; Yoruk, B.; Blackburn, T.; Snieckus, V. A mixed naphthyl-phenyl phosphine ligand motif for Suzuki, Heck, and hydrodehalogenation reactions. *Synlett* **2006**, No. 18, 2908–2913.

Table S2. Crystal data and structure refinement for 1-5. X-ray structure of 4 was reported previously [27].

**Identification code**	**1**	**2**	**3**	**4** [27]	**5**
Empirical formula	$C_{33}H_{45}O_7P$	$C_{31}H_{39}O_4P$	$C_{32}H_{41}O_6P$	$C_{29}H_{35}O_4P$	$C_{32}H_{41}O_4P$
Formula weight	584.69	506.63	552.65	478.54	520.65
Temperature/K	293	293	293	180(2)	293
Crystal system	orthorhombic	orthorhombic	monoclinic	triclinic	triclinic
Space group	*Pbca*	$Pna2_1$	$P2_1/c$	*P*-1	*P*-1
a/Å	12.7176(2)	11.8424(1)	8.80010(17)	10.502(7)	8.7456(3)
b/Å	21.3579(5)	15.8657(1)	18.3104(3)	15.684(11)	9.3148(4)
c/Å	22.9453(4)	14.7789(1)	18.3194(3)	16.167(11)	19.0363(7)
α/°	90	90	90	86.975(13)	87.883(3)
β/°	90	90	90.2353(17)	86.020(12)	80.754(3)
γ/°	90	90	90	71.012(10)	71.354(4)
Volume/Å^3	6232.5(2)	2776.78(4)	2951.84(10)	2511(3)	1450.11(10)
Z	8	4	4	4	2
ϱ_{calc}g/cm^3	1.2462	1.2118	1.2435	1.266	1.1923
μ/mm^{-1}	1.156	1.139	1.166	0.143	1.103
F(000)	2522.2	1092.3	1188.8	1024.0	562.2
Crystal size/mm^3	0.2 × 0.2 × 0.1	0.3 × 0.1 × 0.1	0.25 × 0.1 × 0.08	0.40 × 0.20 × 0.10	0.3 × 0.2 × 0.05
Radiation	Cu Kα (λ = 1.54184)	Cu Kα (λ = 1.54184)	Cu Kα (λ = 1.54184)	MoKα (λ = 0.71073)	Cu Kα (λ = 1.54184)

2Θ range for data collection/°	7.7 to 152.84	8.18 to 136.68	6.82 to 152.6	3.68 to 56.78	9.42 to 137.04
Index ranges	-13 ≤ h ≤ 16, -20 ≤ k ≤ 26, -28 ≤ l ≤ 28	-14 ≤ h ≤ 14, -17 ≤ k ≤ 19, -17 ≤ l ≤ 17	-10 ≤ h ≤ 11, -23 ≤ k ≤ 21, -21 ≤ l ≤ 23	-13 ≤ h ≤ 13, -20 ≤ k ≤ 20, -21 ≤ l ≤ 17	-10 ≤ h ≤ 10, -11 ≤ k ≤ 11, -22 ≤ l ≤ 22
Reflections collected	42896	51823	20763	15951	38117
Independent reflections	6493 R_{int} = 0.0485, R_{sigma} = 0.0289	5076 R_{int} = 0.0275, R_{sigma} = 0.0134	6065 R_{int} = 0.0311, R_{sigma} = 0.0295	10799 R_{int} = 0.0986, R_{sigma} = 0.0531	5223 R_{int} = 0.1893, R_{sigma} = 0.0764
Data/restraints/parameters	6493/0/382	5076/0/327	6065/0/359	10799/0/629	5223/0/337
Goodness-of-fit on F^2	0.986	1.050	1.031	0.915	1.048
Final R indexes [I>=2σ (I)]	R_1 = 0.0503, wR_2 = 0.1384	R_1 = 0.0290, wR_2 = 0.0794	R_1 = 0.0488, wR_2 = 0.1297	R_1 = 0.1107, wR_2 = 0.2494	R_1 = 0.1572, wR_2 = 0.4269
Final R indexes [all data]	R_1 = 0.0672, wR_2 = 0.1499	R_1 = 0.0292, wR_2 = 0.0796	R_1 = 0.0639, wR_2 = 0.1421	R_1 = 0.2940, wR_2 = 0.3422	R_1 = 0.1712, wR_2 = 0.4394
Largest diff. peak/hole / e Å^{-3}	0.28/-0.29	0.15/-0.19	0.41/-0.51	1.01/-0.53	1.36/-0.72
Flack parameter	–	0.028(14)	–	–	–
CCDC No.	2482474	2482475	2482476	609270	2482477

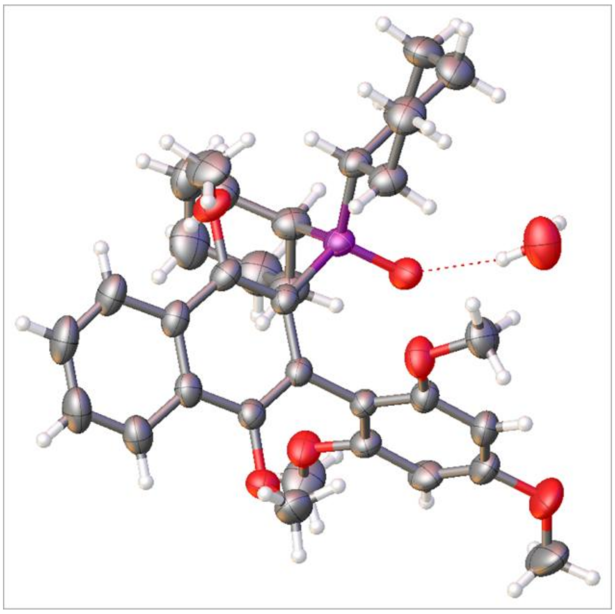

Figure S1. Molecular structure of 1. Ellipsoids with 50% probability.

Table S3. Bond lengths for 1.

Atom	Atom	Length/Å	Atom	Atom	Length/Å
P1	C21	1.823(2)	C21	C22	1.536(3)
P1	C1	1.844(2)	C21	C26	1.534(3)
P1	C27	1.818(2)	C17	C16	1.393(3)
P1	O6	1.4943(15)	C8	C7	1.419(3)
O3	C17	1.370(2)	O1	C11	1.439(3)
O3	C20	1.402(3)	C16	C15	1.382(3)
O2	C9	1.384(2)	C4	C5	1.361(4)
O2	C33	1.422(3)	C15	O5	1.380(3)
C2	C3	1.422(3)	C15	C14	1.376(3)
C2	C1	1.383(3)	O5	C19	1.371(4)
C2	O1	1.382(2)	C7	C6	1.365(3)
C10	C9	1.373(3)	C27	C28	1.533(3)
C10	C12	1.494(2)	C27	C32	1.505(3)
C10	C1	1.438(3)	C6	C5	1.390(4)
O4	C13	1.362(2)	C30	C31	1.539(4)
O4	C18	1.421(3)	C30	C29	1.490(4)
C13	C12	1.398(3)	C28	C29	1.506(4)
C13	C14	1.387(3)	C32	C31	1.535(4)
C9	C8	1.422(3)	C23	C24	1.515(4)
C12	C17	1.391(3)	C23	C22	1.519(3)
C3	C8	1.408(3)	C24	C25	1.514(4)
C3	C4	1.427(3)	C26	C25	1.538(3)

Table S4. Bond angles for 1.

Atom	Atom	Atom	Angle/°	Atom	Atom	Atom	Angle/°
C1	P1	C21	109.82(9)	C10	C1	C2	118.49(18)
C27	P1	C21	110.11(10)	C12	C17	O3	114.78(17)
C27	P1	C1	106.65(10)	C16	C17	O3	123.78(19)
O6	P1	C21	109.50(9)	C16	C17	C12	121.44(19)
O6	P1	C1	113.02(9)	C3	C8	C9	118.66(18)
O6	P1	C27	107.67(10)	C7	C8	C9	121.7(2)
C20	O3	C17	118.21(18)	C7	C8	C3	119.61(19)
C33	O2	C9	113.39(17)	C11	O1	C2	116.76(19)
C1	C2	C3	122.48(19)	C15	C16	C17	118.8(2)
O1	C2	C3	118.55(17)	C5	C4	C3	120.3(2)
O1	C2	C1	118.79(19)	O5	C15	C16	123.9(2)
C12	C10	C9	117.25(17)	C14	C15	C16	121.64(19)
C1	C10	C9	119.23(16)	C14	C15	O5	114.5(2)

C1	C10	C12	123.48(17)	C19	O5	C15	119.3(2)
C18	O4	C13	117.39(17)	C6	C7	C8	120.2(2)
C12	C13	O4	115.18(17)	C15	C14	C13	118.7(2)
C14	C13	O4	123.05(19)	C28	C27	P1	109.44(17)
C14	C13	C12	121.77(19)	C32	C27	P1	118.98(17)
C10	C9	O2	120.34(17)	C32	C27	C28	110.3(2)
C8	C9	O2	117.17(18)	C5	C6	C7	120.4(2)
C8	C9	C10	122.41(18)	C29	C30	C31	111.3(2)
C13	C12	C10	120.47(17)	C6	C5	C4	121.2(2)
C17	C12	C10	121.77(17)	C29	C28	C27	111.7(2)
C17	C12	C13	117.69(17)	C31	C32	C27	111.5(2)
C8	C3	C2	118.51(17)	C32	C31	C30	110.1(3)
C4	C3	C2	123.1(2)	C22	C23	C24	110.7(2)
C4	C3	C8	118.3(2)	C28	C29	C30	111.4(2)
C22	C21	P1	109.43(14)	C25	C24	C23	110.6(2)
C26	C21	P1	110.09(15)	C23	C22	C21	111.7(2)
C26	C21	C22	110.20(19)	C25	C26	C21	111.2(2)
C2	C1	P1	120.12(15)	C26	C25	C24	111.8(2)
C10	C1	P1	121.02(14)				

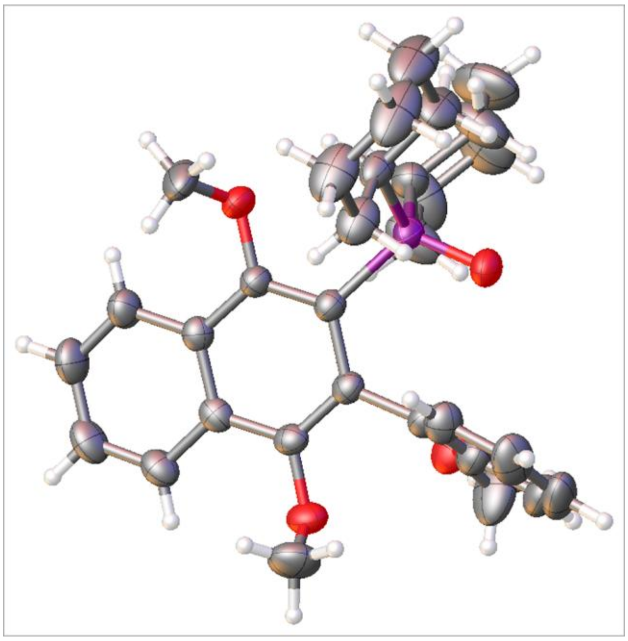

Figure S2. Molecular structure of 2. Ellipsoids with 50% probability.

Table S5. Bond lengths for 2.

Atom	Atom	Length/Å	Atom	Atom	Length/Å
P1	O1	1.4842(11)	C9	C10	1.376(2)

P1	C1	1.8422(15)	C10	C13	1.4998(19)
P1	C20	1.8306(15)	C13	C14	1.389(2)
P1	C26	1.8310(18)	C13	C18	1.390(2)
O2	C2	1.3846(17)	C14	C15	1.398(2)
O2	C11	1.445(2)	C15	C16	1.366(3)
O3	C9	1.3750(17)	C16	C17	1.377(3)
O3	C12	1.418(2)	C17	C18	1.391(2)
O4	C18	1.360(2)	C20	C21	1.533(3)
O4	C19	1.413(2)	C20	C25	1.527(3)
C1	C2	1.3781(19)	C21	C22	1.530(3)
C1	C10	1.4409(19)	C22	C23	1.518(5)
C2	C3	1.425(2)	C23	C24	1.511(4)
C3	C4	1.419(2)	C24	C25	1.531(3)
C3	C8	1.418(2)	C26	C27	1.531(3)
C4	C5	1.362(2)	C26	C31	1.527(3)
C5	C6	1.398(3)	C27	C28	1.509(4)
C6	C7	1.365(3)	C28	C29	1.499(4)
C7	C8	1.419(2)	C29	C30	1.497(4)
C8	C9	1.416(2)	C30	C31	1.527(3)

Table S6. Bond angles for 2.

Atom	Atom	Atom	Angle/°	Atom	Atom	Atom	Angle/°
C1	P1	O1	113.77(6)	C13	C10	C1	123.49(13)
C20	P1	O1	111.12(7)	C13	C10	C9	117.44(12)
C20	P1	C1	106.03(8)	C14	C13	C10	120.78(14)
C26	P1	O1	110.44(8)	C18	C13	C10	119.85(14)
C26	P1	C1	103.47(7)	C18	C13	C14	119.12(14)
C26	P1	C20	111.73(8)	C15	C14	C13	120.17(17)
C11	O2	C2	115.03(13)	C16	C15	C14	119.58(19)
C12	O3	C9	115.28(13)	C17	C16	C15	121.26(17)
C19	O4	C18	118.65(16)	C18	C17	C16	119.32(19)
C2	C1	P1	120.07(10)	C13	C18	O4	115.37(14)
C10	C1	P1	120.50(10)	C17	C18	O4	124.12(16)
C10	C1	C2	118.82(13)	C17	C18	C13	120.49(17)
C1	C2	O2	118.80(12)	C21	C20	P1	112.48(14)
C3	C2	O2	118.42(12)	C25	C20	P1	108.37(12)
C3	C2	C1	122.59(13)	C25	C20	C21	110.37(15)
C4	C3	C2	123.09(13)	C22	C21	C20	110.9(2)
C8	C3	C2	117.85(12)	C23	C22	C21	112.3(2)

C8	C3	C4	119.00(13)	C24	C23	C22	110.50(19)
C5	C4	C3	120.46(16)	C25	C24	C23	111.3(2)
C6	C5	C4	120.41(16)	C24	C25	C20	112.71(19)
C7	C6	C5	121.09(15)	C27	C26	P1	112.34(16)
C8	C7	C6	120.07(16)	C31	C26	P1	109.46(13)
C7	C8	C3	118.94(14)	C31	C26	C27	108.20(17)
C9	C8	C3	119.07(12)	C28	C27	C26	112.7(2)
C9	C8	C7	121.96(14)	C29	C28	C27	112.1(2)
C8	C9	O3	117.31(13)	C30	C29	C28	111.1(2)
C10	C9	O3	120.19(13)	C31	C30	C29	111.3(2)
C10	C9	C8	122.39(13)	C30	C31	C26	112.3(2)
C9	C10	C1	118.99(12)				

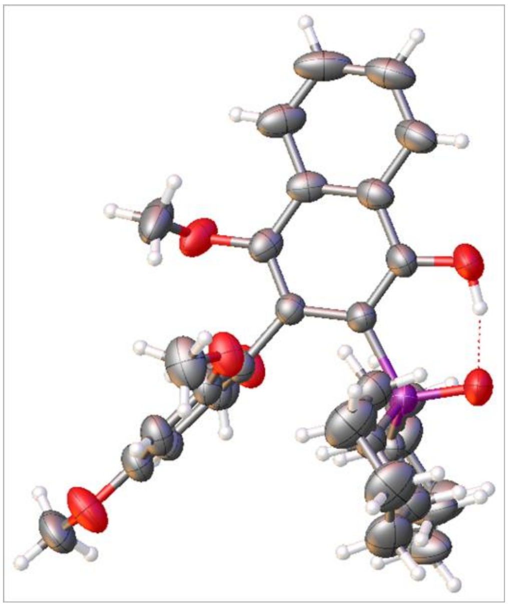

Figure S3. Molecular structure of 3. Ellipsoids with 50% probability.

Table S7. Bond lengths for 3.

Atom	Atom	Length/Å	Atom	Atom	Length/Å
P1	C1	1.8201(18)	C27	C28	1.545(3)
P1	C21	1.822(2)	C27	C32	1.538(3)
P1	O6	1.5114(14)	C17	C16	1.394(3)
P1	C27	1.818(2)	C3	C2	1.425(3)
C10	C12	1.501(2)	C3	C4	1.418(3)
C10	C1	1.441(2)	O1	C2	1.353(2)
C10	C9	1.371(2)	O4	C15	1.365(2)
O5	C17	1.363(2)	O4	C19	1.418(3)
O5	C20	1.425(3)	C16	C15	1.385(3)

O3	C13	1.369(2)	C15	C14	1.384(3)
O3	C18	1.428(3)	C4	C5	1.355(3)
C12	C13	1.398(3)	C28	C29	1.513(4)
C12	C17	1.401(3)	C5	C6	1.390(4)
C1	C2	1.389(2)	C22	C23	1.529(4)
C21	C22	1.536(3)	C7	C6	1.373(4)
C21	C26	1.536(3)	C26	C25	1.514(4)
O2	C9	1.385(2)	C32	C31	1.514(4)
O2	C11	1.425(3)	C30	C29	1.515(4)
C13	C14	1.387(3)	C30	C31	1.519(4)
C8	C3	1.405(3)	C25	C24	1.507(4)
C8	C9	1.421(3)	C23	C24	1.518(4)
C8	C7	1.419(3)			

Table S8. Bond angles for 3.

Atom	Atom	Atom	Angle/°	Atom	Atom	Atom	Angle/°
C21	P1	C1	108.46(9)	C16	C17	O5	122.61(17)
O6	P1	C1	109.00(8)	C16	C17	C12	122.02(18)
O6	P1	C21	108.93(10)	C2	C3	C8	119.22(17)
C27	P1	C1	107.62(9)	C4	C3	C8	119.66(19)
C27	P1	C21	113.35(9)	C4	C3	C2	121.1(2)
C27	P1	O6	109.39(10)	C19	O4	C15	118.0(2)
C1	C10	C12	123.84(15)	O2	C9	C10	119.20(17)
C9	C10	C12	117.10(15)	C8	C9	C10	122.45(17)
C9	C10	C1	119.02(16)	C8	C9	O2	118.35(17)
C20	O5	C17	118.23(16)	C15	C16	C17	118.56(18)
C18	O3	C13	118.27(17)	C3	C2	C1	121.32(18)
C13	C12	C10	120.84(16)	O1	C2	C1	124.30(18)
C17	C12	C10	121.30(16)	O1	C2	C3	114.38(17)
C17	C12	C13	116.94(16)	C16	C15	O4	123.9(2)
C10	C1	P1	124.33(13)	C14	C15	O4	114.8(2)
C2	C1	P1	116.48(14)	C14	C15	C16	121.29(18)
C2	C1	C10	119.17(16)	C5	C4	C3	120.4(2)
C22	C21	P1	108.61(15)	C15	C14	C13	118.96(19)
C26	C21	P1	111.88(16)	C29	C28	C27	111.2(3)
C26	C21	C22	109.26(18)	C6	C5	C4	120.2(2)
C11	O2	C9	113.82(16)	C23	C22	C21	112.0(2)
C12	C13	O3	115.54(16)	C6	C7	C8	119.9(2)
C14	C13	O3	122.43(18)	C25	C26	C21	112.2(2)
C14	C13	C12	122.01(18)	C7	C6	C5	121.3(2)

C9	C8	C3	118.69(16)	C31	C32	C27	112.7(2)
C7	C8	C3	118.5(2)	C31	C30	C29	111.2(2)
C7	C8	C9	122.8(2)	C24	C25	C26	112.7(3)
C28	C27	P1	111.36(18)	C24	C23	C22	111.4(3)
C32	C27	P1	109.04(15)	C23	C24	C25	110.8(2)
C32	C27	C28	108.68(17)	C30	C29	C28	112.2(2)
C12	C17	O5	115.36(16)	C30	C31	C32	111.6(3)

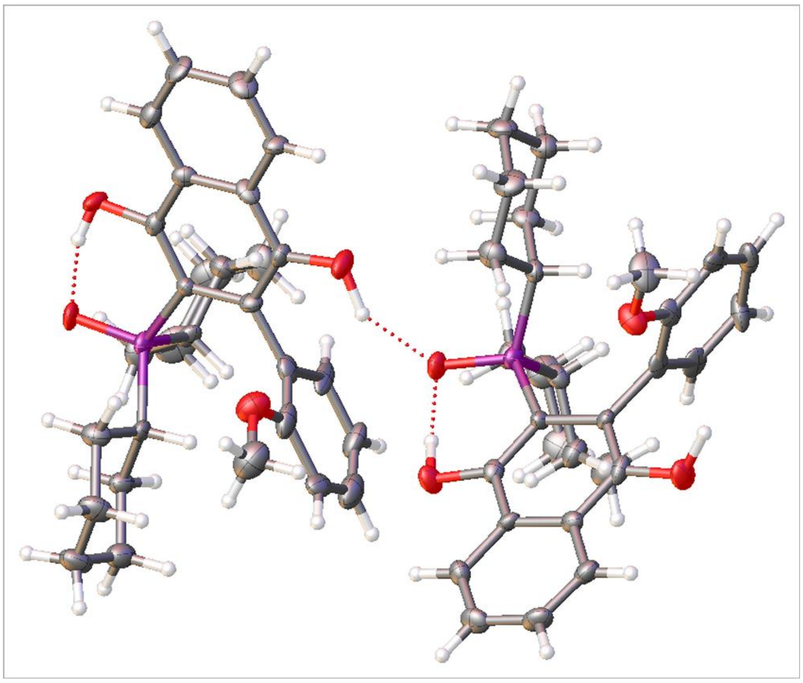

Figure S4. Molecular structure of 4. Ellipsoids with 50% probability.

Table S9. Bond lengths for 4.

Atom	Atom	Length/Å	Atom	Atom	Length/Å
P1	O4	1.520(5)	C19	C20	1.519(12)
P1	C24	1.797(8)	C20	C21	1.511(13)
P1	C8	1.813(7)	C21	C22	1.510(13)
P1	C18	1.825(8)	C22	C23	1.522(12)
P2	O8	1.514(5)	C24	C29	1.524(11)
P2	C53	1.817(8)	C24	C25	1.547(11)
P2	C47	1.824(8)	C25	C26	1.540(11)
P2	C37	1.824(8)	C26	C27	1.519(12)
O1	C6	1.366(8)	C30	C31	1.359(11)
O2	C9	1.369(8)	C30	C39	1.408(11)
O3	C12	1.339(11)	C31	C32	1.375(11)

O3	C17	1.424(11)	C32	C33	1.373(11)
O5	C35	1.388(8)	C33	C34	1.408(11)
O6	C38	1.369(8)	C34	C39	1.391(10)
O7	C41	1.352(10)	C34	C35	1.415(11)
O7	C46	1.409(10)	C35	C36	1.373(11)
C1	C2	1.379(11)	C36	C37	1.440(9)
C1	C10	1.404(11)	C36	C40	1.498(11)
C2	C3	1.429(11)	C37	C38	1.371(10)
C3	C4	1.354(11)	C38	C39	1.447(11)
C4	C5	1.421(10)	C40	C45	1.395(11)
C5	C10	1.400(10)	C40	C41	1.397(11)
C5	C6	1.425(11)	C41	C42	1.372(11)
C6	C7	1.383(11)	C42	C43	1.378(12)
C7	C8	1.454(10)	C43	C44	1.417(13)
C7	C11	1.495(11)	C44	C45	1.338(12)
C8	C9	1.380(10)	C47	C48	1.543(11)
C28	C27	1.495(13)	C47	C52	1.553(10)
C28	C29	1.528(11)	C48	C49	1.491(12)
C9	C10	1.427(10)	C49	C50	1.524(12)
C11	C12	1.359(13)	C50	C51	1.527(13)
C11	C16	1.426(12)	C51	C52	1.510(12)
C12	C13	1.423(12)	C53	C54	1.483(11)
C13	C14	1.388(13)	C53	C58	1.561(11)
C14	C15	1.331(14)	C54	C55	1.526(11)
C15	C16	1.361(12)	C55	C56	1.529(12)
C18	C19	1.490(11)	C56	C57	1.529(12)
C18	C23	1.555(11)	C57	C58	1.516(11)

Table S10. Bond angles for 4.

Atom	Atom	Atom	Angle/°	Atom	Atom	Atom	Angle/°
O4	P1	C24	110.4(3)	C29	C24	C25	112.6(6)
O4	P1	C8	107.6(3)	C29	C24	P1	115.1(6)
C24	P1	C8	112.0(4)	C25	C24	P1	108.9(5)
O4	P1	C18	108.3(3)	C26	C25	C24	110.4(7)
C24	P1	C18	110.8(4)	C27	C26	C25	112.2(7)
C8	P1	C18	107.6(4)	C28	C27	C26	112.0(7)
O8	P2	C53	110.7(3)	C24	C29	C28	110.6(7)
O8	P2	C47	108.9(3)	C31	C30	C39	120.6(7)
C53	P2	C47	109.2(4)	C30	C31	C32	120.6(8)

O8	P2	C37	107.9(3)	C33	C32	C31	120.3(8)
C53	P2	C37	110.6(4)	C32	C33	C34	120.2(7)
C47	P2	C37	109.4(4)	C39	C34	C33	119.1(7)
C12	O3	C17	117.0(8)	C39	C34	C35	118.9(7)
C41	O7	C46	118.2(7)	C33	C34	C35	122.0(7)
C2	C1	C10	120.6(7)	C36	C35	O5	123.2(7)
C1	C2	C3	119.5(8)	C36	C35	C34	122.3(7)
C4	C3	C2	119.9(8)	O5	C35	C34	114.5(7)
C3	C4	C5	121.2(8)	C35	C36	C37	119.4(7)
C10	C5	C4	119.0(7)	C35	C36	C40	118.4(6)
C10	C5	C6	119.7(7)	C37	C36	C40	122.2(6)
C4	C5	C6	121.2(7)	C38	C37	C36	119.1(7)
O1	C6	C7	124.8(7)	C38	C37	P2	116.4(5)
O1	C6	C5	114.3(7)	C36	C37	P2	124.5(6)
C7	C6	C5	120.9(7)	O6	C38	C37	125.7(7)
C6	C7	C8	119.7(7)	O6	C38	C39	113.2(7)
C6	C7	C11	118.5(7)	C37	C38	C39	121.1(7)
C8	C7	C11	121.8(7)	C34	C39	C30	119.0(7)
C9	C8	C7	118.8(7)	C34	C39	C38	119.2(7)
C9	C8	P1	117.5(5)	C30	C39	C38	121.8(7)
C7	C8	P1	123.7(6)	C45	C40	C41	118.2(8)
C27	C28	C29	112.7(8)	C45	C40	C36	120.6(8)
O2	C9	C8	125.0(7)	C41	C40	C36	121.1(8)
O2	C9	C10	113.4(7)	O7	C41	C42	125.7(8)
C8	C9	C10	121.6(7)	O7	C41	C40	114.2(7)
C5	C10	C1	119.8(7)	C42	C41	C40	120.2(8)
C5	C10	C9	119.2(7)	C41	C42	C43	120.2(9)
C1	C10	C9	121.0(7)	C42	C43	C44	120.0(8)
C12	C11	C16	119.4(9)	C45	C44	C43	118.5(9)
C12	C11	C7	121.5(8)	C44	C45	C40	122.6(9)
C16	C11	C7	119.0(8)	C48	C47	C52	108.0(6)
O3	C12	C11	115.5(9)	C48	C47	P2	112.1(6)
O3	C12	C13	124.2(9)	C52	C47	P2	109.6(6)
C11	C12	C13	120.3(9)	C49	C48	C47	112.2(8)
C14	C13	C12	117.2(9)	C48	C49	C50	111.9(8)
C15	C14	C13	123.0(10)	C49	C50	C51	109.7(8)
C14	C15	C16	120.5(10)	C52	C51	C50	112.5(8)
C15	C16	C11	119.6(10)	C51	C52	C47	111.9(7)
C19	C18	C23	110.5(7)	C54	C53	C58	112.0(7)
C19	C18	P1	112.6(6)	C54	C53	P2	115.0(5)

C23	C18	P1	109.5(5)	C58	C53	P2	108.6(5)
C18	C19	C20	111.8(7)	C53	C54	C55	111.4(7)
C21	C20	C19	110.7(7)	C54	C55	C56	111.4(7)
C22	C21	C20	110.9(8)	C55	C56	C57	110.9(7)
C21	C22	C23	112.3(8)	C58	C57	C56	111.7(7)
C22	C23	C18	110.5(7)	C57	C58	C53	110.8(7)

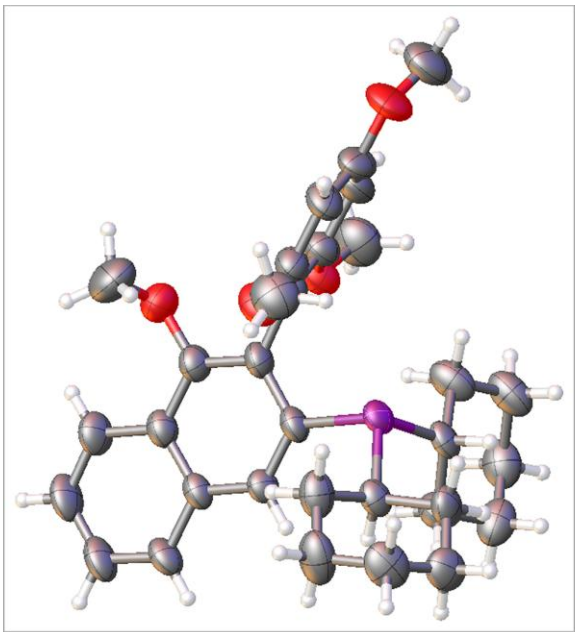

Figure S5. Molecular structure of 5. Ellipsoids with 50% probability.

Table S11. Bond lengths for 5.

Atom	Atom	Length/Å	Atom	Atom	Length/Å
P1	C1	1.858(7)	C9	C10	1.382(10)
P1	C21	1.853(8)	C10	C12	1.493(11)
P1	C27	1.867(8)	C12	C13	1.401(12)
O1	C13	1.354(11)	C12	C17	1.385(12)
O1	C18	1.408(12)	C13	C14	1.393(11)
O2	C15	1.371(10)	C14	C15	1.377(14)
O2	C19	1.405(15)	C15	C16	1.363(15)
O3	C17	1.370(11)	C16	C17	1.400(12)
O3	C20	1.413(13)	C21	C22	1.529(12)
O4	C9	1.376(10)	C21	C26	1.534(11)
O4	C11	1.426(14)	C22	C23	1.497(14)
C1	C2	1.352(10)	C23	C24	1.522(14)
C1	C10	1.439(12)	C24	C25	1.490(15)
C2	C3	1.407(10)	C25	C26	1.515(13)

C3	C4	1.413(11)	C27	C28	1.532(10)
C3	C8	1.418(12)	C27	C32	1.506(11)
C4	C5	1.386(11)	C28	C29	1.533(13)
C5	C6	1.383(14)	C29	C30	1.491(14)
C6	C7	1.355(13)	C30	C31	1.510(13)
C7	C8	1.431(10)	C31	C32	1.516(14)
C8	C9	1.420(11)			

Table S12. Bond angles for 5.

Atom	Atom	Atom	Angle/°	Atom	Atom	Atom	Angle/°
C21	P1	C1	102.6(3)	C17	C12	C13	117.4(7)
C27	P1	C1	101.5(3)	C12	C13	O1	116.1(7)
C27	P1	C21	102.8(4)	C14	C13	O1	122.3(8)
C18	O1	C13	119.7(8)	C14	C13	C12	121.5(9)
C19	O2	C15	118.2(9)	C15	C14	C13	118.9(9)
C20	O3	C17	118.8(8)	C14	C15	O2	115.3(9)
C11	O4	C9	112.6(8)	C16	C15	O2	123.5(10)
C2	C1	P1	123.5(6)	C16	C15	C14	121.2(8)
C10	C1	P1	117.6(5)	C17	C16	C15	119.6(10)
C10	C1	C2	118.8(7)	C12	C17	O3	115.5(7)
C3	C2	C1	123.3(7)	C16	C17	O3	123.2(9)
C4	C3	C2	122.1(8)	C16	C17	C12	121.3(9)
C8	C3	C2	118.8(7)	C22	C21	P1	111.1(6)
C8	C3	C4	119.1(7)	C26	C21	P1	118.2(5)
C5	C4	C3	120.0(9)	C26	C21	C22	109.8(7)
C6	C5	C4	120.4(8)	C23	C22	C21	113.1(9)
C7	C6	C5	121.6(8)	C24	C23	C22	112.1(8)
C8	C7	C6	119.9(9)	C25	C24	C23	110.3(8)
C7	C8	C3	118.9(7)	C26	C25	C24	112.9(8)
C9	C8	C3	118.0(7)	C25	C26	C21	111.8(7)
C9	C8	C7	123.1(8)	C28	C27	P1	108.7(5)
C8	C9	O4	117.6(7)	C32	C27	P1	111.4(6)
C10	C9	O4	120.4(7)	C32	C27	C28	110.4(7)
C10	C9	C8	122.0(8)	C29	C28	C27	111.0(7)
C9	C10	C1	119.1(7)	C30	C29	C28	112.5(8)
C12	C10	C1	121.4(6)	C31	C30	C29	110.2(8)
C12	C10	C9	119.5(7)	C32	C31	C30	111.6(8)
C13	C12	C10	120.3(8)	C31	C32	C27	111.6(8)
C17	C12	C10	122.3(8)				

Table S13. Intra- and intermolecular contacts in 1-5.

Crystal	D–H…A	D–H	D…A	H…A	D–H…A	Symmetry code	Molecular view
1	O7water–H7a…O6	0.87(4)	2.857(3)	2.00(4)	167(4)	–	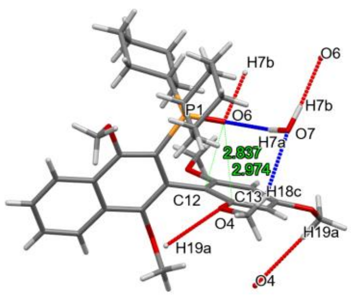
	O7water–H7b…O6	1.00(4)	2.982(3)	1.99(4)	175(4)	1-x,-y,1-z	
	H19a O4	0.96(2)	3.592(4)	2.63(2)	178(1)	-1/2+x,1/2-y,1-z	
	P1=O6…C12		2.837(2)		91.62(8)	intramolecular	
	P1=O6…C13		2.974(3)		106.82(8)	Intramolecular	
2	C5–H5…O1	0.930(3)	3.406(2)	2.633(2)	141.0(2)	1.5-x,-1/2+y,-1/2+z	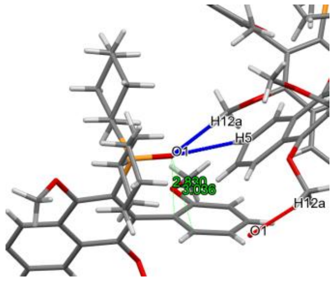
	C12–H12a…O1	0.96(1)	3.486(3)	2.55(1)	166.5(9)	1-x,1-y,-1/2+z	
	P1=O1…C13		2.830(2)		91.98(6)	intramolecular	
	P1=O1…C14		3.036(2)		102.54(6)	intramolecular	
3	O1–H1…O6	0.94(3)	2.487(2)	1.57(3)	164(3)	intramolecular	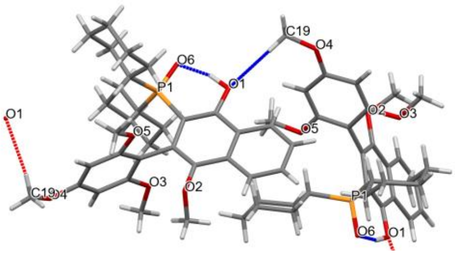
	C19–H19b…O1	0.96(1)	3.501(3)	2.55(1)	169(1)	-x,-1/2+y,1/2-z	
4	O6–H4…O8	1.1(1)	2.515(8)	1.5(1)	148(9)	intramolecular	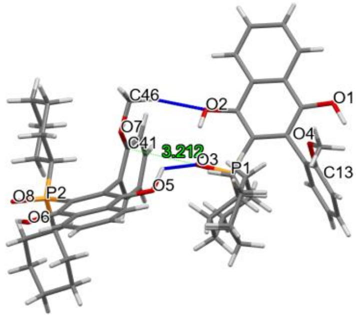
	O1–H1…O8	1.05(7)	2.696(7)	1.79(8)	142(7)	4a..4b	
	O2–H2…O3	0.84	2.528(7)	1.72(7)	160(8)	intramolecular	
	O5–H3…O3	1.1(1)	2.682(8)	1.9(1)	123(8)	x, y, 1+z	
	C18–H16…O6	0.98	3.50(1)	2.575	157.1	4a..4b	
	C46–H48…O2	0.98	3.45(1)	2.522	158.0	4a..4b	
	P1=O3…C41		3.212(9)			4a…4b	
	P2=O8…C13		3.27(1)			4a…4b	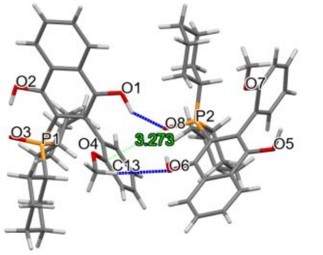
5	P1…C12		3.103(9)			intramolecular	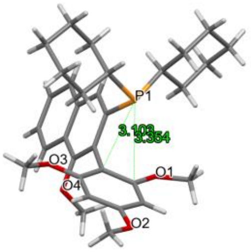
	P1…C12		3.354(8)			intramolecular	

Table S14. Hirshfeld Surface analysis results for 1-5: 2D fingerprint plots, dnorm surfaces, shape index and curvedness.

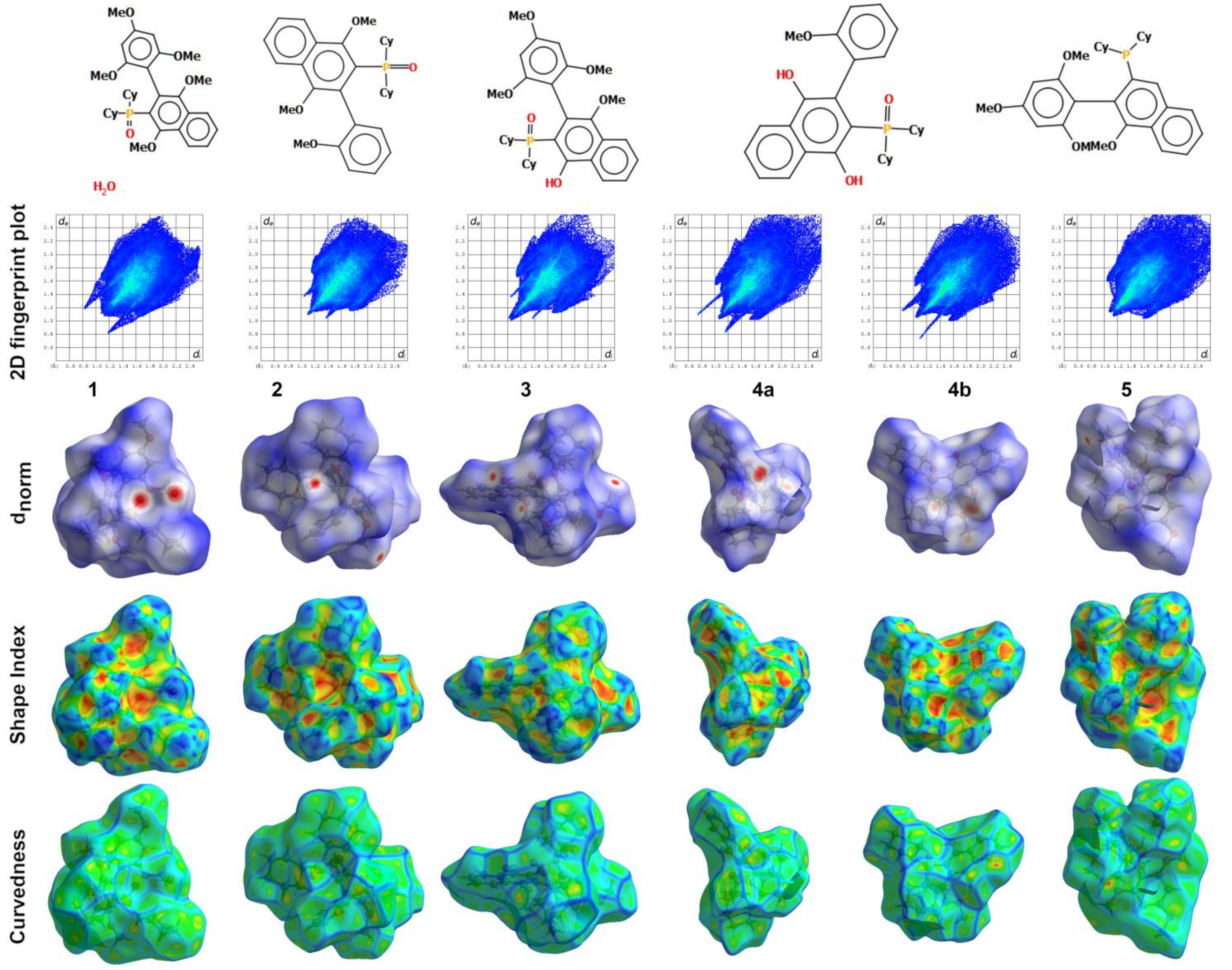

Table S15. Contributions to Hirshfeld surface area for various contacts in 1-5.

Contribution	1	2	3	4a	4b	5
O…H	13	8.1	13.5	10.8	10.8	9
C…H	12.5	13.7	14.1	19.3	17.6	16.2
H…H	74.5	78.2	72.3	69.1	70.8	74.1
C…O				0.6	0.6	
O…O			0.1	0.1	0.1	
C…C				0.1	0.1	
P...H						0.7

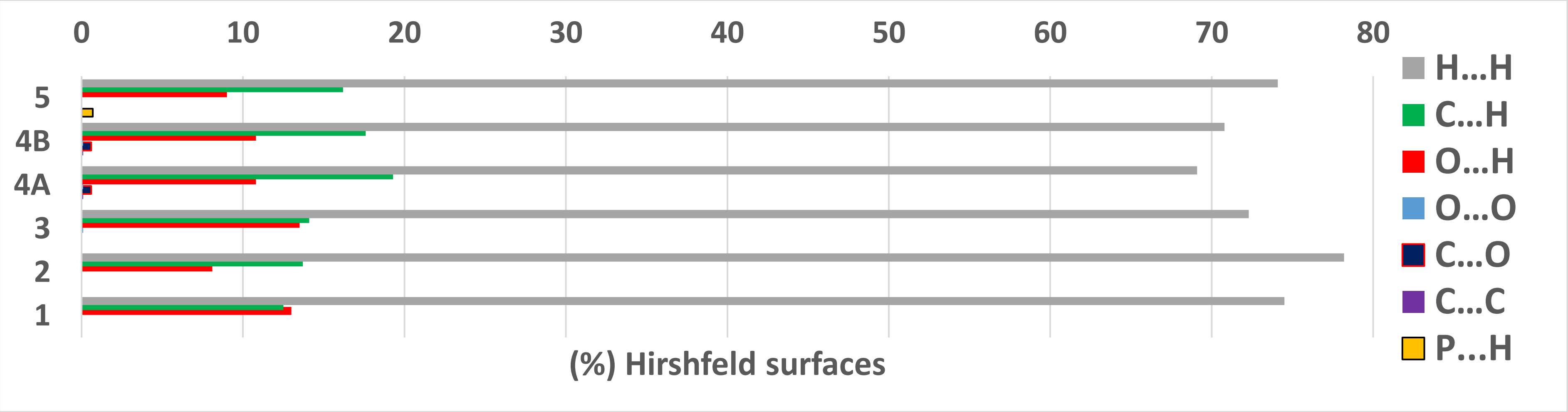

Table S16. Geometric aromaticity indices HOMA and HOMER calculated for X-ray (XRD) and optimized (DFT) structures and magnetic indices NICS(0), NICS(1), and NICS(1)zz.

	1	2	3	4a	4b	5
**Ph HOMA XRD**	0.986	0.968	0.986	0.674	0.831	0.953
**N1 HOMA XRD**	0.762	0.735	0.750	0.674	0.690	0.728
**N2 HOMA XRD**	0.830	0.821	0.847	0.817	0.913	0.809
**Ph HOMA DFT**	0.962	0.968	0.946	0.936	–	0.957
**N1 HOMA DFT**	0.697	0.716	0.631	0.630	–	0.741
**N2 HOMA DFT**	0.808	0.798	0.843	0.850	–	0.803
**Ph HOMER XRD**	-1.343	-1.651	-0.992	-3.090	-2.391	-1.584
**N1 HOMER XRD**	-0.308	-0.324	-0.278	-0.308	-0.756	-0.896
**N2 HOMER XRD**	-1.247	-1.101	-1.326	-1.083	-1.814	-1.075
**Ph HOMER DFT**	-0.372	-0.508	-0.222	-0.305	–	-0.323
**N1 HOMER DFT**	0.237	0.231	0.373	0.336	–	0.147
**N2 HOMER DFT**	-0.304	-0.306	-0.298	-0.301	–	-0.292
**Ph NICS(0) XRD**	-8.887	-7.456	-8.332	-7.525	-7.172	-8.501
**N1 NICS(0) XRD**	-9.575	-9.883	-8.084	-6.982	-7.941	-7.969
**N2 NICS(0) XRD**	-7.672	-7.724	-7.550	-7.652	-7.575	-7.541
**Ph NICS(0) DFT**	-9.289	-8.361	-8.855	-7.627	–	-8.975
**N1 NICS(0) DFT**	-9.928	-10.219	-8.126	-7.933	–	-9.041
**N2 NICS(0) DFT**	-8.360	-8.452	-8.337	-8.335	–	-8.244
**Ph NICS(1) XRD**	-7.508	-8.672	-7.381	-9.358	-8.875	-7.044
**N1 NICS(1) XRD**	-9.117	-9.389	-7.829	-7.498	-7.922	-9.684
**N2 NICS(1) XRD**	-9.385	-9.49	-9.802	-10.222	-9.913	-9.975
**Ph NICS(1) DFT**	-7.596	-8.875	-7.642	-8.430	–	-7.476
**N1 NICS(1) DFT**	-9.388	-9.553	-8.141	-7.652	–	-9.686
**N2 NICS(1) DFT**	-9.215	-9.282	-9.602	-9.868	–	-9.817
**Ph NICS(1)zz XRD**	-20.023	-26.048	-19.661	-29.257	-28.364	-19.188
**N1 NICS(1)zz XRD**	-22.345	-23.080	-19.444	-18.189	-19.215	-24.729
**N2 NICS(1)zz XRD**	-25.236	-25.631	-26.682	-27.915	-26.796	-26.858
**Ph NICS(1)zz DFT**	-20.149	-26.124	-20.208	-25.513	–	-19.622
**N1 NICS(1)zz DFT**	-23.024	-23.596	-18.908	-18.962	–	-24.776
**N2 NICS(1)zz DFT**	-24.369	-24.636	-25.510	-26.602	–	-26.214

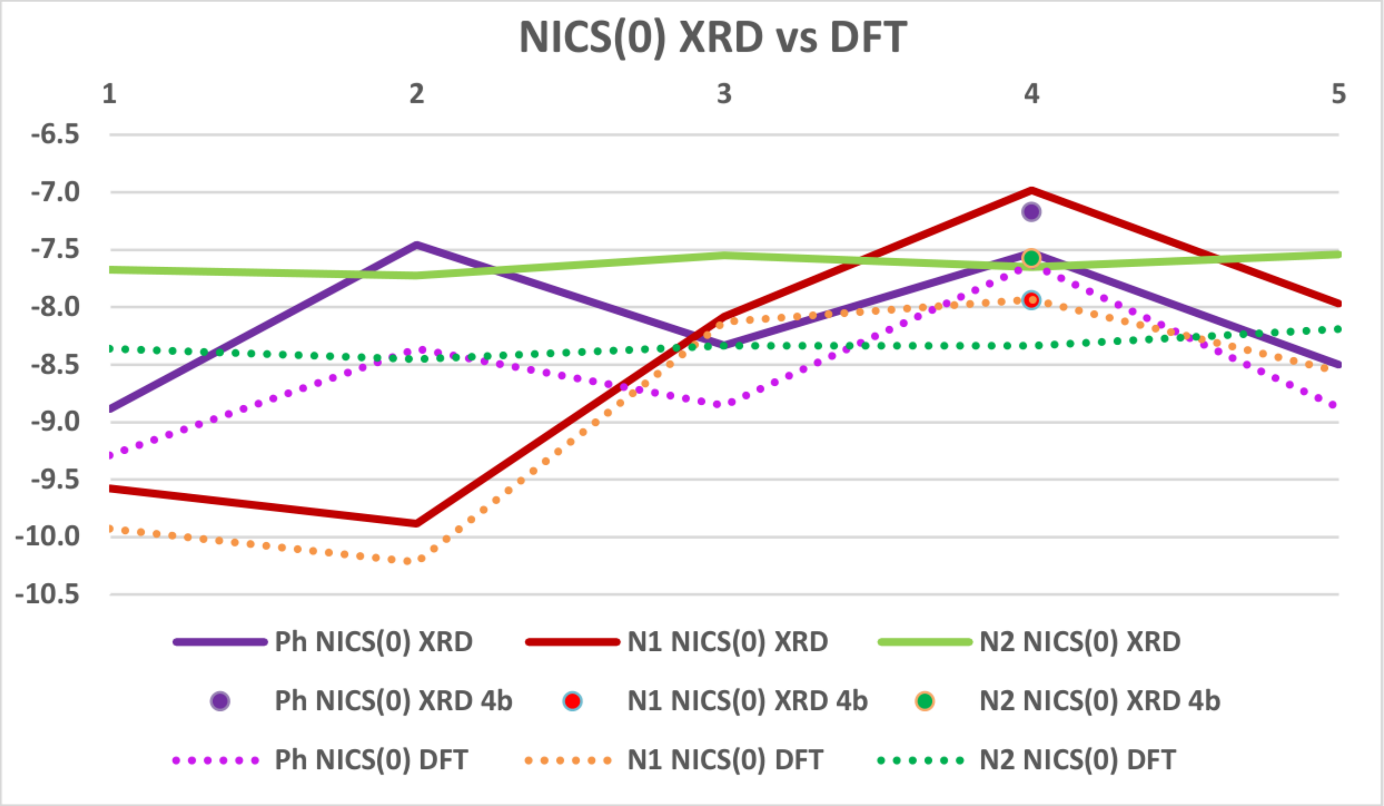

Figure S6. Aromaticity index NICS(0) calculated for X-ray (XRD) and optimized (DFT) geometries for compounds 1-5. In crystal 4 the data for the second symmetrically independent molecule are marked as circles and denoted 4b. Ph, N1 and N2 denote aromatic rings.

Table S17. Visualization of aromaticity indices HOMA and HOMER calculated for X-ray (XRD) and optimized (DFT) geometry.

Lig-and	HOMA	HOMER
L1 XRD	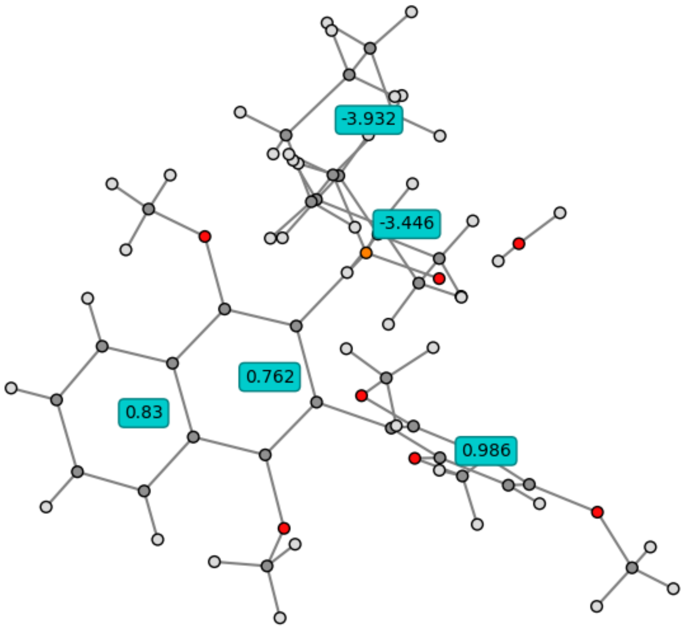	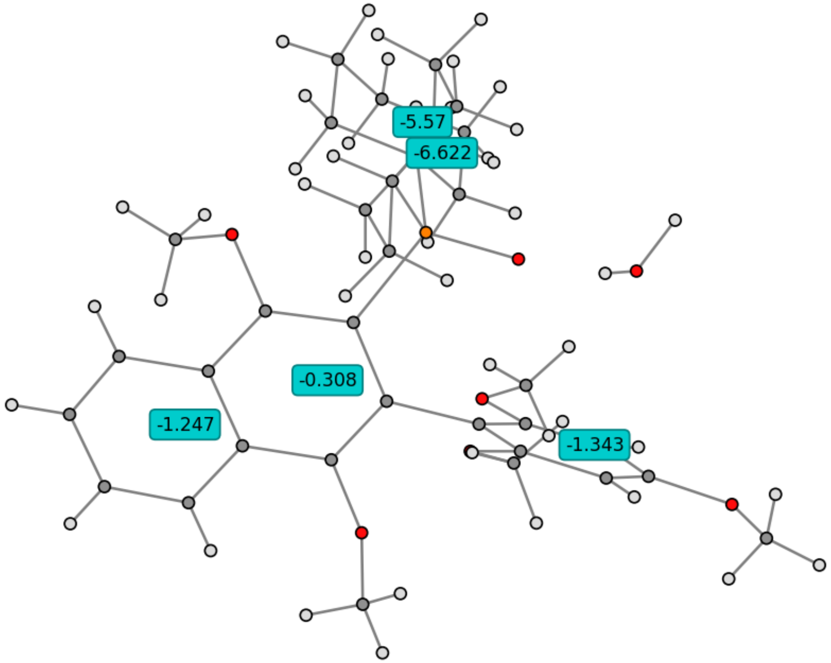

**L2**<br>**XRD**	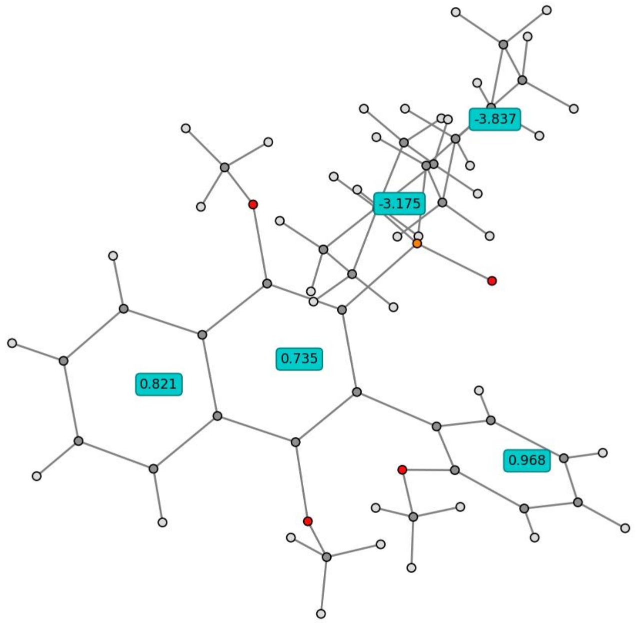	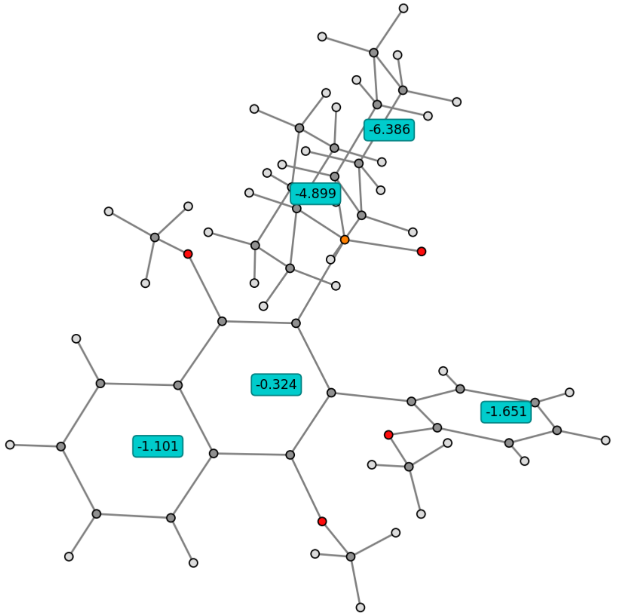
**L3**<br>**XRD**	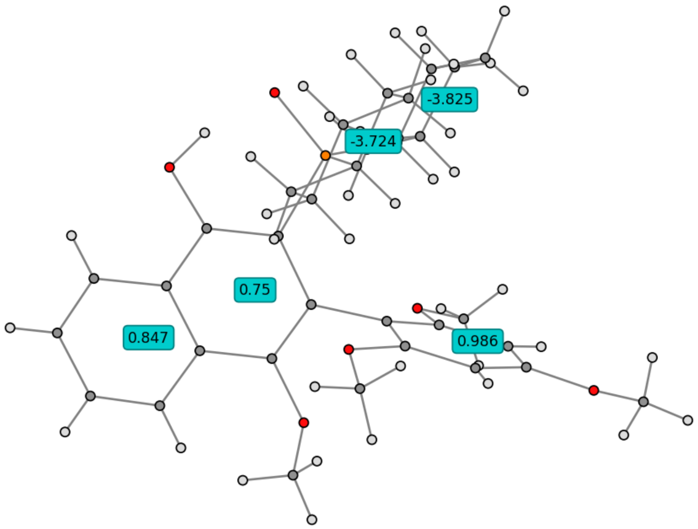	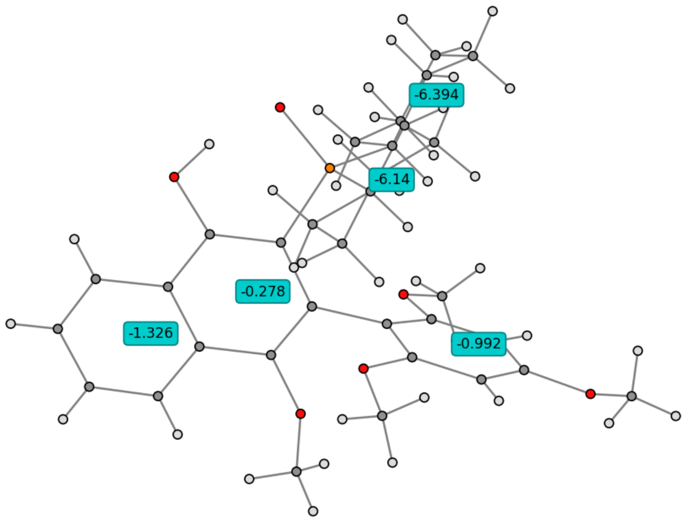

**L4<br>XRD**	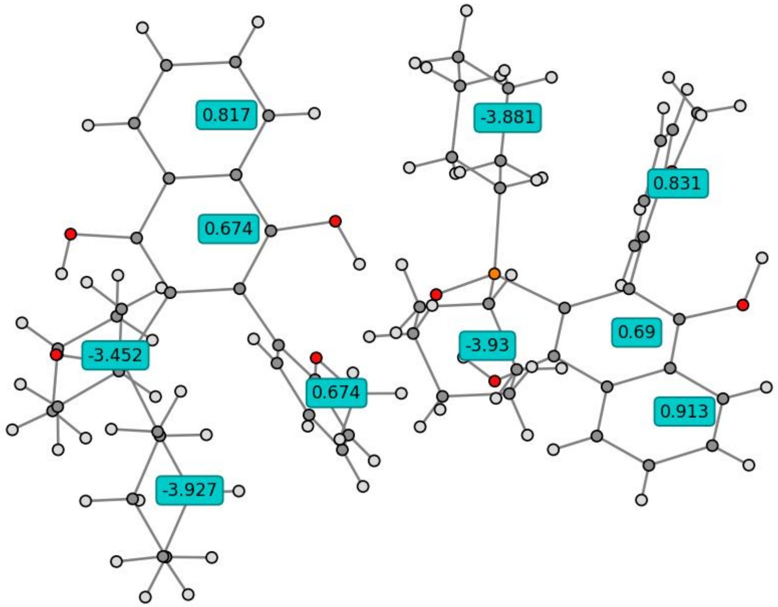	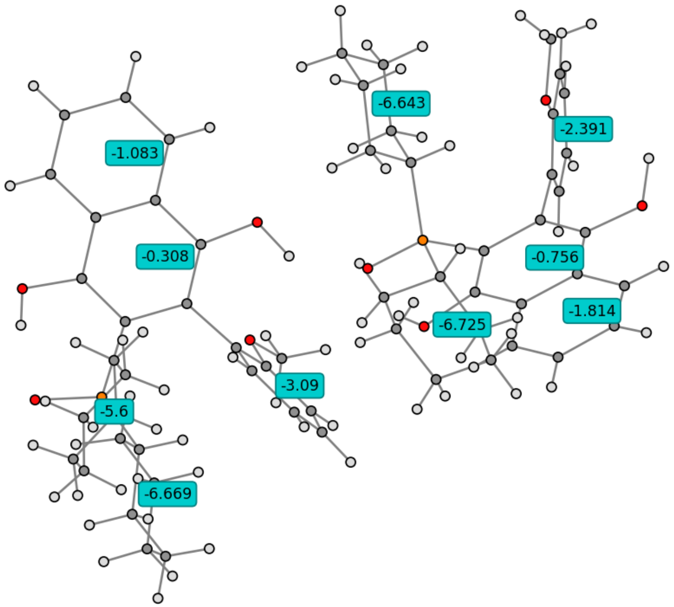
**L5<br>XRD**	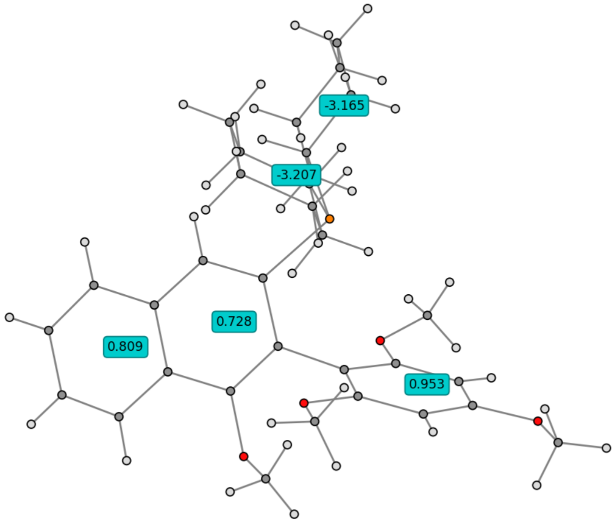	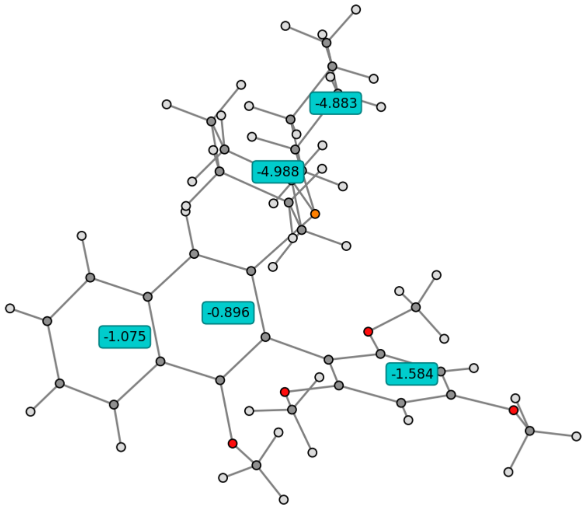

<table>
<tr>
<td>L1
DFT</td>
<td>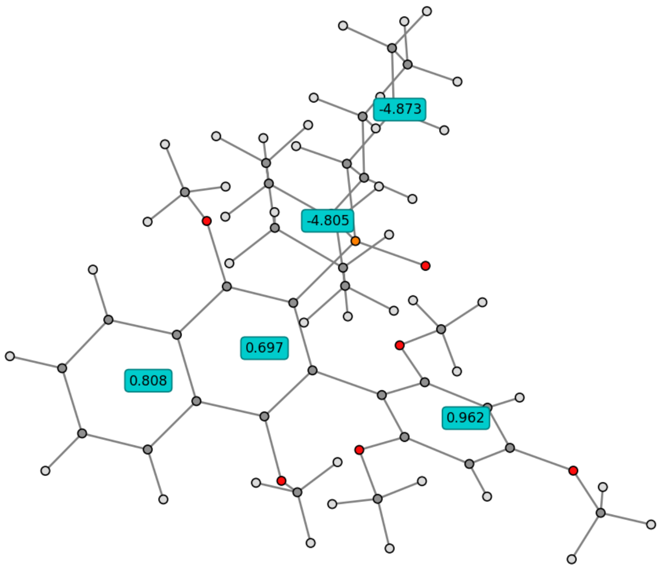
</td>
<td>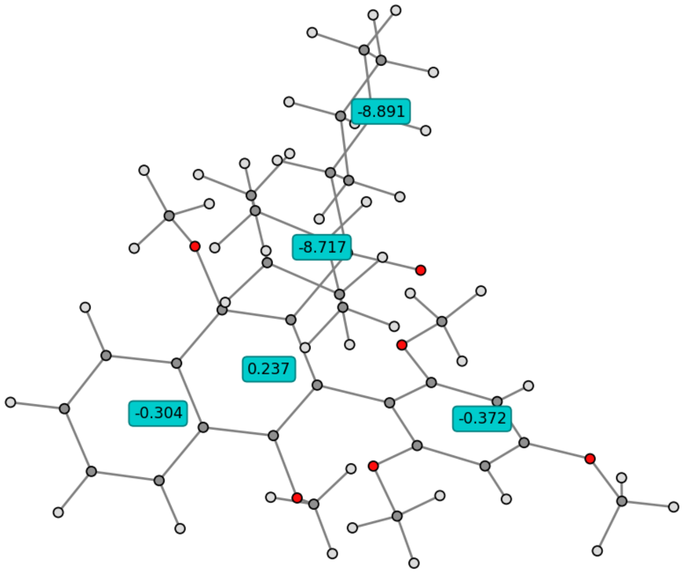
</td>
</tr>
<tr>
<td>L2
DFT</td>
<td>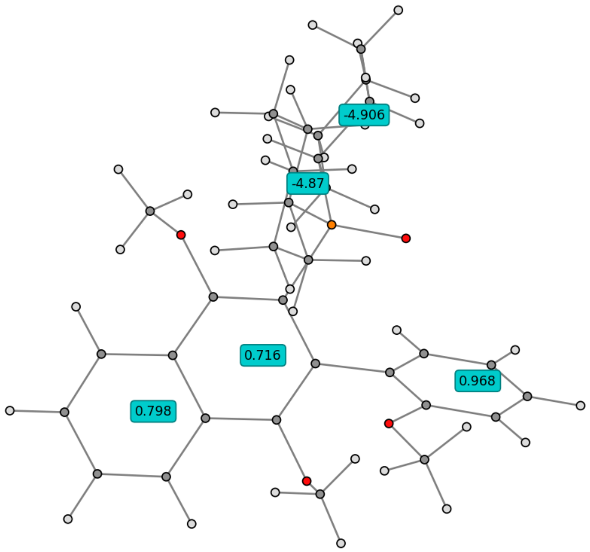
</td>
<td>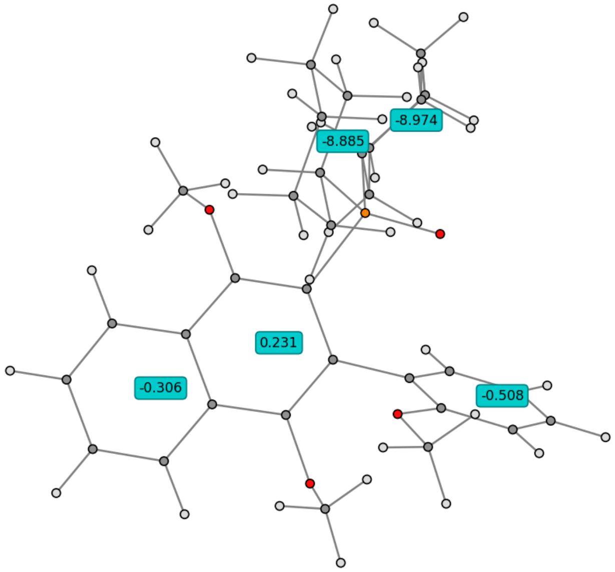
</td>
</tr>
</table>

<table>
<tr><td>L3
DFT</td><td>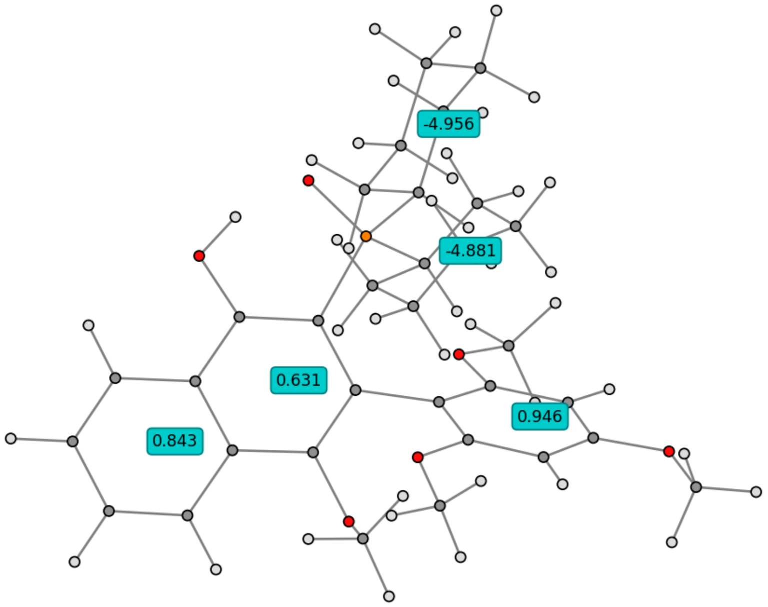
</td><td>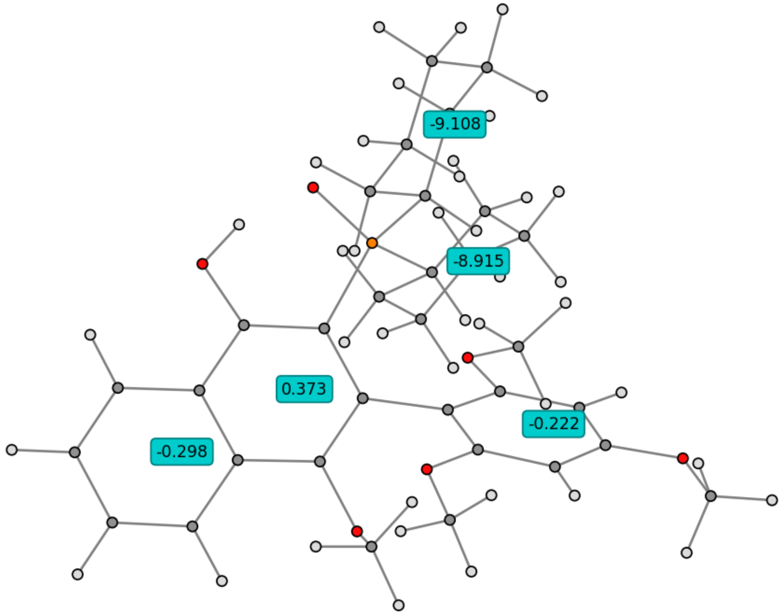
</td></tr>
</table>

**L4 DFT**	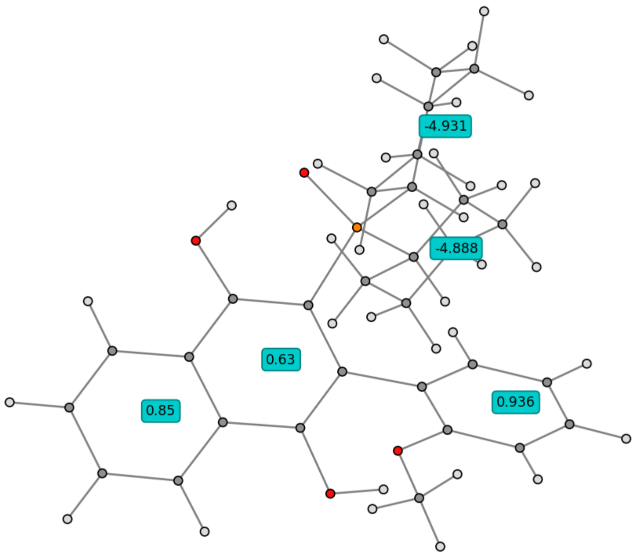-4.931<br>-4.888<br>0.63<br>0.85<br>0.936	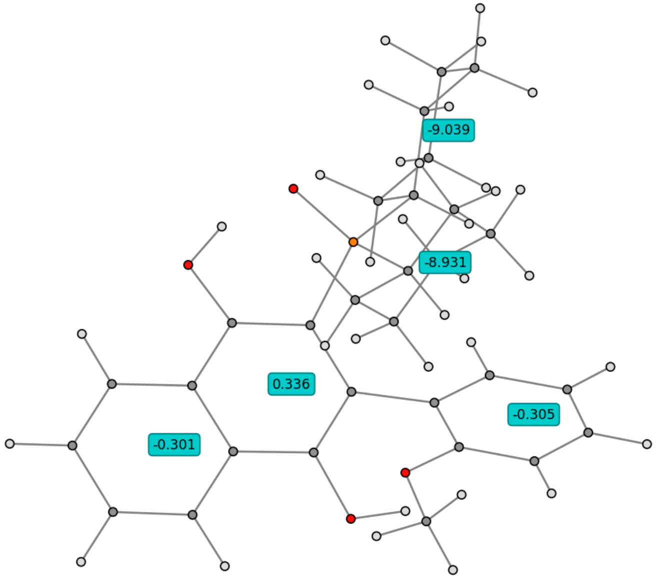-9.039<br>-8.931<br>0.336<br>-0.301<br>-0.305
**L5 DFT**	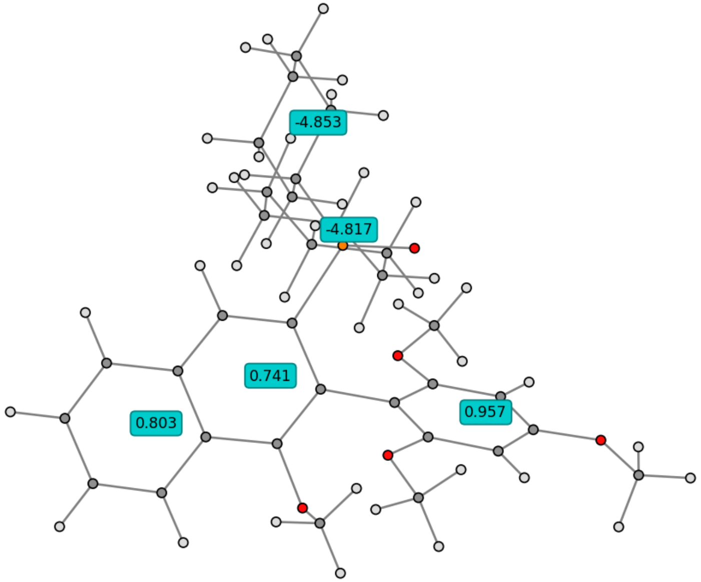-4.853<br>-4.817<br>0.741<br>0.803<br>0.957	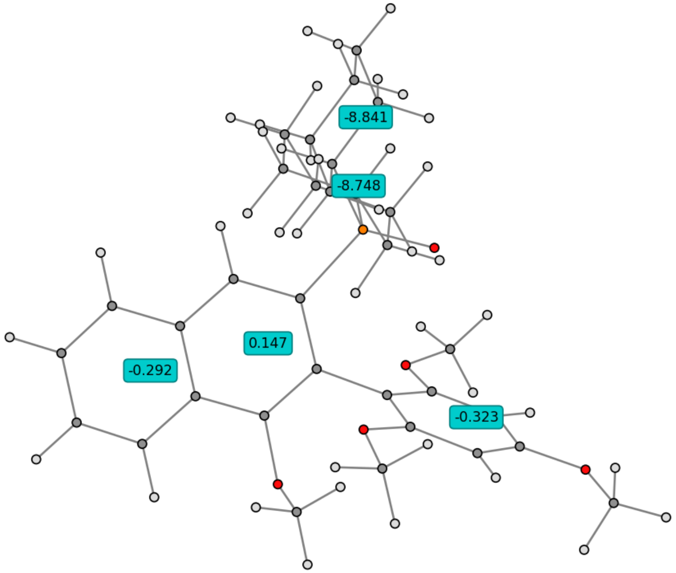-8.841<br>-8.748<br>0.147<br>-0.292<br>-0.323

Cell line maintenance and *in vitro* experiments

The cytotoxicity of selected biaryl monophosphines was evaluated *in vitro* towards normal VERO (ATCC, CCL-81) cells and cancer-derived cell line H1HeLa (cervical adenocarcinoma; ATCC (American Type Culture Collection) CRL-1958) using 3-(4,5-dimethylthiazol-2-yl)-2,5-diphenyltetrazolium bromide (MTT) based protocol.

Media used for *in vitro* culturing included Dulbecco's Modified Eagle's Medium (DMEM, Corning, Tewksbury, MA, USA) used for VERO cells and Modified Eagle Medium (MEM, Corning) used for H1HeLa. Cell media used in the experiments were supplemented with antibiotics (Penicillin-Streptomycin Solution, Corning) and fetal bovine serum (FBS, Corning) – 10% (cell passaging) and 2% (cell maintenance and experiments). Phosphate-buffered saline (PBS) and trypsin were bought from Corning, whereas MTT (3-(4,5-dimethylthiazol-2-yl)-2,5-diphenyltetrazolium bromide) from Sigma (Sigma-Aldrich, St. Louis, MO, USA). SDS was purchased from PanReac Applichem (Darmstadt, Germany). Incubation was carried out in a 5% CO_2 atmosphere at 37°C (CO_2 incubator, Panasonic Healthcare Co., Tokyo, Japan).

Concentrations of cells for passaging were as follows: VERO – $1.5*10^5$ cells/mL, H1HeLa – $2*10^5$ cells/mL. Routine observations of cell cultures and experiments were carried out using an inverted microscope (CKX41, Olympus Corporation, Tokyo, Japan) equipped with a camera (Moticam 3+, Motic, Hong Kong), and the results were recorded (Motic Images Plus 2.0, Motic).

Stock solutions of biaryl monophosphines were prepared by dissolving the samples in cell culture grade DMSO (PanReac Applichem).

Evaluation of cytotoxicity

Cytotoxicity was tested using an MTT-based protocol. The cells were passaged into 96-well plates (Falcon, TC-treated, Corning) and, after overnight incubation, treated with serial dilutions of extract or fraction stock solutions for 24 h. Simultaneously, the cytotoxicity of DMSO in concentrations equal to those present in the dilutions of stock solutions was tested to exclude any effect on the assay. Afterwards, the media was removed, cells were washed with PBS, and 10% of MTT solution (5 mg/mL) in cell media was added, and the incubation continued for the next 4 h. Subsequently,

the SDS/DMF/PBS (14% SDS, 36% DMF, 50% PBS) solvent was used (100 μL per well) to dissolve the precipitated formazan crystals, and the plates were left at 37°C overnight. Finally, the Synergy H1 Multi-Mode Microplate Reader (BioTek Instruments, Inc., Winooski, Vermont, USA) with Gen5 software (ver. 3.09.07; BioTek Instruments, Inc.) was used to measure the absorbance (540 and 620 nm). The resulting data were analyzed in GraphPad Prism (version 10.2) to determine the 50% cytotoxic concentration (CC_{50}) from dose-response curves.

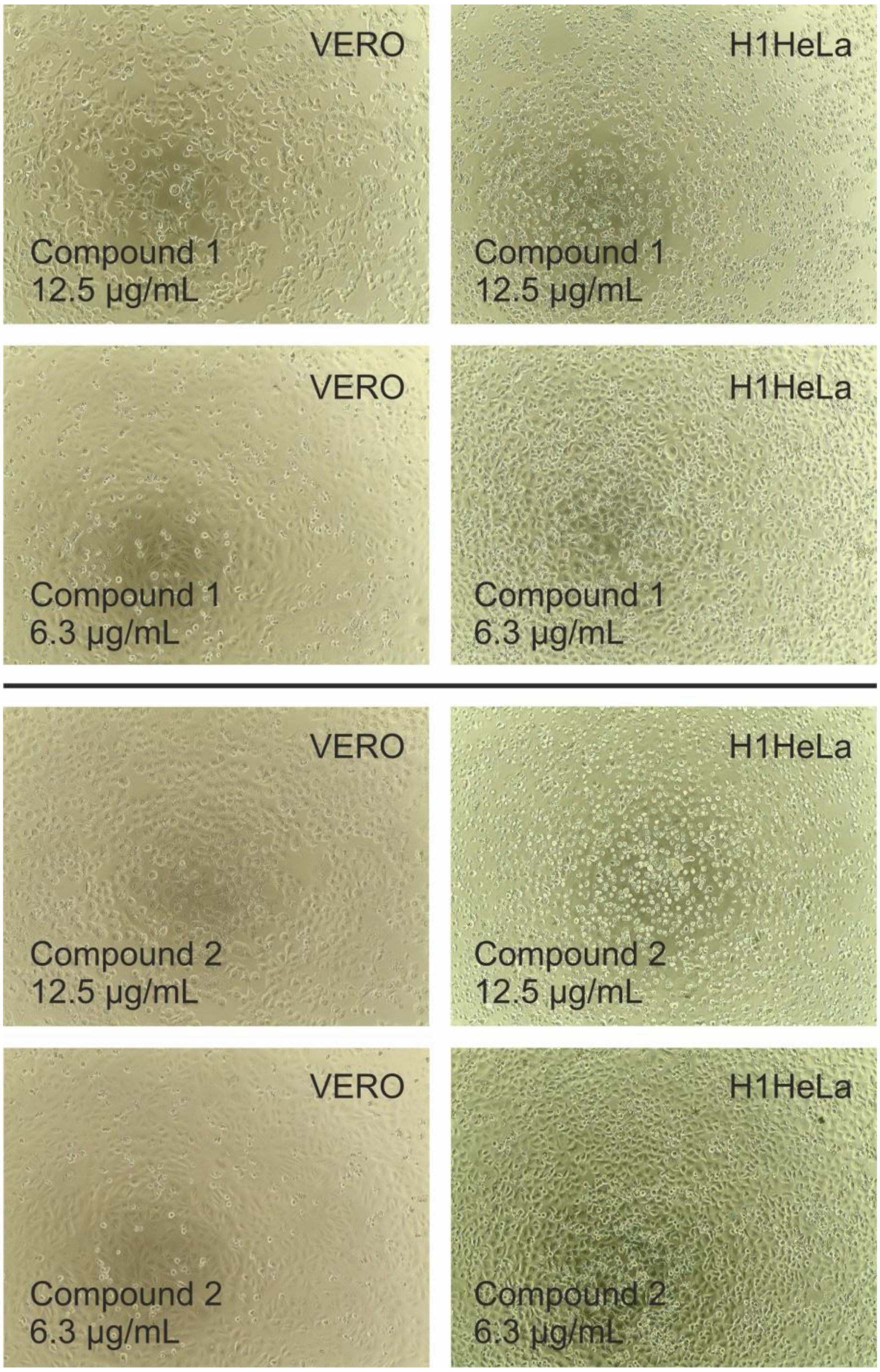

Figure S7. The influence of compounds 1 and 2 on the morphology of VERO and H1HeLa cellular monolayer. (VERO – normal kidney cells; H1HeLa - cervical adenocarcinoma).

Figure S8. The ^{1}H NMR spectrum for compound **1**.

24.09.2025 16:29:03
5 mm PABBO BB-1H/D Z-GRD Z119470/0011

Acquisition Time (sec)	3.1719	*Comment*	5 mm PABBO BB-1H/D Z-GRD Z119470/0011			*Date*	11 Oct 2015 14:24:32
Date Stamp	11 Oct 2015 14:24:32						
File Name	\\MYCLOUDEX2ULTRA\DATA\SPECTRA ARCHIVE\NMR\LIGANDY\MEO-SYMPHOS\MEO-SYMPHOSO 10C\3\FID						
Frequency (MHz)	500.13	*Nucleus*	1H	*Number of Transients*	16	*Origin*	spect
Original Points Count	32768	*Owner*	nmr	*Points Count*	32768	*Pulse Sequence*	zg30
Receiver Gain	161.00	*SW(cyclical) (Hz)*	10330.58	*Solvent*	CHLOROFORM-d	*Spectrum Offset (Hz)*	3080.6296
Spectrum Type	STANDARD	*Sweep Width (Hz)*	10330.26	*Temperature (degree C)*	10.044		

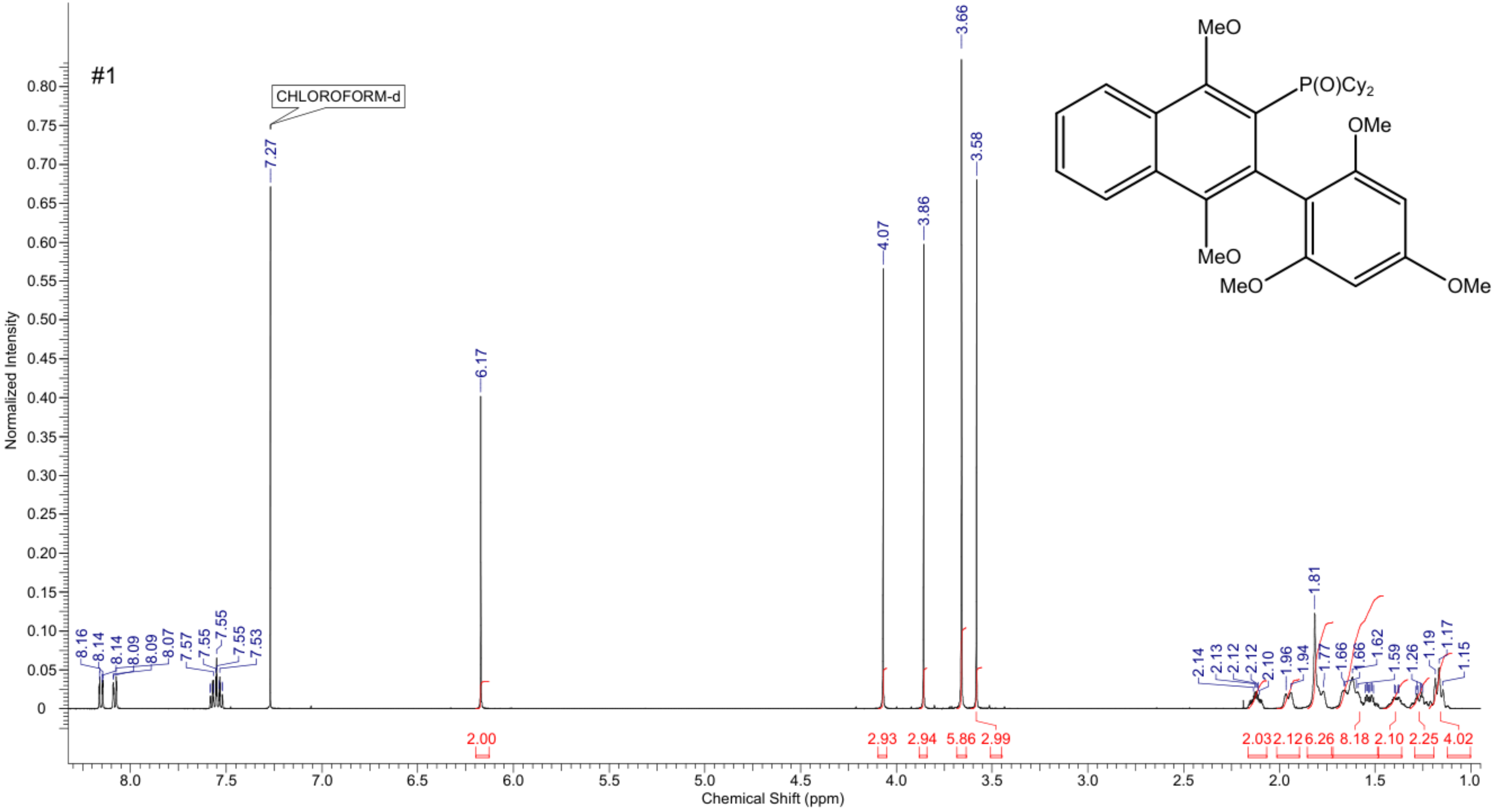

Figure S9. The ^{13}C NMR spectrum for compound **1**.

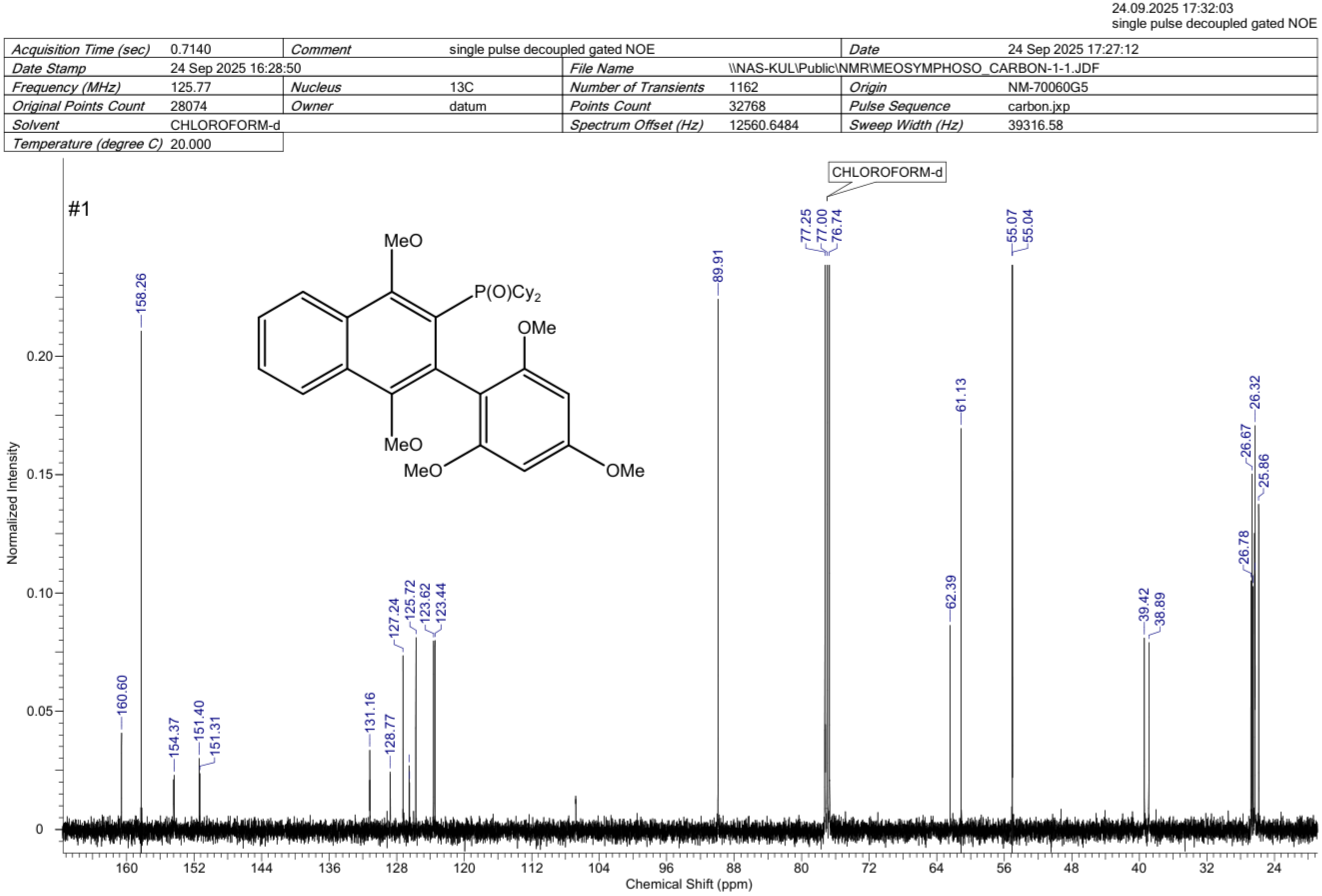

Figure S10. The ³¹P NMR spectrum for compound **1**.

24.09.2025 16:29:53
5 mm PABBO BB-1H/D Z-GRD Z119470/0011

Acquisition Time (sec)	0.4020	*Comment*	5 mm PABBO BB-1H/D Z-GRD Z119470/0011			*Date*	11 Oct 2015 14:22:24
Date Stamp	11 Oct 2015 14:22:24						
File Name	\\MYCLOUDEX2ULTRA\DATA\SPECTRA ARCHIVE\NMR\LIGANDY\MEO-SYMPHOS\MEO-SYMPHOSO 10C\2\FID					*Frequency (MHz)*	202.45
Nucleus	31P	*Number of Transients*	16	*Origin*	spect	*Original Points Count*	32768
Owner	nmr	*Points Count*	32768	*Pulse Sequence*	zgpg30	*Receiver Gain*	2050.00
SW(cyclical) (Hz)	81521.73	*Solvent*	CHLOROFORM-d	*Spectrum Offset (Hz)*	-10122.8525	*Spectrum Type*	STANDARD
Sweep Width (Hz)	81519.25	*Temperature (degree C)*	10.503				

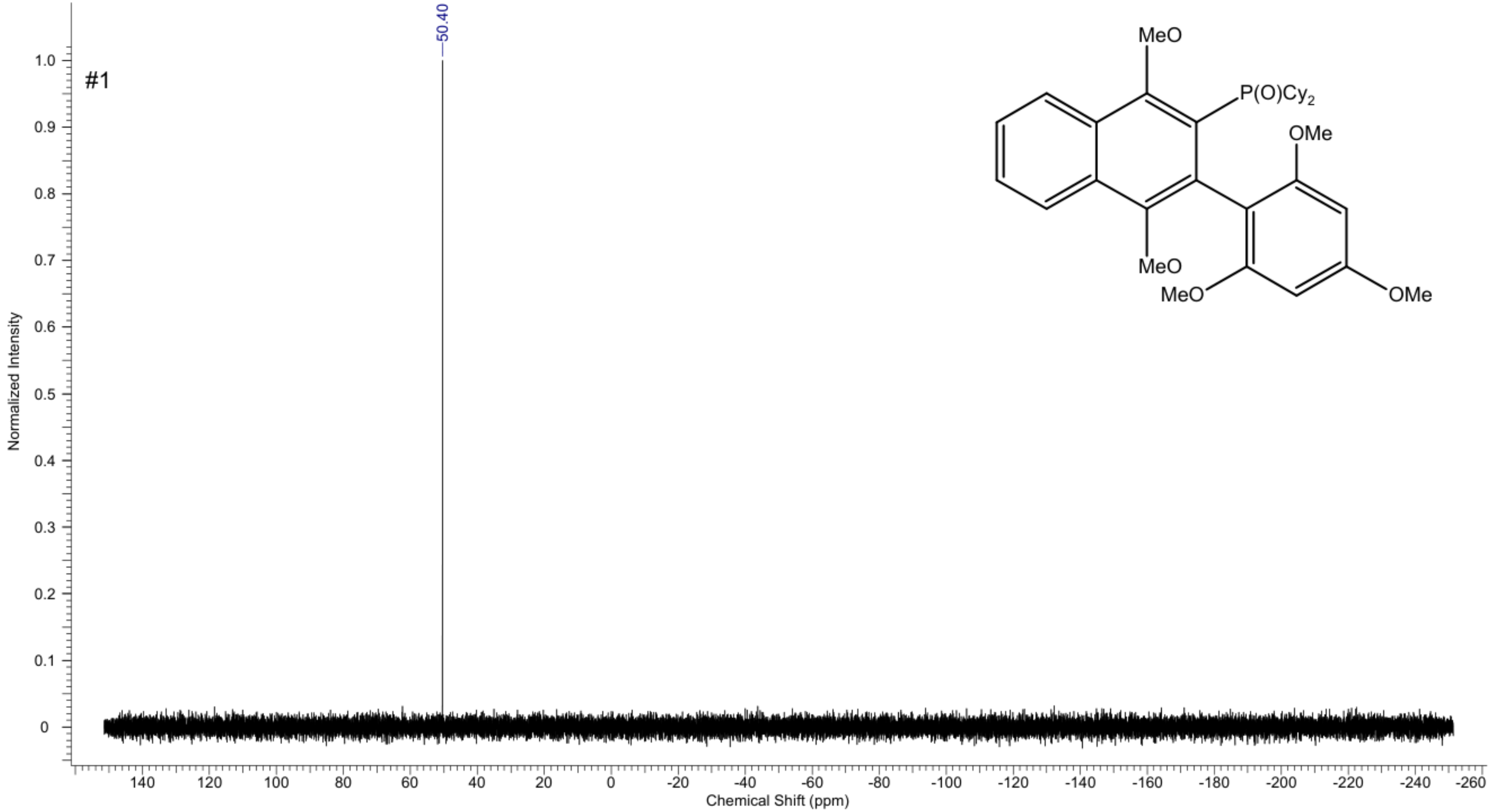

Figure S11. The ^{1}H NMR spectrum for compound **2**.

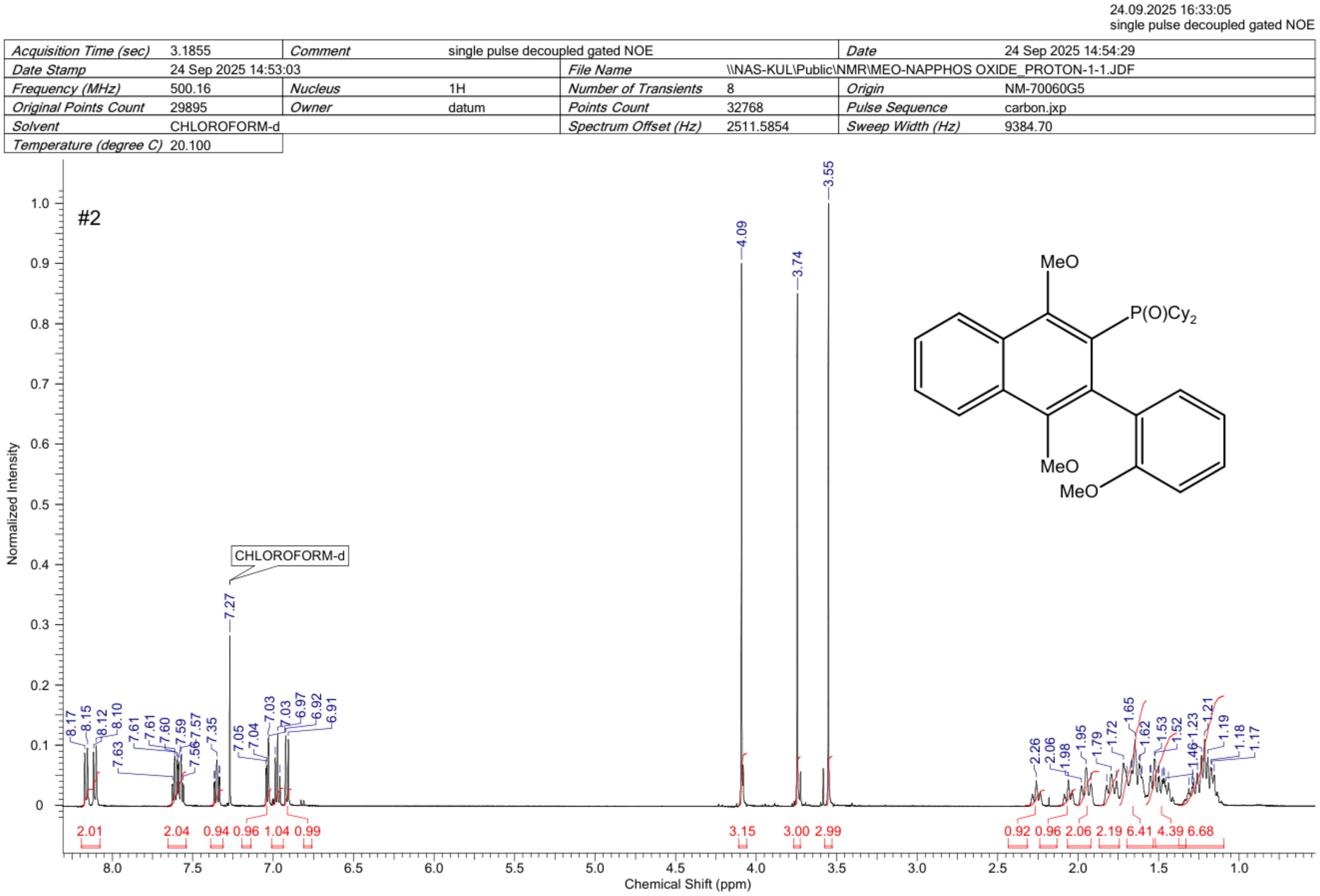

Figure S12. The ^{13}C NMR spectrum for compound **2**.

24.09.2025 16:36:49
single pulse decoupled gated NOE

Acquisition Time (sec)	0.7140	*Comment*	single pulse decoupled gated NOE			*Date*	24 Sep 2025 16:17:05
Date Stamp	24 Sep 2025 15:04:16			*File Name*	\\NAS-KUL\Public\NMR\MEO-NAPPHOS OXIDE_CARBON-1-1.JDF		
Frequency (MHz)	125.77	*Nucleus*	13C	*Number of Transients*	1451	*Origin*	NM-70060G5
Original Points Count	28074	*Owner*	datum	*Points Count*	32768	*Pulse Sequence*	carbon.jxp
Solvent	CHLOROFORM-d			*Spectrum Offset (Hz)*	12576.5293	*Sweep Width (Hz)*	39316.58
Temperature (degree C)	19.900						

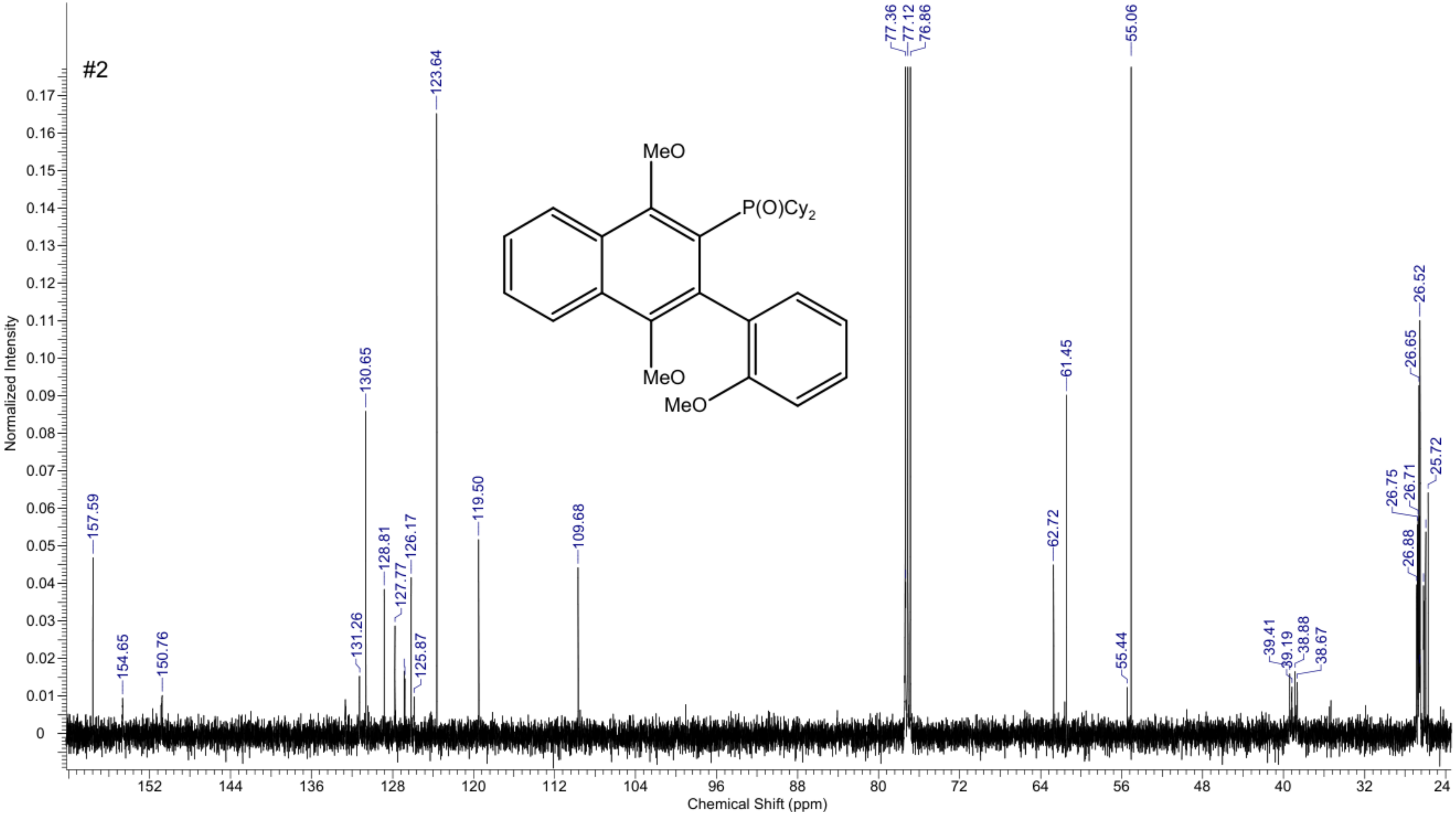

Figure S13. The ^{31}P NMR spectrum for compound **2**.

25.09.2025 11:07:45
5 mm PABBO BB-1H/D Z-GRD Z119470/0011

Acquisition Time (sec)	0.4020	*Comment*	5 mm PABBO BB-1H/D Z-GRD Z119470/0011			*Date*	14 Feb 2015 00:38:40
Date Stamp	14 Feb 2015 00:38:40			*File Name*	\\NAS-KUL\Public\NMR\MEO-NAPPHOS OXIDE_PHOSPHORUS-1.JDF		
Frequency (MHz)	202.45	*Nucleus*	31P	*Number of Transients*	32	*Origin*	spect
Original Points Count	32768	*Owner*	nmr	*Points Count*	32768	*Pulse Sequence*	zgpg30
Receiver Gain	2050.00	*SW(cyclical) (Hz)*	81521.73	*Solvent*	CHLOROFORM-d		
Spectrum Offset (Hz)	-9634.8818	*Spectrum Type*	STANDARD	*Sweep Width (Hz)*	81519.25	*Temperature (degree C)*	23.552

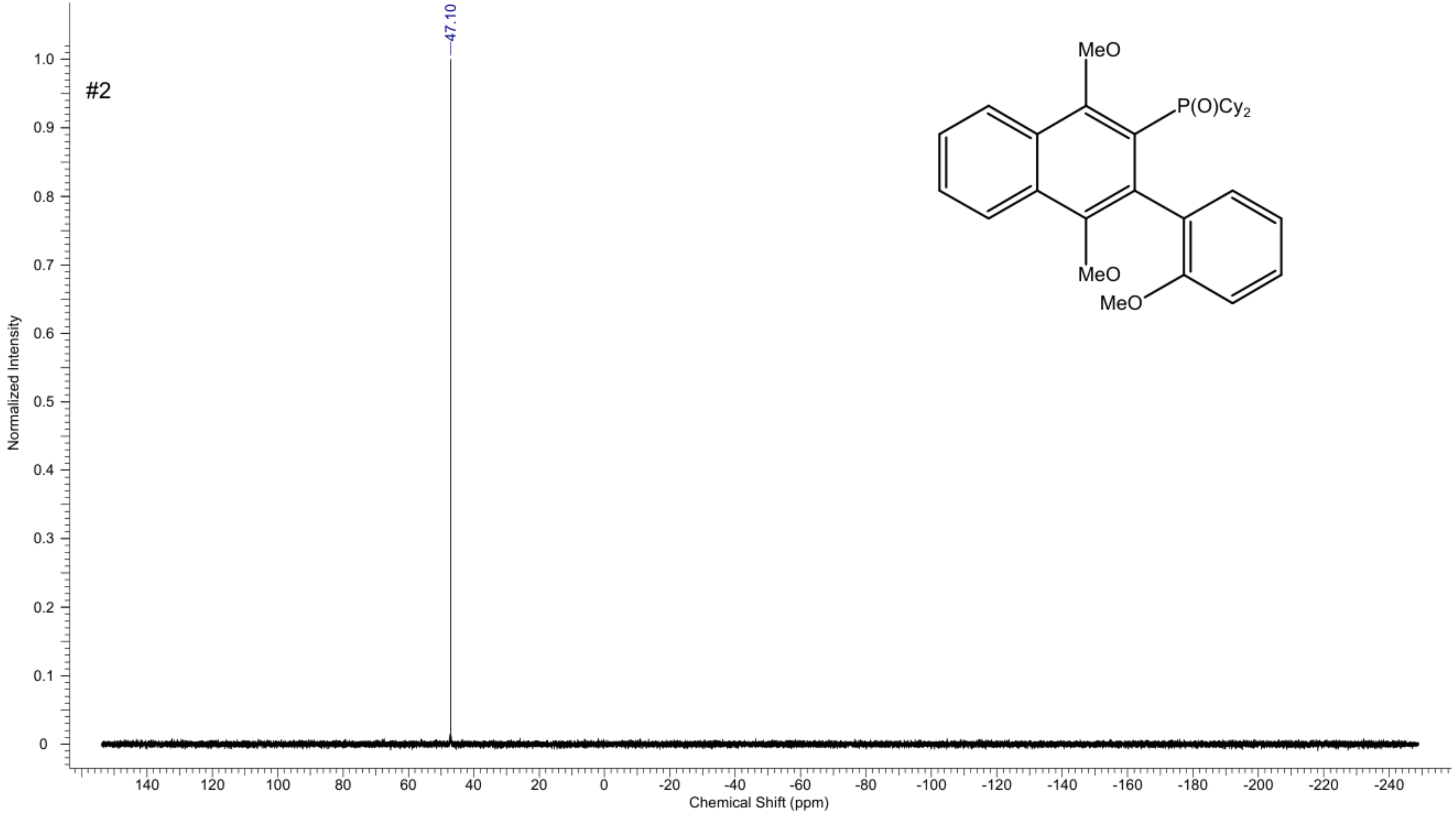

Figure S14. The ¹H NMR spectrum for compound **3**.

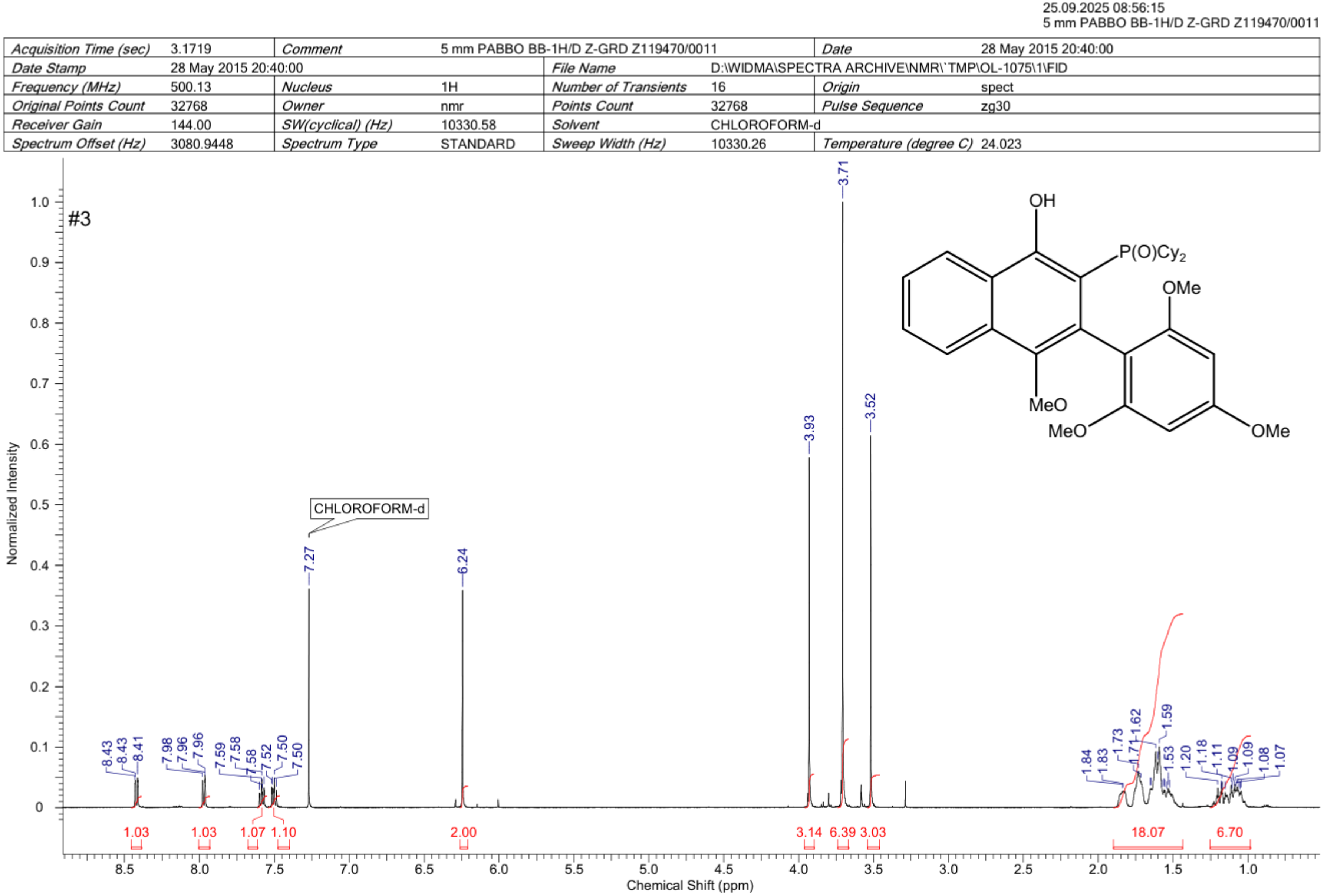

Figure S15. The ^{13}C NMR spectrum for compound **3**.

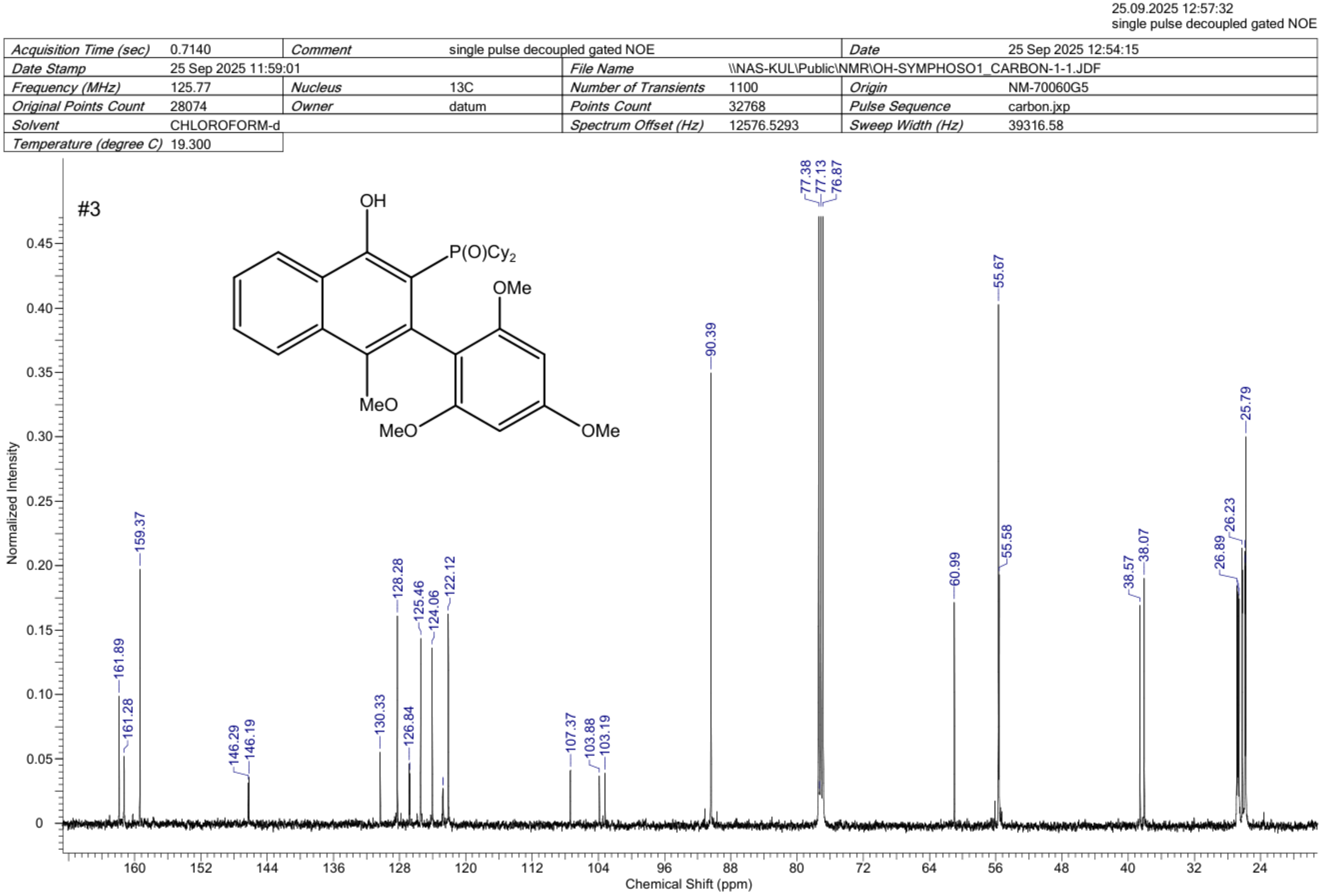

Figure S16. The ^{31}P NMR spectrum for compound **3**.

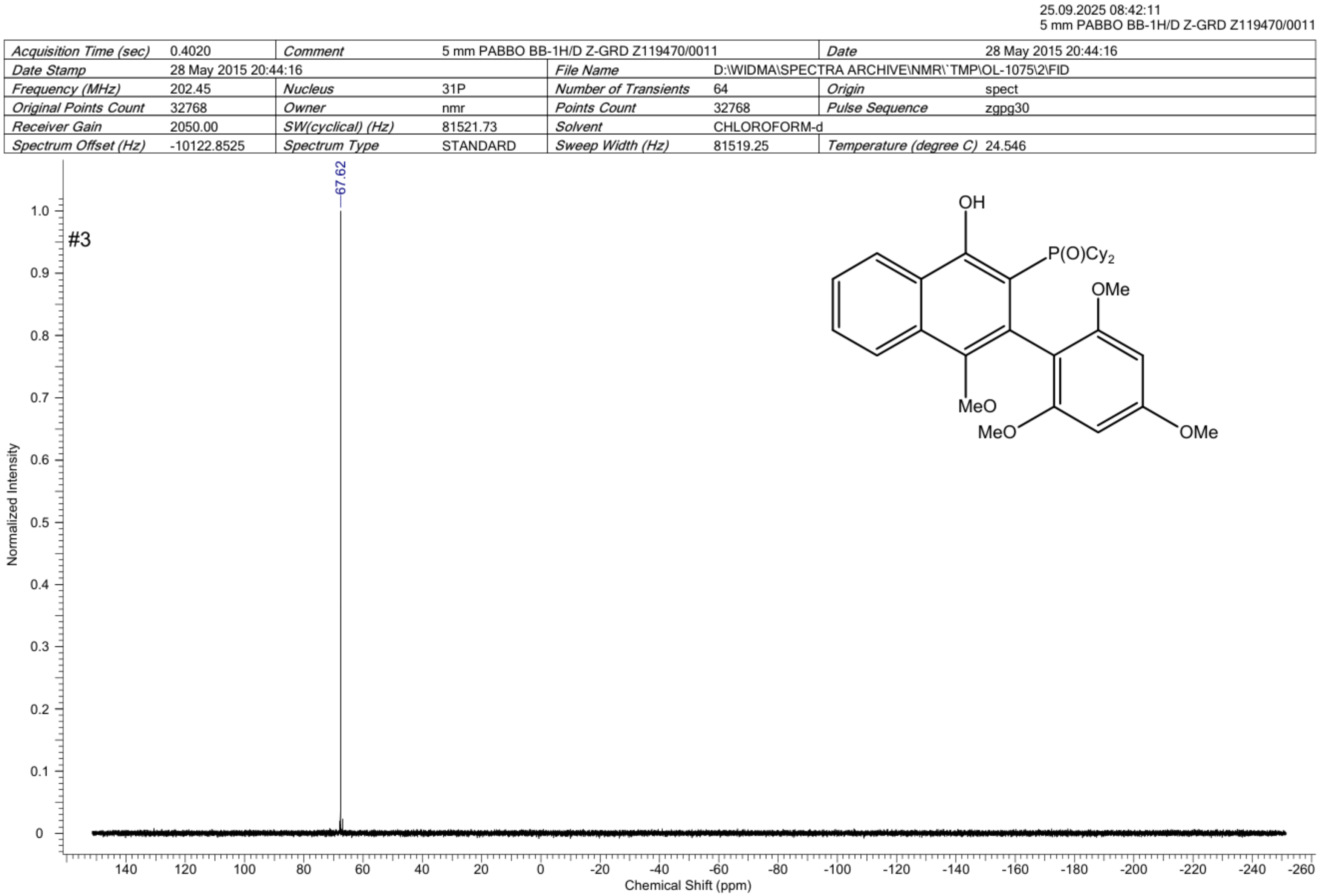

Figure S17. The ^{1}H NMR spectrum for compound **4**.

25.09.2025 12:36:23
1,4-dihydroxy-2-P(O)Cy2-3-An-naftalene

Acquisition Time (sec)	0.4915	*Comment*	1,4-dihydroxy-2-P(O)Cy2-3-An-naftalene	*Date*	20 Dec 2005 17:28:16
Date Stamp	20 Dec 2005 17:28:16	*File Name*	D:\WIDMA\NMR-(JUN23-DEC22)-2005\NMR-(JUN23-DEC22)\OLEG-DEC20-2005\12\FID		
Frequency (MHz)	400.30	*Nucleus*	1H	*Number of Transients*	16
Origin	av400				
Original Points Count	4096	*Owner*	NMR-guest	*Points Count*	4096
Pulse Sequence	zgig				
Receiver Gain	71.80	*SW(cyclical) (Hz)*	8333.33	*Solvent*	DMSO-d6
Spectrum Offset (Hz)	2501.1975				
Spectrum Type	STANDARD	*Sweep Width (Hz)*	8331.30	*Temperature (degree C)*	21.000

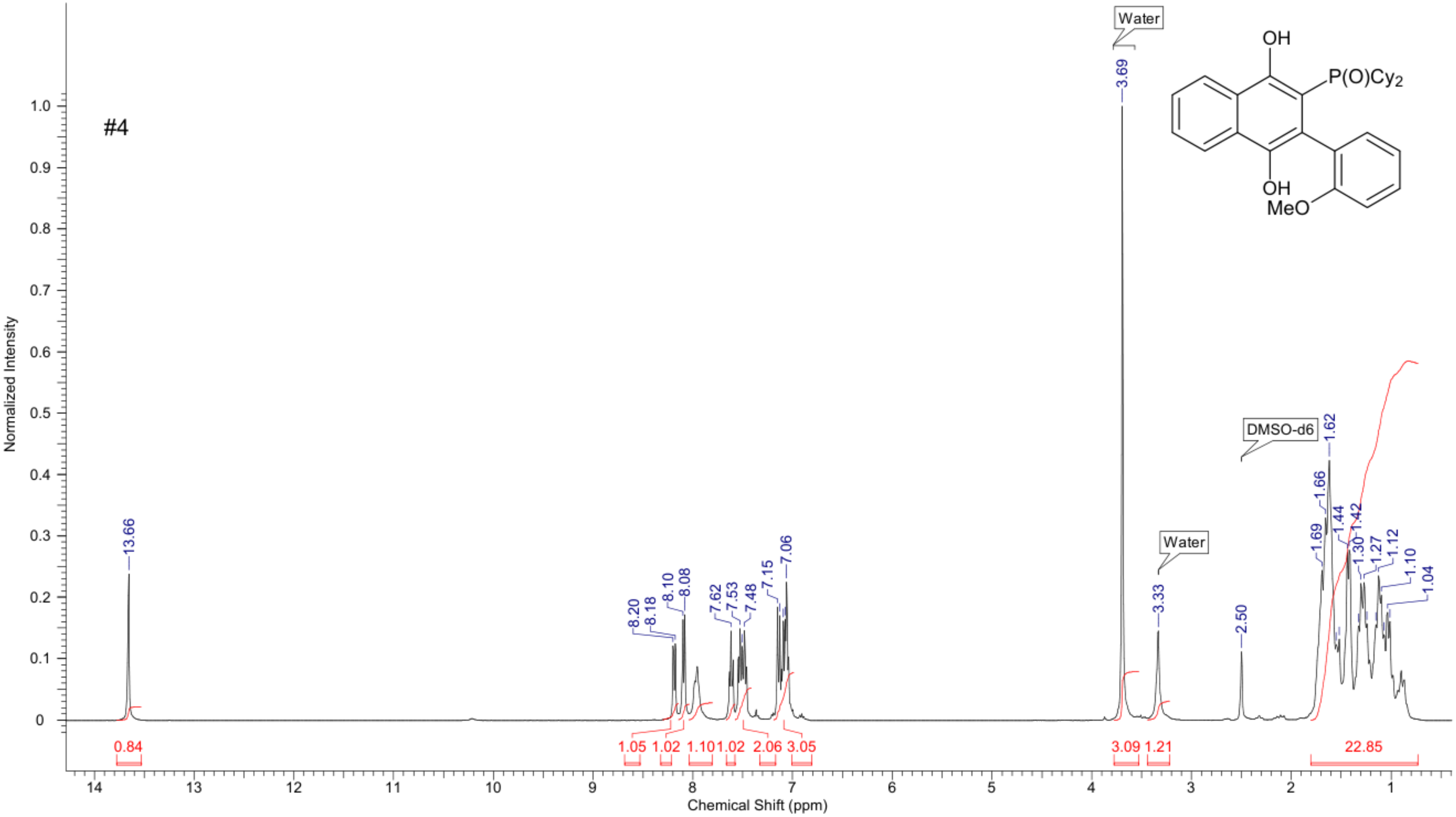

Figure S18. The ^{13}C NMR spectrum for compound **4**.

25.09.2025 12:38:18
1,4-dihydroxy-2-P(O)Cy2-3-An-naftalene

Acquisition Time (sec)	1.3664	*Comment*	1,4-dihydroxy-2-P(O)Cy2-3-An-naftalene			*Date*	20 Dec 2005 19:57:36
Date Stamp	20 Dec 2005 19:57:36			*File Name*	D:\WIDMA\NMR-(JUN23-DEC22)-2005\NMR-(JUN23-DEC22)\OLEG-DEC20-2005\16\FID		
Frequency (MHz)	100.66	*Nucleus*	13C	*Number of Transients*	2000	*Origin*	av400
Original Points Count	32768	*Owner*	NMR-guest	*Points Count*	32768	*Pulse Sequence*	dept135
Receiver Gain	16384.00	*SW(cyclical) (Hz)*	23980.81	*Solvent*	DMSO-d6	*Spectrum Offset (Hz)*	10065.6113
Spectrum Type	DEPT135	*Sweep Width (Hz)*	23980.08	*Temperature (degree C)*	20.900		

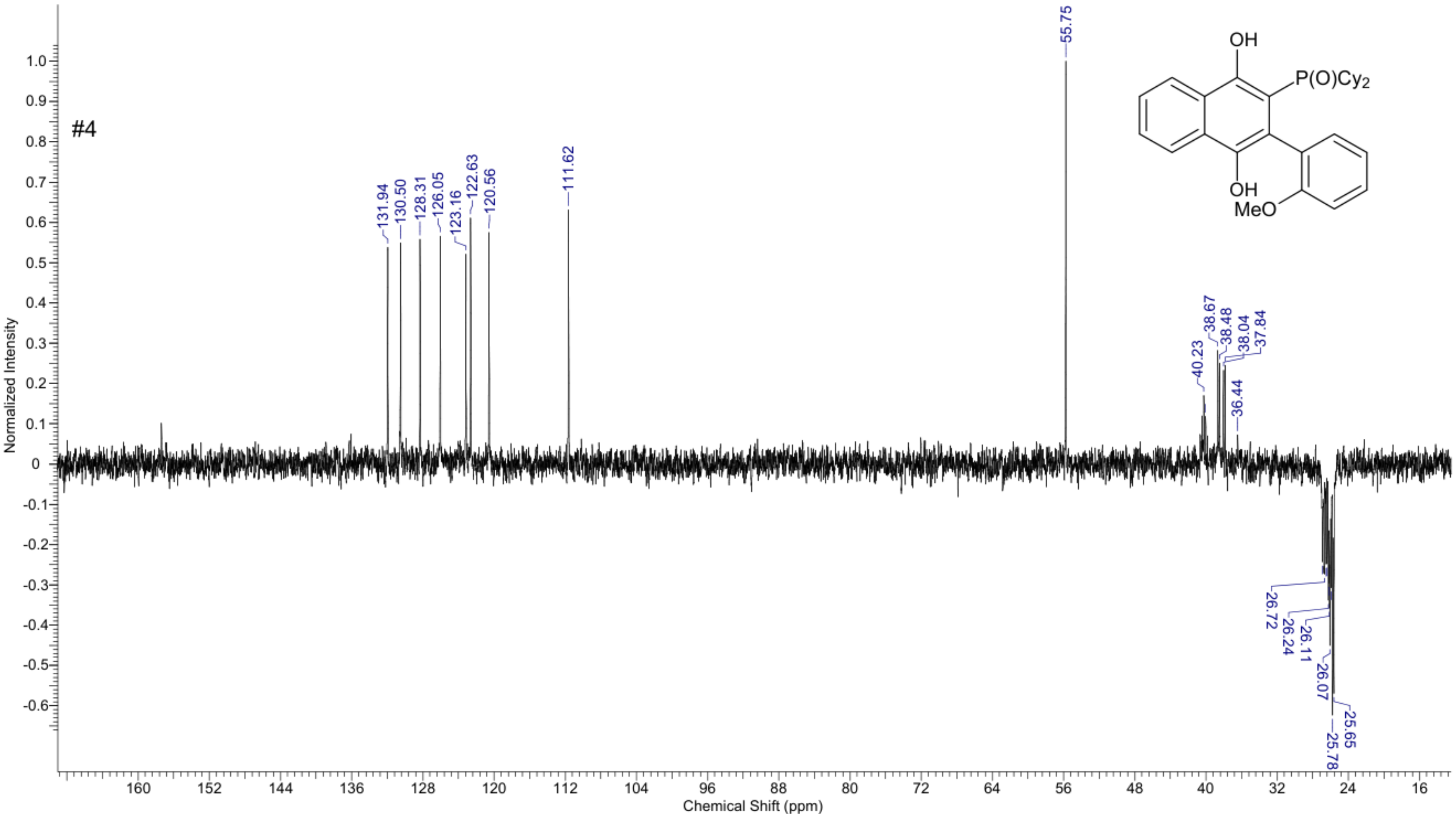

Figure S19. The ^{31}P NMR spectrum for compound **4**.

25.09.2025 12:38:33
1,4-dihydroxy-2-P(O)Cy2-3-An-naftalene

Acquisition Time (sec)	0.5046	*Comment*	1,4-dihydroxy-2-P(O)Cy2-3-An-naftalene			*Date*	20 Dec 2005 22:03:28
Date Stamp	20 Dec 2005 22:03:28			*File Name*	D:\WIDMA\NMR-(JUN23-DEC22)-2005\NMR-(JUN23-DEC22)\OLEG-DEC20-2005\19\FID		
Frequency (MHz)	162.04	*Nucleus*	31P	*Number of Transients*	256	*Origin*	av400
Original Points Count	32768	*Owner*	NMR-guest	*Points Count*	32768	*Pulse Sequence*	zg30
Receiver Gain	18390.40	*SW(cyclical) (Hz)*	64935.07	*Solvent*	DMSO-d6	*Spectrum Offset (Hz)*	8102.2236
Spectrum Type	STANDARD	*Sweep Width (Hz)*	64933.09	*Temperature (degree C)*	20.900		

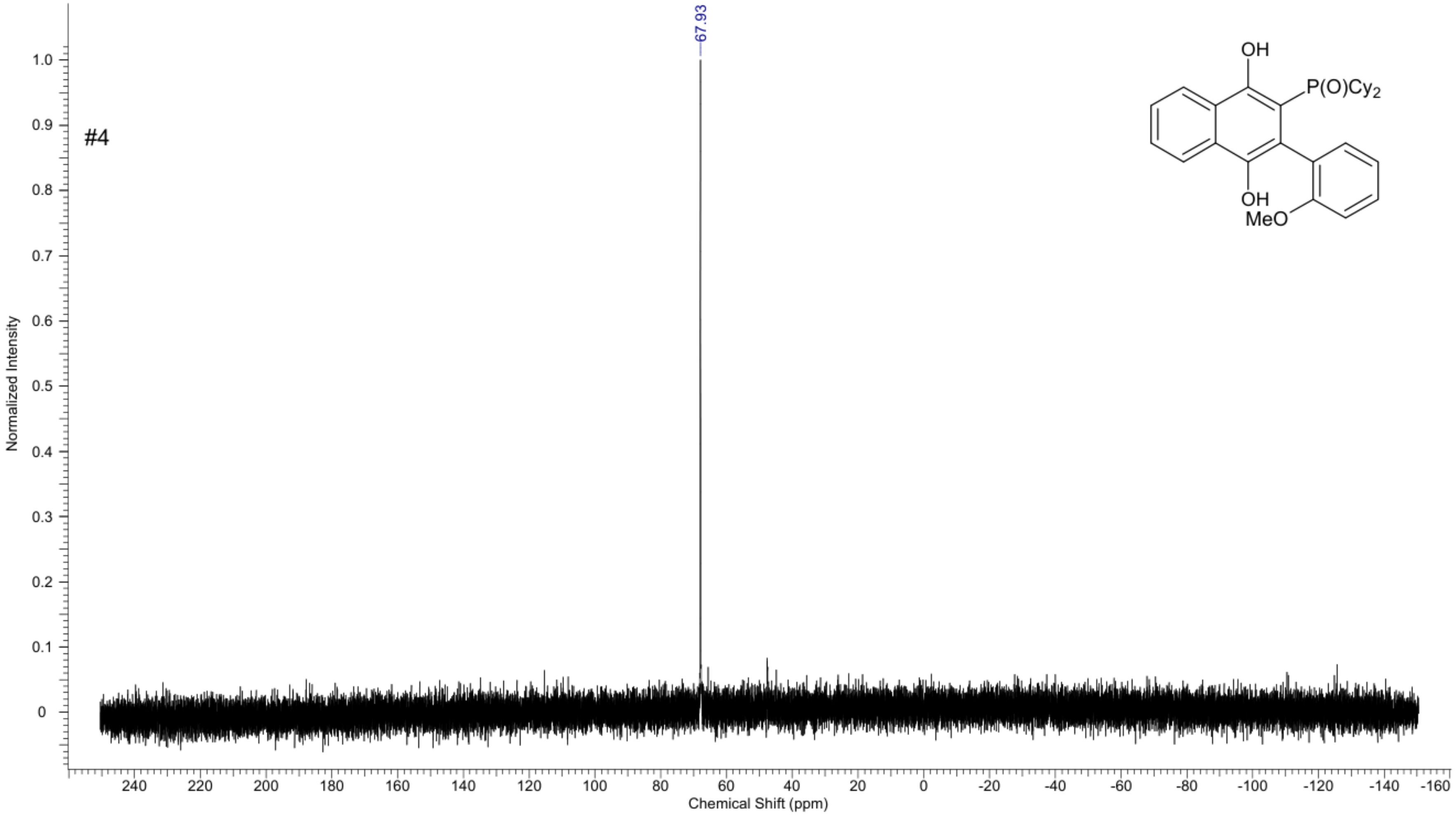

Figure S20. The ¹H NMR spectrum for compound **5**.

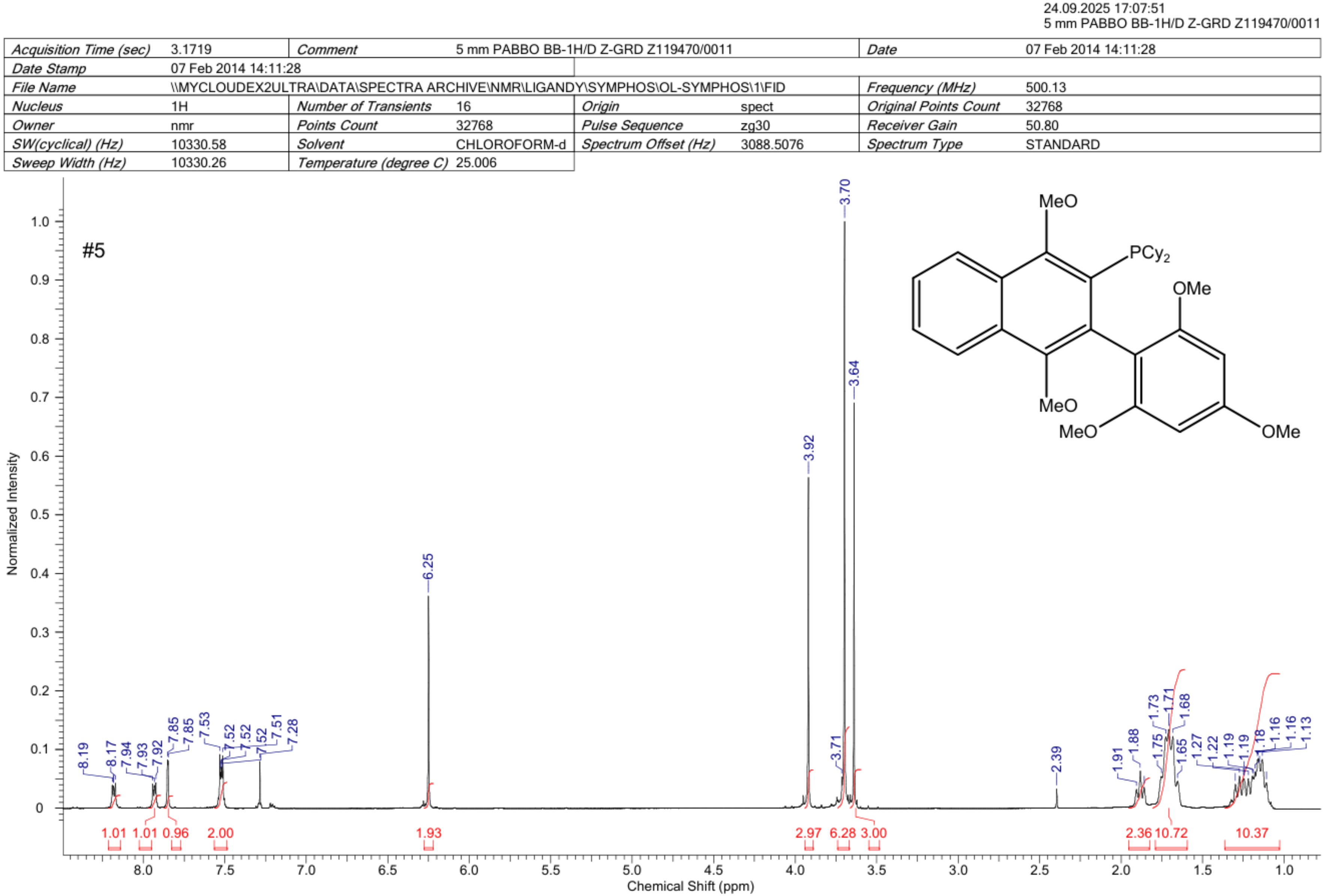

Figure S21. The ^{13}C NMR spectrum for compound **5**.

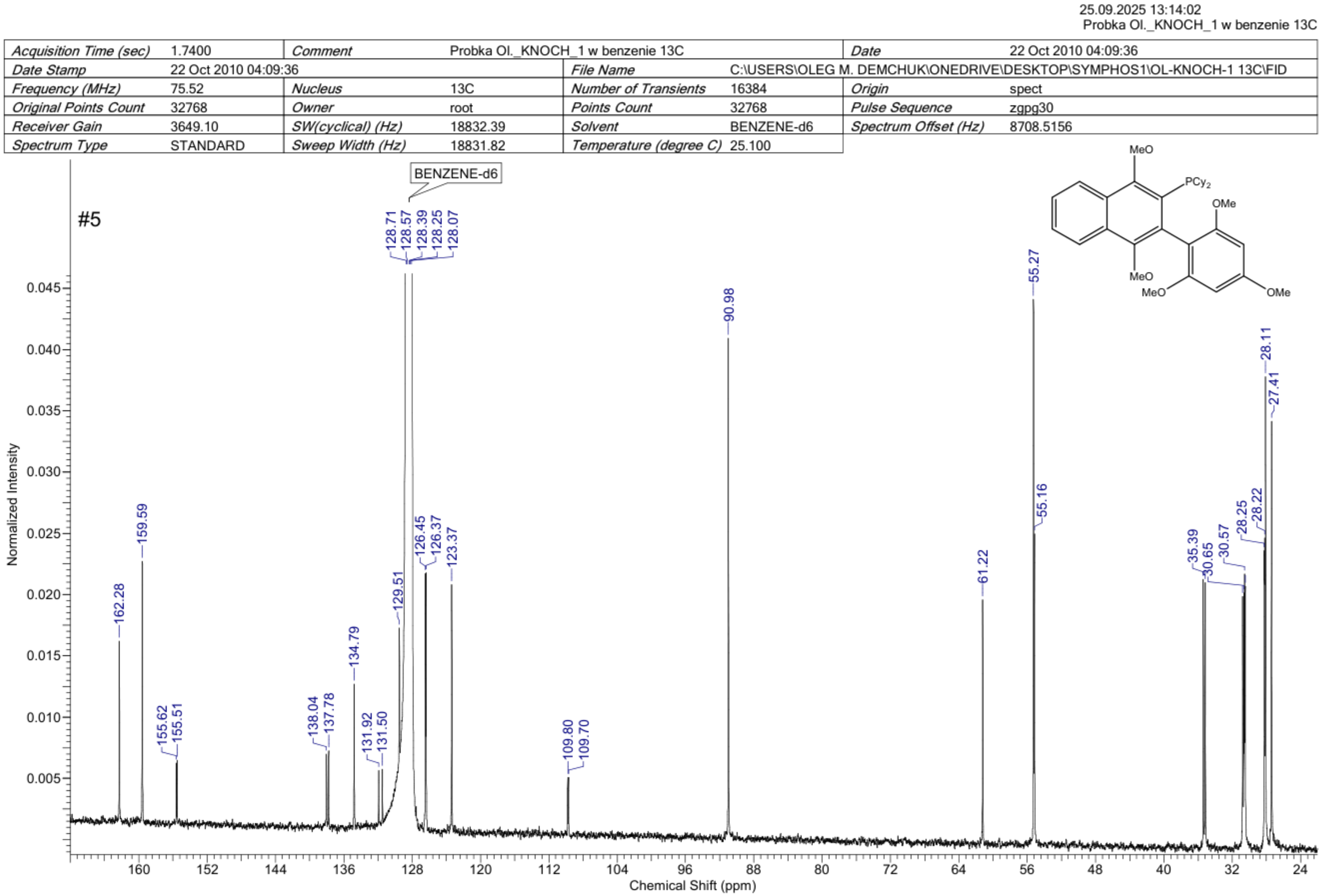

Figure S22. The ^{31}P NMR spectrum for compound **5**.

24.09.2025 17:08:06

Acquisition Time (sec)	0.4020	*Date*	22 Jul 2014 13:48:16			*Date Stamp*	22 Jul 2014 13:48:16
File Name	\\MYCLOUDEX2ULTRA\DATA\SPECTRA ARCHIVE\NMR\LIGANDY\SYMPHOS\OL-SYMPHOS\2\FID					*Frequency (MHz)*	202.45
Nucleus	31P	*Number of Transients*	16	*Origin*	spect	*Original Points Count*	32768
Owner	nmr	*Points Count*	32768	*Pulse Sequence*	zgpg30	*Receiver Gain*	2050.00
SW(cyclical) (Hz)	81521.73	*Solvent*	CHLOROFORM-d			*Spectrum Offset (Hz)*	-10122.8525
Spectrum Type	STANDARD	*Sweep Width (Hz)*	81519.25	*Temperature (degree C)*	23.664		

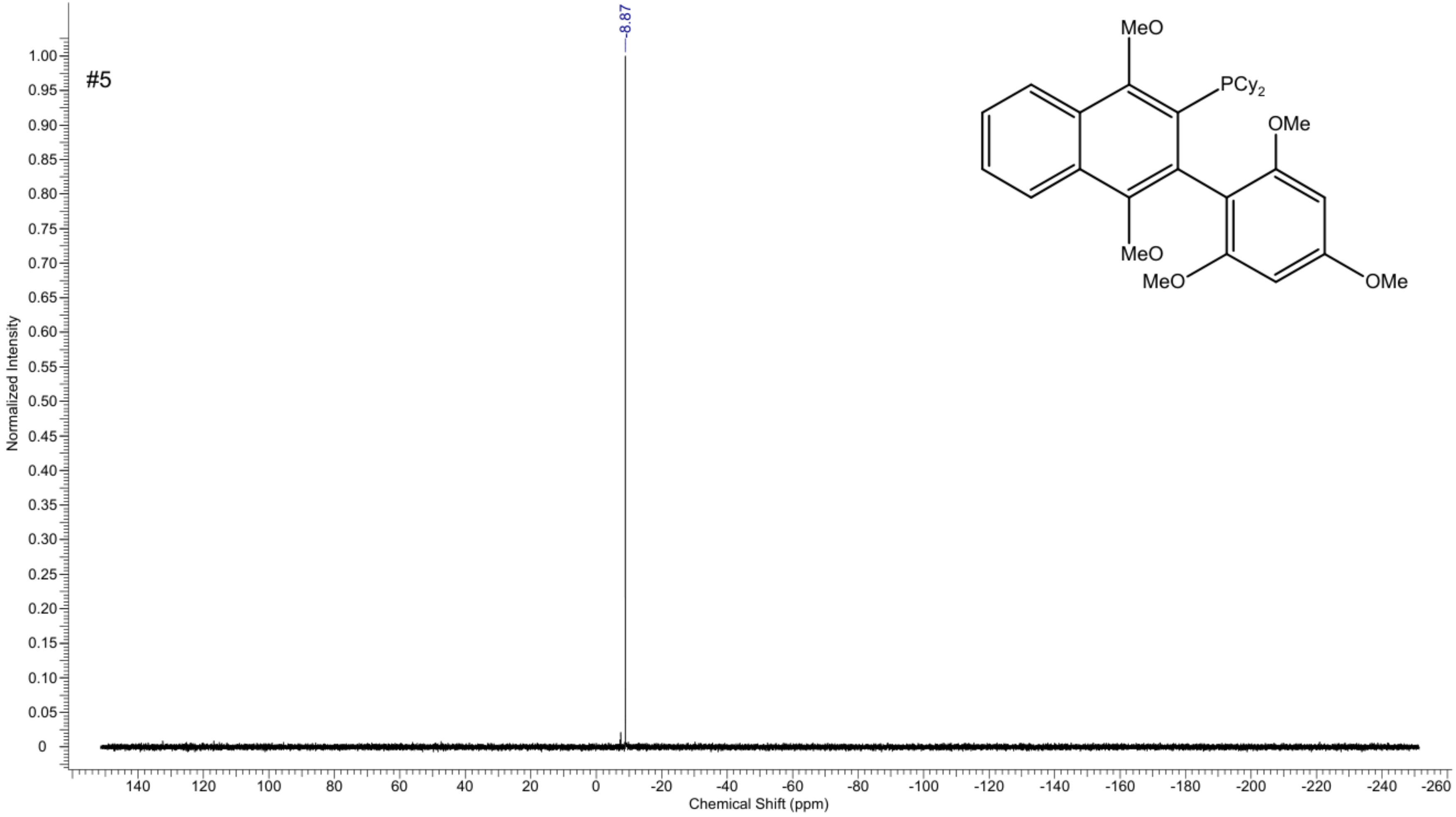

Supplement: Supplementary file 1 [file molecules-30-04018-s001.zip › Supplementary Materials.pdf]
