# Supplementary material for: Aromaticity Tuning in Biaryl Monophosphines and Their Derivatives
Source: Molecules. 2025 Oct 8;30(19):4018. doi: 10.3390/molecules30194018 (PMC12525867; doi:10.3390/molecules30194018)

checkCIF/PLATON report

You have not supplied any structure factors. As a result the full set of tests cannot be run.

THIS REPORT IS FOR GUIDANCE ONLY. IF USED AS PART OF A REVIEW PROCEDURE FOR PUBLICATION, IT SHOULD NOT REPLACE THE EXPERTISE OF AN EXPERIENCED CRYSTALLOGRAPHIC REFEREE.

No syntax errors found. CIF dictionary Interpreting this report

Datablock: 3

Bond precision: C-C = 0.0033 A Wavelength=1.54184

Cell:	a=8.80010(17)	b=18.3104(3)	c=18.3194(3)
	alpha=90	beta=90.2353(17)	gamma=90
Temperature:	294 K		

	Calculated	Reported
Volume	2951.84(9)	2951.84(10)
Space group	P 21/c	P 1 21/c 1
Hall group	-P 2ybc	-P 2ybc
Moiety formula	C32 H41 O6 P	C32 H41 O6 P
Sum formula	C32 H41 O6 P	C32 H41 O6 P
Mr	552.62	552.65
Dx,g cm-3	1.243	1.243
Z	4	4
Mu (mm-1)	1.166	1.166
F000	1184.0	1188.8
F000'	1188.56	
h,k,lmax	11,23,23	11,23,23
Nref	6178	6065
Tmin,Tmax	0.869,0.911	0.677,1.000
Tmin'	0.747	

Correction method= # Reported T Limits: Tmin=0.677 Tmax=1.000
AbsCorr = MULTI-SCAN

Data completeness= 0.982 Theta(max)= 76.300

R(reflections)= 0.0488(4785) wR2(reflections)= 0.1421(6065)

S = 1.031 Npar= 359

The following ALERTS were generated. Each ALERT has the format
test-name_ALERT_alert-type_alert-level.
Click on the hyperlinks for more details of the test.

Alert level B

```
PLAT230_ALERT_2_B Hirshfeld Test Diff for    C5       --C6       .        8.0 s.u.
```

Alert level G

```
PLAT005_ALERT_5_G No Embedded Refinement Details Found  in the CIF     Please Do !
PLAT068_ALERT_1_G Reported F000 Differs from Calcd (or Missing)...     Please Check
PLAT769_ALERT_4_G CIF Embedded Explicitly Supplied Scattering Data          4 Note
PLAT948_ALERT_5_G Externally Supplied Scattering Factors       CIF          4 Note
PLAT982_ALERT_1_G The  C-f'=    0.0192 Deviates from  IT-Value =       0.0181 Check
PLAT982_ALERT_1_G The  O-f'=    0.0524 Deviates from  IT-Value =       0.0492 Check
PLAT982_ALERT_1_G The  P-f'=    0.3036 Deviates from  IT-Value =       0.2955 Check
PLAT983_ALERT_1_G The  O-f"=    0.0338 Deviates from  IT-Value =       0.0322 Check
PLAT983_ALERT_1_G The  P-f"=    0.4370 Deviates from  IT-Value =       0.4335 Check
```

0 **ALERT level A** = Most likely a serious problem - resolve or explain
1 **ALERT level B** = A potentially serious problem, consider carefully
0 **ALERT level C** = Check. Ensure it is not caused by an omission or oversight
9 **ALERT level G** = General information/check it is not something unexpected

6 ALERT type 1 CIF construction/syntax error, inconsistent or missing data
1 ALERT type 2 Indicator that the structure model may be wrong or deficient
0 ALERT type 3 Indicator that the structure quality may be low
1 ALERT type 4 Improvement, methodology, query or suggestion
2 ALERT type 5 Informative message, check

It is advisable to attempt to resolve as many as possible of the alerts in all categories. Often the minor alerts point to easily fixed oversights, errors and omissions in your CIF or refinement strategy, so attention to these fine details can be worthwhile. In order to resolve some of the more serious problems it may be necessary to carry out additional measurements or structure refinements. However, the purpose of your study may justify the reported deviations and the more serious of these should normally be commented upon in the discussion or experimental section of a paper or in the "special_details" fields of the CIF. checkCIF was carefully designed to identify outliers and unusual parameters, but every test has its limitations and alerts that are not important in a particular case may appear. Conversely, the absence of alerts does not guarantee there are no aspects of the results needing attention. It is up to the individual to critically assess their own results and, if necessary, seek expert advice.

Publication of your CIF in IUCr journals

A basic structural check has been run on your CIF. These basic checks will be run on all CIFs submitted for publication in IUCr journals (*Acta Crystallographica*, *Journal of Applied Crystallography*, *Journal of Synchrotron Radiation*); however, if you intend to submit to *Acta Crystallographica Section C* or *E* or *IUCrData*, you should make sure that full publication checks are run on the final version of your CIF prior to submission.

Publication of your CIF in other journals

Please refer to the *Notes for Authors* of the relevant journal for any special instructions relating to CIF submission.

PLATON version of 04/06/2025; check.def file version of 30/05/2025

Datablock 3 - ellipsoid plot

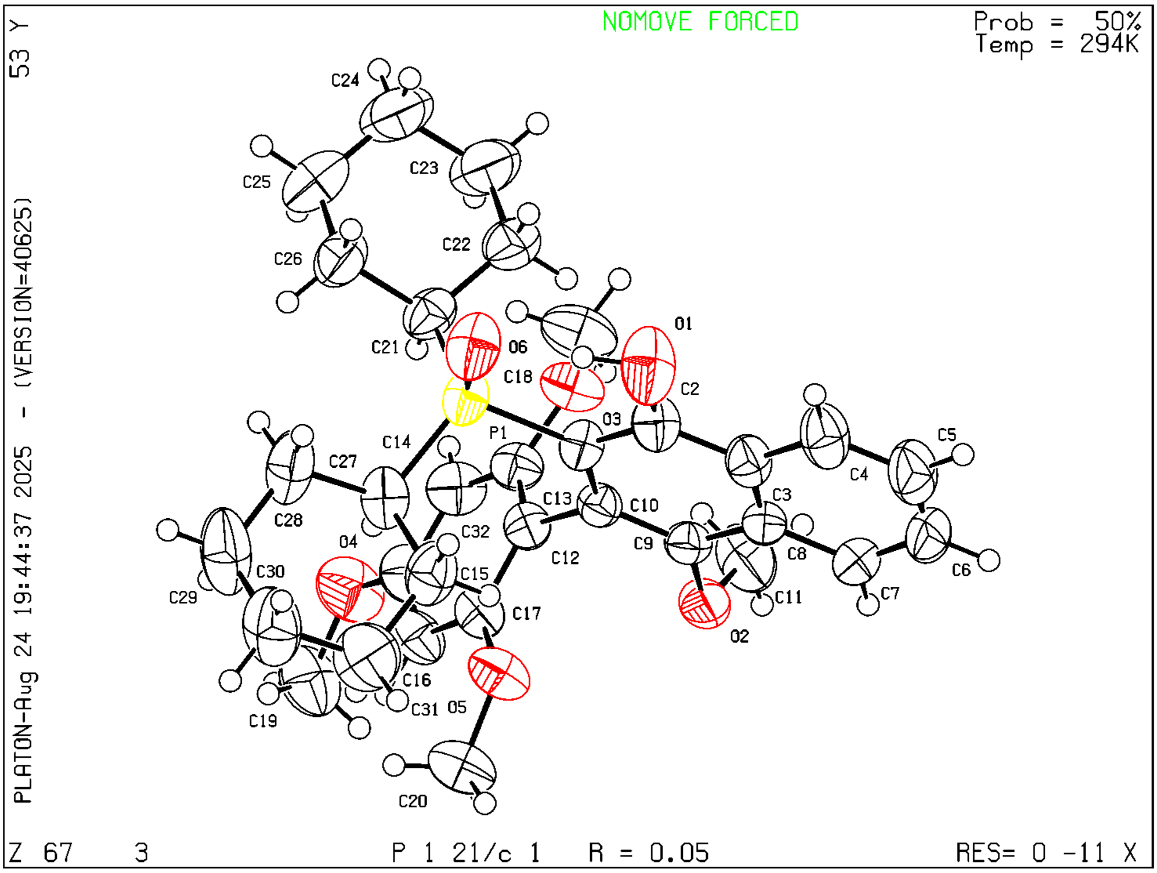

Supplement: Supplementary file 1 [file molecules-30-04018-s001.zip › File S1.zip!/cif-files/3_checkcif.pdf]
